# PARA: A one-meter reach, two-kg payload, three-DoF open source robotic arm with customizable end effector

**DOI:** 10.1016/j.ohx.2021.e00209

**Published:** 2021-06-17

**Authors:** Albert Tai, Michael Chun, Yuqiu Gan, Mert Selamet, Hod Lipson

**Affiliations:** Creative Machines Lab, Columbia University School of Engineering and Applied Science, New York, NY, United States

**Keywords:** Hardware, Mechanics, Robotic arm, Robotics, Additive manufacturing, 3D printing

## Abstract

This article presents a robotic arm that is more cost effective than existing models on the market while still maintaining sufficient torque and speed capabilities. Most industrial-grade high power and precision arms are often very expensive, while conversely, more low-cost arms targeted toward the education and hobby sectors are inadequate in power and robustness. The Creative Machines Lab’s three degree of freedom Printed Articulated Robotic Arm (PARA) can lift a 2 kg payload at a reach of 940 mm, while under a no-load case, it has exhibited a precision of about ±2.6 mm at an end effector speed of 250 mm/s. It costs about $3400 to build, an order of magnitude lower than market models with similar functionalities. This project is also meant to serve as a demonstration of the usage of 3D printed parts as practical tools in industry.

## Graphical abstract

**Figure:**
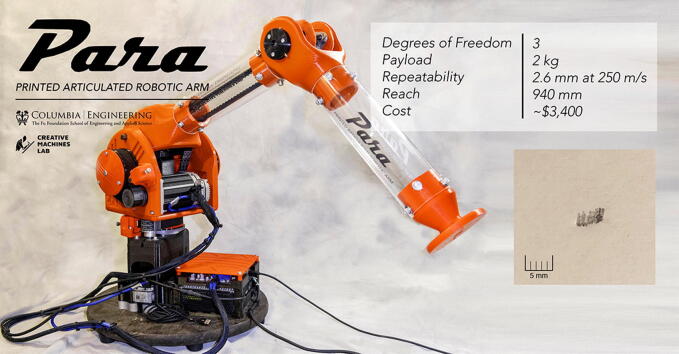

## 1 Hardware in context

PARA is a three degree of freedom robotic arm which can lift approximately 2 kg at a 940 mm reach. The primary goal of the PARA project was to create a mostly 3D printed open-source robotic arm that is more cost effective than similar-scale proprietary arms on the market while still maintaining sufficient torque, speed, and precision capabilities. Many existing open-source robotic arms and low-cost arms targeted towards the education and hobby sectors are often inadequate in size and power. Additionally, their designs and functionalities are often poorly documented so they cannot be easily replicated. By being bigger and stronger than the average open-source arm and cheaper than many industrial-grade proprietary models, PARA fills a niche for multiple sectors. Amateur or small-scale manufacturers can use PARA for simple manual operations like pick-and-place which do not require the extreme amounts of precision associated with industrial models and their high price markups. With custom end effector attachments, PARA can also be used for tasks requiring tools such as drilling, screwing, or applying paint or adhesive. Researchers and educators can use PARA as a testbed for their own creations like custom end effectors or new control schema, or just use it as an educational tool. The readily-available CAD files and build instructions included in this article encourage users to customize and/or improve PARA’s designs for their own purposes. Finally, being primarily 3D printed, PARA also serves as a demonstration of the potential of additive manufacturing in advanced applications.

**Table:** 

**Specifications table:**	
**Hardware name**	*Printed Articulated Robotic Arm (PARA)*
**Subject area**	*Educational Tools and Open Source Alternatives to Existing Infrastructure*
**Hardware type**	*Mechanical engineering and materials science*
**Open source license**	*MIT*
**Cost of hardware**	$*3400*
**Source file repository**	https://doi.org/10.17605/OSF.IO/5AF4V

PARA strives to achieve a balance between functionality (with regards to power and precision) and affordability. In order to assess its specifications relative to other existing robotic arm models, 32 other existing commercial or open-source robotic arms were assessed, comparing parameters such as payload, reach, repeatability, and cost.

**Fig. 1:**
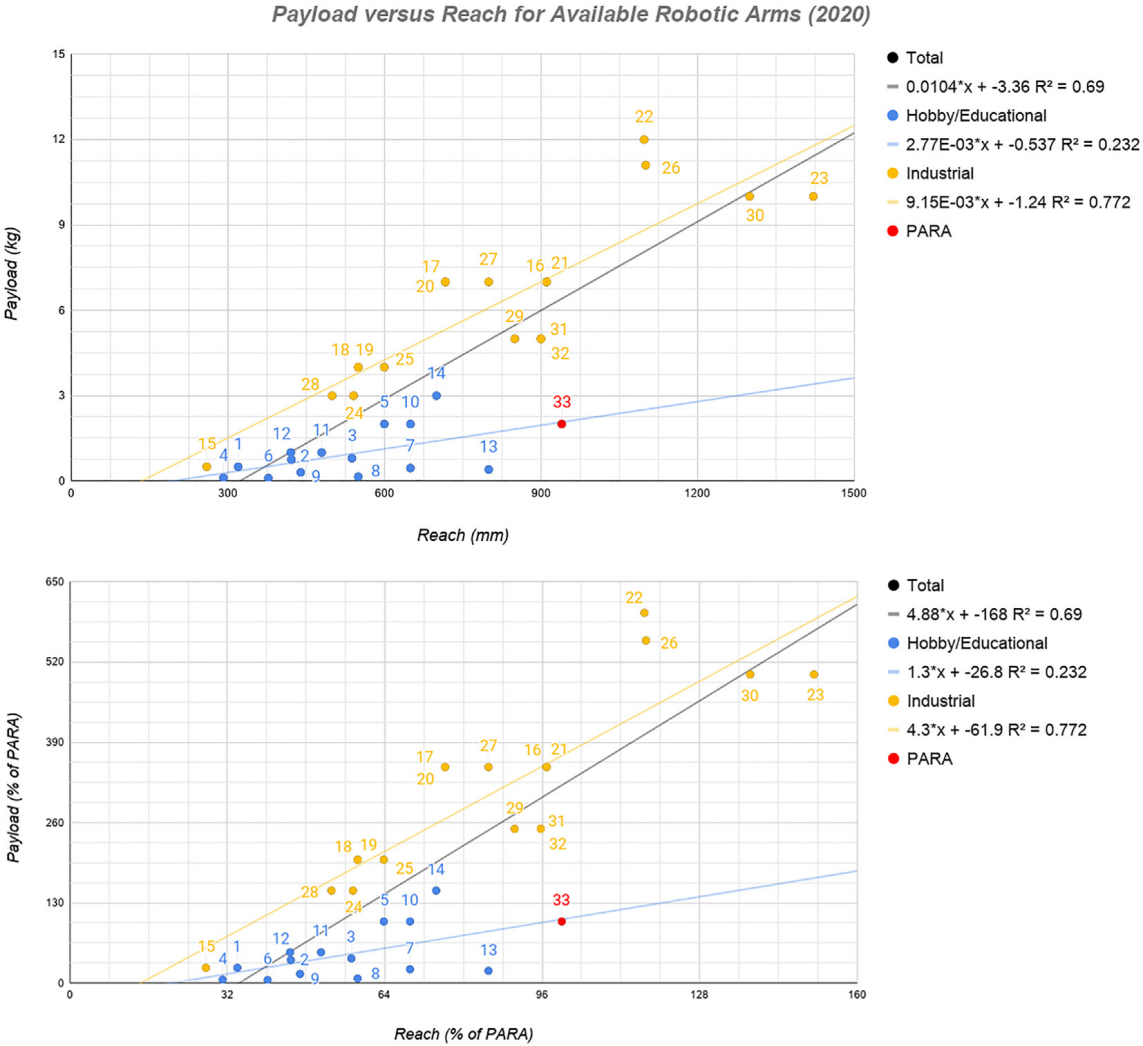
Payload versus reach for available robotic arms (2020) in terms of absolute values (top) and percentages of PARA’s values (bottom). See Appendix for individual robot information corresponding to labeled points.

**Fig. 2:**
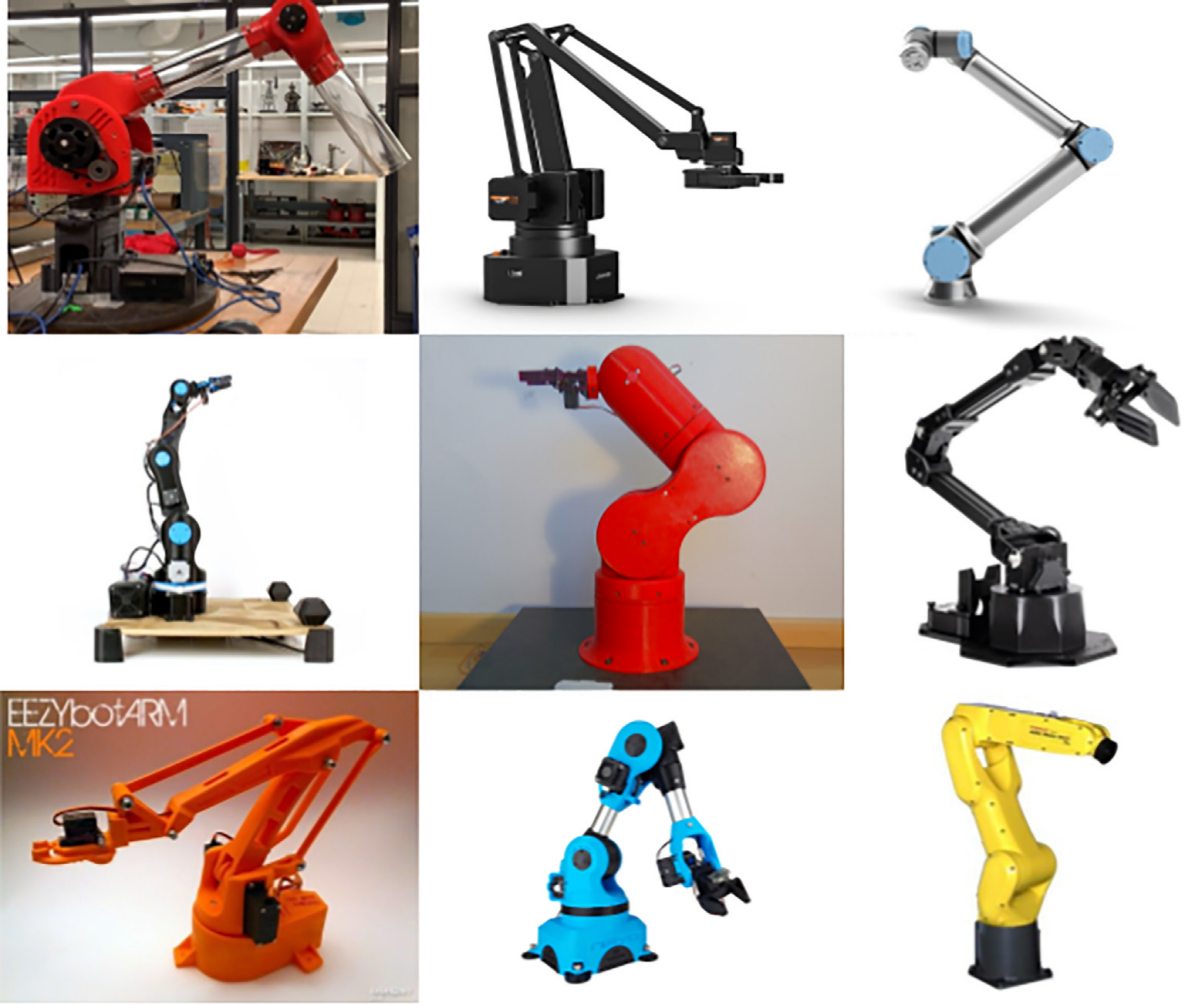
The diversity of existing robotic arms. Top row (left to right): Columbia CML PARA, Ufactory UArm Swift [6], Universal Robotics UR3e [7]. Middle row: BCN3D Moveo [1], Thor [8], Trossen Robotics ViperX 250 [3]. Bottomw row: EEZY Robots EEZYbotArm v2 [9], Fanuc LR Mate 200iD [10], Oz Robtoics Niryo One [11].

Fig. 1 plots payload versus load-lifting capabilities for various robotic arms. These arms (the diversity of which can be seen in Fig. 2) were categorized into two groups: arms intended for hobby/educational purposes and arms intended for industrial tasks like welding or heavy lifting. From observing the trendlines for both categories of arms, one can observe the clear difference in expected load-lifting capabilities between the two categories, with PARA, touting a maximum payload of 2 kg at a 940 mm reach, falling solidly on the blue trendline for hobby/educational arms. Observing Fig. 1, one can easily observe the novelty of PARA’s scale: its reach and maximum payload outclass most arms in the hobby/educational category. Many 3D printed arms with freely available parts on the Internet have shorter reach and lower load-bearing capabilities because of the smaller motors that they utilize. PARA circumvents these shortcomings by implementing powerful motors and utilizing large, robustly-designed printed parts. For example, the BCN3D Moveo, a popular open-source model, uses NEMA 17 Steppers with an approximately 0.6 Nm output [1]. Trossen Robotics’ Interbotix arms use Dynamixel XM-series motors [2], [3], which while precise, feature stall torques less than 5 Nm [4]. In contrast, PARA’s NEMA 34 motors have a 13 Nm output.

Conversely, industrial strength robotic arms are often capable of lifting much greater loads at similar reaches due to their usage of metallic components and custom motors/gearboxes. These higher-end components come at the expense of cost: industrial-grade robotic arms at the similar size class offered by major manufacturers such as FANUC, KUKA, or Universal Robotics often cost tens of thousands of dollars [5], and are not affordable for many. A KUKA sales representative provided the following approximate costs for several KUKA industrial robotic arms similar in size to PARA (M. Robey, personal communication, January 19, 2019):

**Table:** 

**KUKA Model**	**Reach (mm)**	**Payload Capacity (kg)**	**Payload Capacity (% of PARA)**	**Approx. Cost (USD)**	**Approx. Cost (% of PARA)**
Agilus KR 3 R540	541	3	150	24,000	700
Agilus KR 4 R600	600	4	200	23,000	680
Agilus KR 10 R1100-2	1101	11	550	33,000	970
LBR iiwa 7 R800	800	7	350	77,000	2260

For many, equally as important as strength and functionality is affordability: PARA achieves cost reduction compared to proprietary market models by using relatively cheap parts and being easy to assemble. The arm’s parts are primarily 3D printed, meaning the same (often inexpensive) machine can produce a majority of the parts, rather than relying on distinct tooling for each individual component. The usage of plastic parts also presents cost-savings when compared to metal. Parts which cannot be printed such as fasteners and bearings are readily available on the Internet. Most importantly, PARA is open-source and the hardware is not driven by a necessity for profit: for example, users skip assembly costs by assembling PARA themselves. The arm is designed to be manufactured with minimal fabrication effort: the only instances of manual fabrication are drilling the arm’s base plate and linkage body tubes, sawing the linkage body tubes, and heat-setting several brass inserts into 3D printed parts. Additionally, users can customize or even improve the open-source arm to serve their own needs.

PARA currently costs about $3400 to build, but this price point could be further lowered by replacing the arm’s Clearpath servo motors ($713 and $631) – which are expensive due to their sophisticated internal control functionalities – with standard NEMA 34 and 23 stepper motors and encoders. If each stepper motor costs approximately $100, PARA’s cost would be driven down to around $1500.

**Fig. 3:**
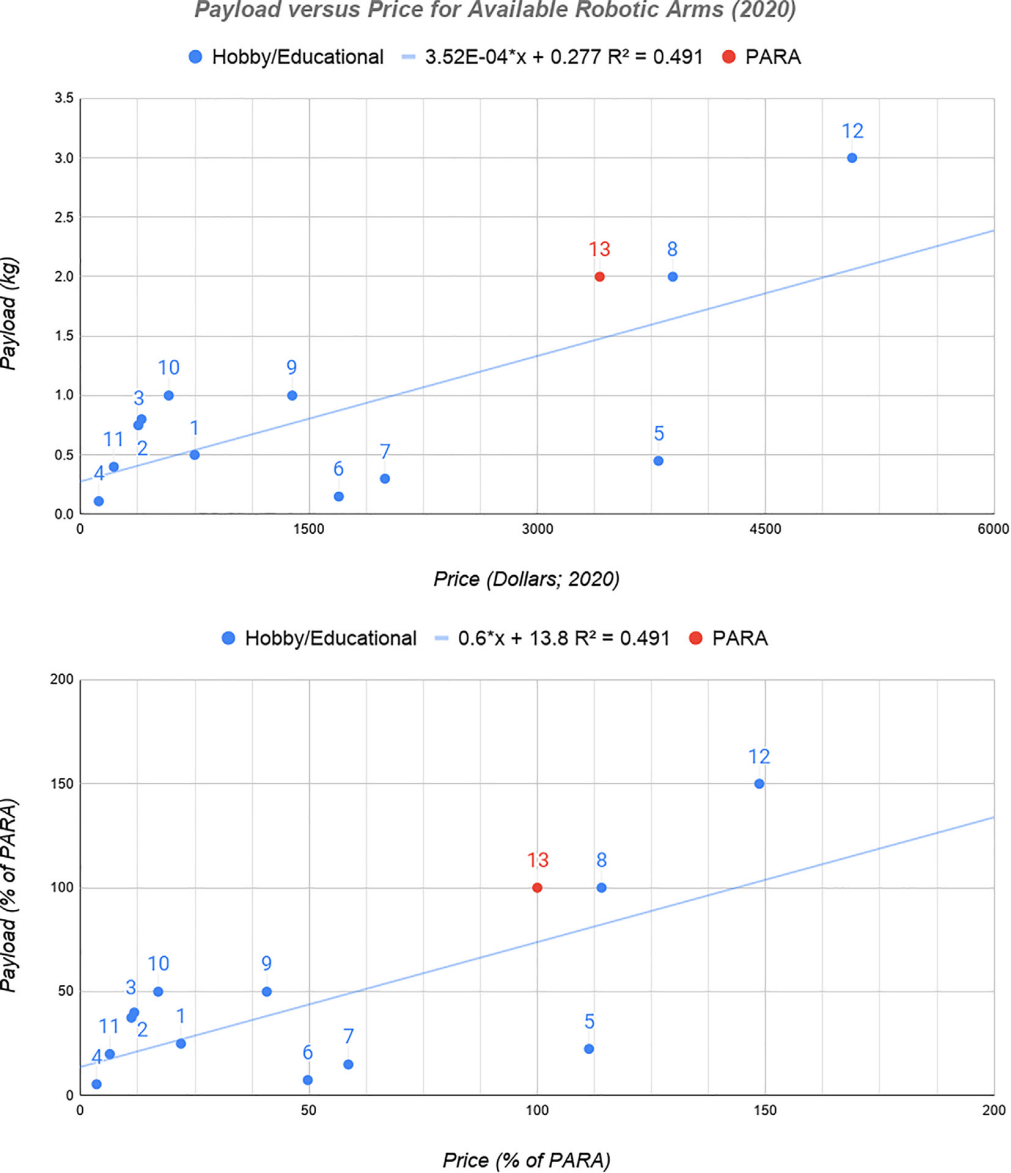
Payload versus cost for available robotic arms (2020) in terms of absolute values (top) and percentages of PARA’s values (bottom). See Appendix for individual robot information corresponding to labeled points.

Nonetheless, compared to other non-industrial models, PARA features greater load lifting capabilities per dollar. As previously stated, industrial robotic arms are often capable of lifting 2–3 times PARA’s max payload, however their price point is an order of magnitude greater. For a more apples-to-apples comparison, Fig. 3 plots load lifting capabilities versus price for just the hobby/educational grade robotic arms researched in this study.

At around $3400 to build, PARA can lift 0.59 grams per dollar, approximately 33% higher than a robot of identical cost if it followed the observed trendlines from Fig. 3. This advantage can be further increased by replacing the arm’s expensive motors.

**Fig. 4:**
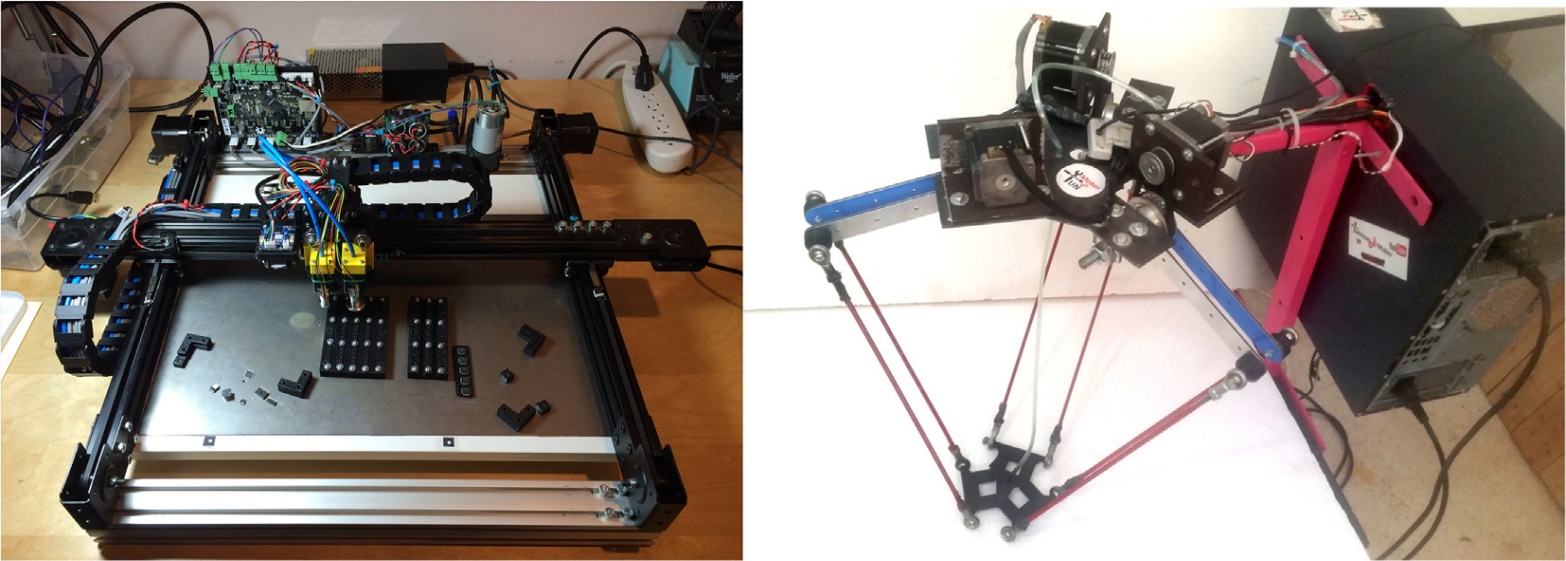
OpenBuilds by OpenPnP [13] (left) and “Delta Robot Open Source Project” by TunMaker [14] (right).

Besides robotic arms, the open-source community has devised several alternate non-robotic arm solutions capable of performing similar functions to PARA (pick-and-place, tool handling, etc.). However, these solutions often rely on end effectors mounted on Cartesian CNC router-style or delta-style frames (Fig. 4). The OpenPnP project has even compiled a list of such open-source Cartesian-style pick-and-place machines, such as its own OpenPnP OpenBuilds machine [12]. While oftentimes relatively cheap and simple in construction, these machines require space-consuming mounting infrastructure like rails and gantries, and cannot maneuver into tight areas like a robotic arm can. The frames required for a traditional Cartesian-style or delta-style pick-and-place machine with an identical workspace to PARA would be enormous, spanning over 2 meters in each dimension.

**Fig. 5:**
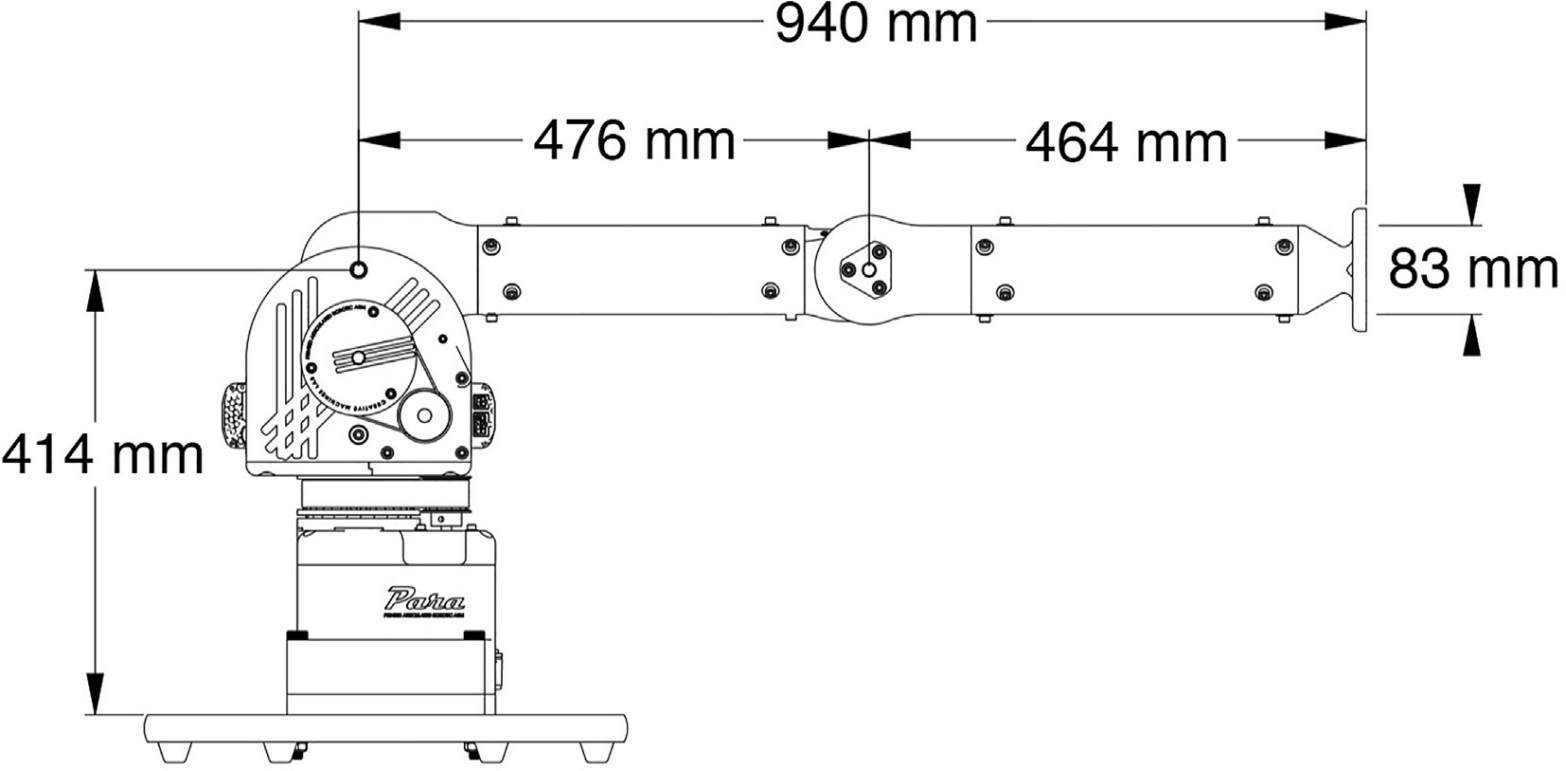
Engineering drawing of PARA with labeled dimensions.

By featuring payload-lifting capabilities just 2–3 times less than industrial-grade robotic arms at a price point cheaper by an order of magnitude while being much larger and more powerful than similarly-priced robotic arms, PARA attempts to distinguish itself in the current market of robotic arms and provide an affordable yet functional alternative. It also seeks to serve as an educational tool for the research community: with its functional capabilities and accessible design files and build instructions, users are encouraged to implement their own features on PARA such as additional degrees of freedom, custom end effector attachments like grippers or tools, or more complex control schemes.

**Fig. 6:**
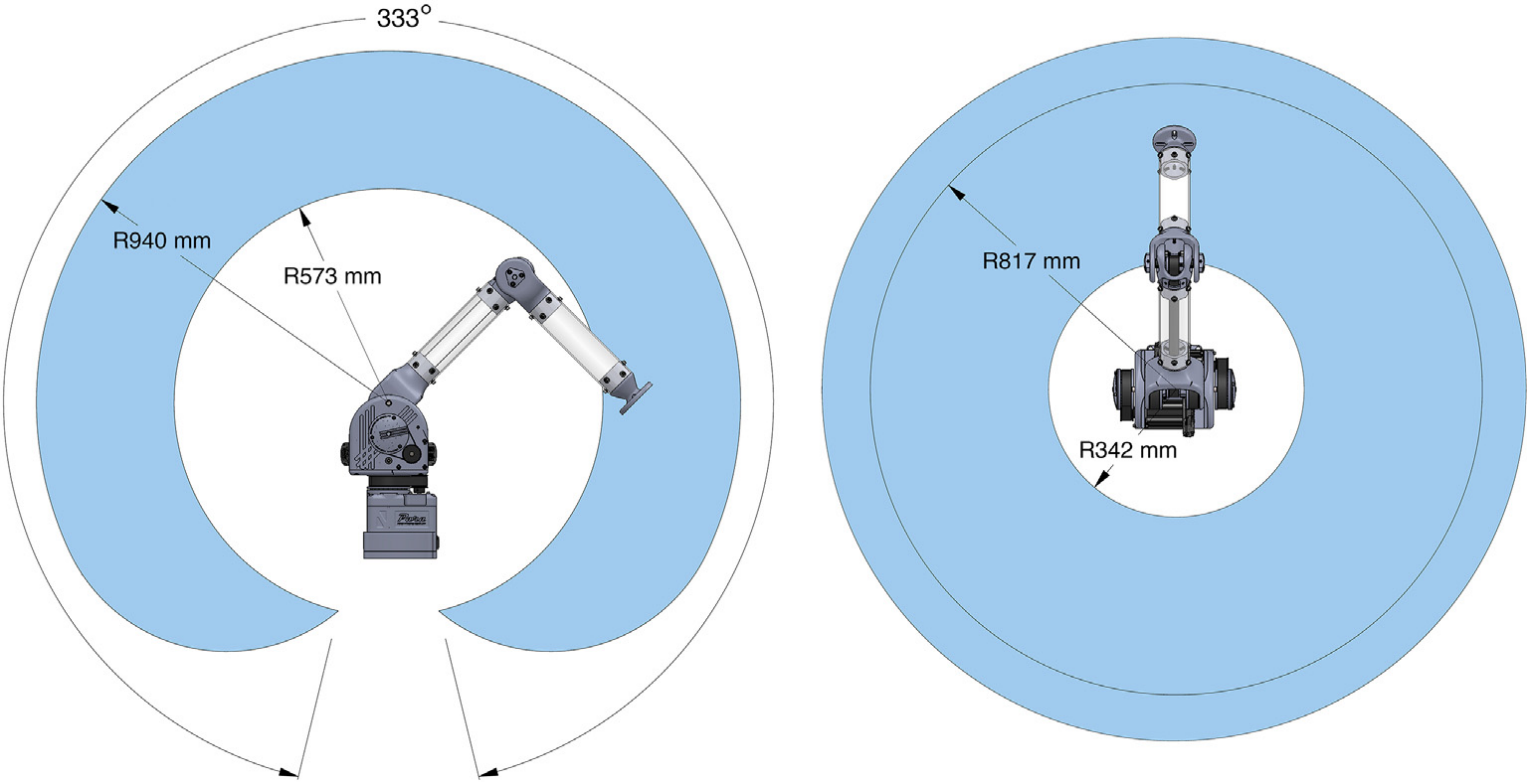
Workspace of PARA with labeled dimensions.

## 2 Hardware description

Technical specifications and dimensions for PARA are as follows:

**Table:** 

Degrees of Freedom	3
Payload	2 kg
Repeatability	± 2.6 mm at 250 mm/s
Reach	940 mm
Cost	Approx. $3400

**Fig. 7:**
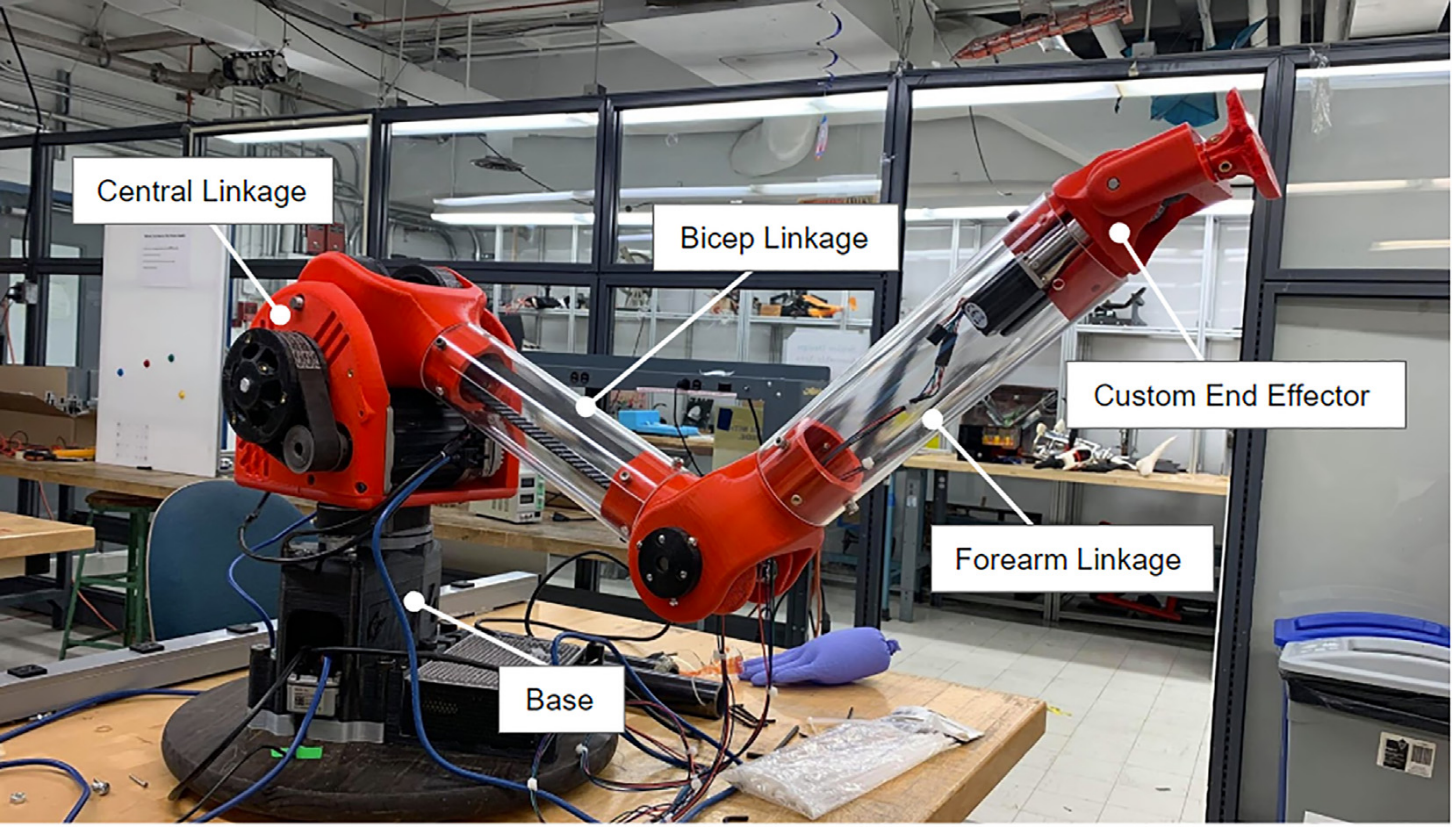
High-Level overview of PARA parts.

Fig. 5 and Fig. 6 showcase PARA's critical dimensions and workspace. PARA features three joints and three linkages, which are labeled in Fig. 7. In this article, the joint between the base and central linkage will be referred to as the base joint, the joint between the central and bicep linkages will be referred to as the shoulder joint, and the joint between the bicep and forearm linkages will be referred to as the elbow joint.

**Fig. 8:**
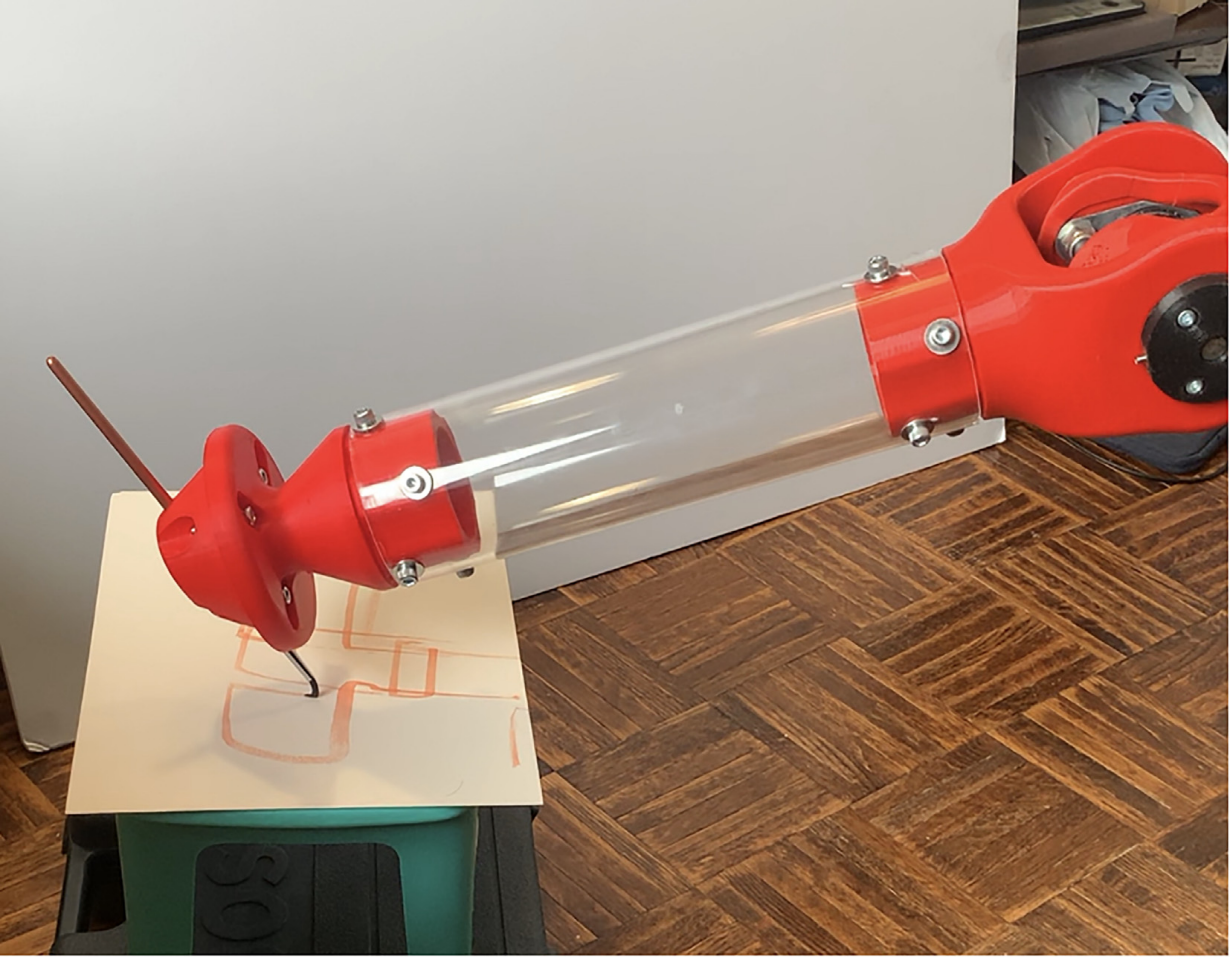
PARA creating a watercolor artwork using a paintbrush mounted on its end effector mounting plate.

The forearm linkage terminates at a mounting plate for the user to add custom actuators with additional degrees of freedom (see Fig. 8).

PARA’s linkages are actuated either by timing belt trains or individual timing belts, which are in turn driven by motors. A NEMA 23 motor actuates the base joint, while two NEMA 34 motors housed in the central linkage actuate the shoulder and elbow joints. The elbow joint motor actuates the forearm linkage completely independently of the rest of the arm, while the shoulder joint motor actuates both the shoulder and elbow joints at the same time (with PARA’s controls accounting for this interdependence by sending commands to the elbow joint motor and offsetting the forearm linkage’s motion while the shoulder joint is actuated, thus preserving the system’s three completely independent degrees of freedom).

The NEMA 23 motor which actuates the base joint is smaller than the NEMA 34 motors because it does not have to act against gravitational forces. The NEMA 34 motors were placed as close to the central axle of the arm as close as possible to minimize the system’s moment of inertia. The motors are powered by a 75 V power source and controlled by G-Code sent from a Smoothieware Smoothieboard 3D printer control board (these electonic components are easily replaceable with alternatives that are cheaper or more familiar to the user). Further detail on how PARA’s motors and power source were selected can be found in Section 2.6.

With a customizable end effector and considerable load-lifting capabilities, PARA can be customized for a variety of different tasks, such as:

• Small-scale automated pick and place operations
• Serving as a testbed for various robotics-related projects, such as novel end effectors or control systems
• Automated manufacturing operations such as drilling or bolt tightening via mounted power tools
• Manual task assistance for those with limited mobility
• Arts and entertainment

PARA was designed in imperial units due to the ubiquitous nature of hardware using that measurement system. Its various hardware and software components will now be described in detail in the following sections.

### 2.1 Linkage construction

**Fig. 9:**
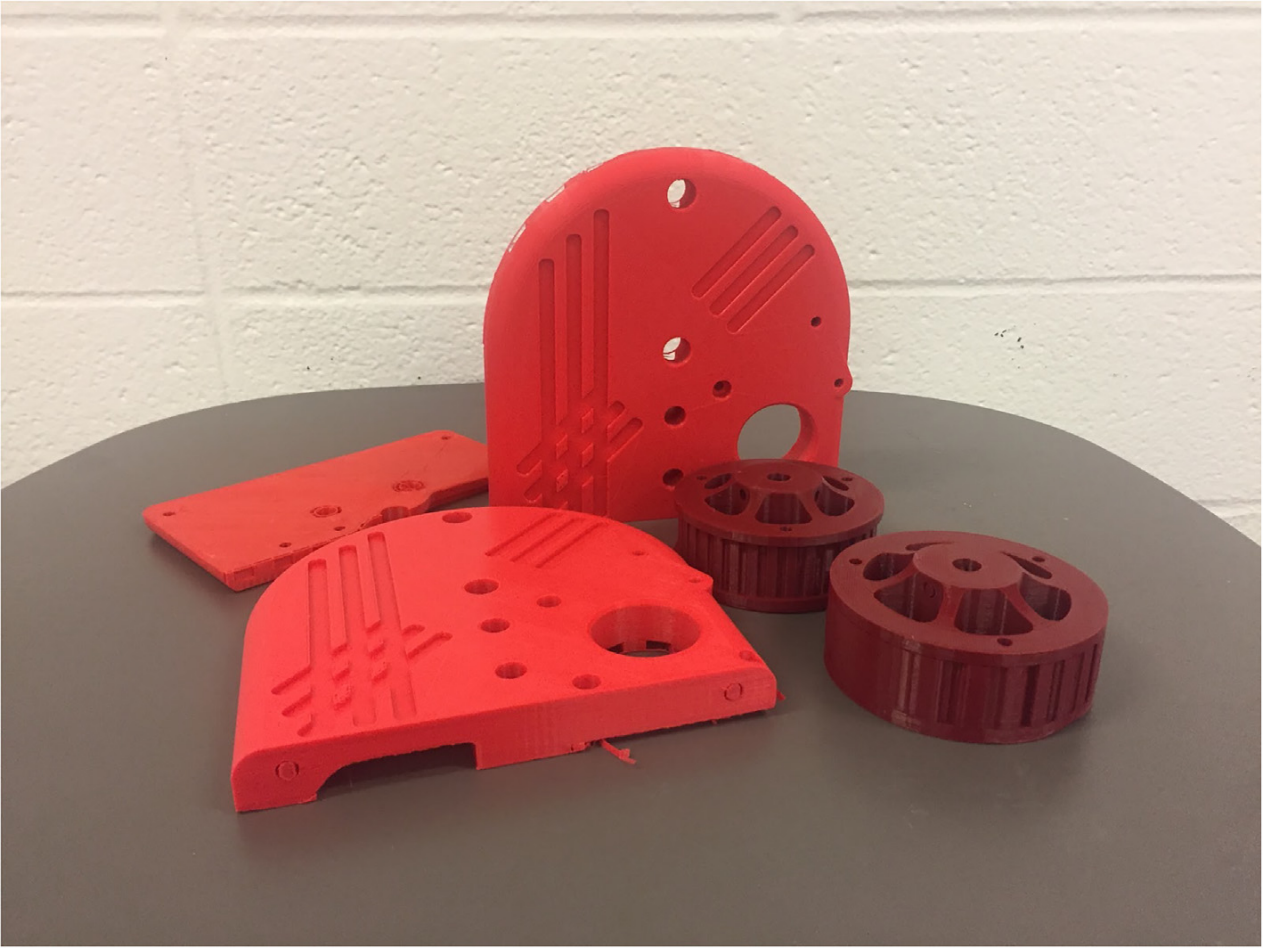
Several printed PARA pieces.

With three degrees of freedom, PARA consists of three linkages: a forearm linkage, a bicep linkage, and a central linkage, which in turn is attached to a base. The central linkage, which primarily consists of printed parts, is by far the largest linkage in terms of print volume. Printing the body of this linkage in a single print process can be risky if left unattended, as the print could fail several hours into the job. Therefore, this central linkage body consists of several smaller pieces with shorter print times (Fig. 9) – if one of these smaller prints fails, the other portions of the linkage would still remain uncompromised. The pieces of the new central linkage are held together by 1/4–20 bolts and heat-set inserts.

The bicep and forearm linkages each consist of two 3D printed end caps fastened to 12” long (304.8 mm) acrylic tubes with a 3.25” (82.6 mm) diameter and 1/8” (3.2 mm) wall thickness. These linkages are fastened to axles by set screws and to timing belt pulleys by bolts.

### 2.2 Linkage actuation system

**Fig. 10:**
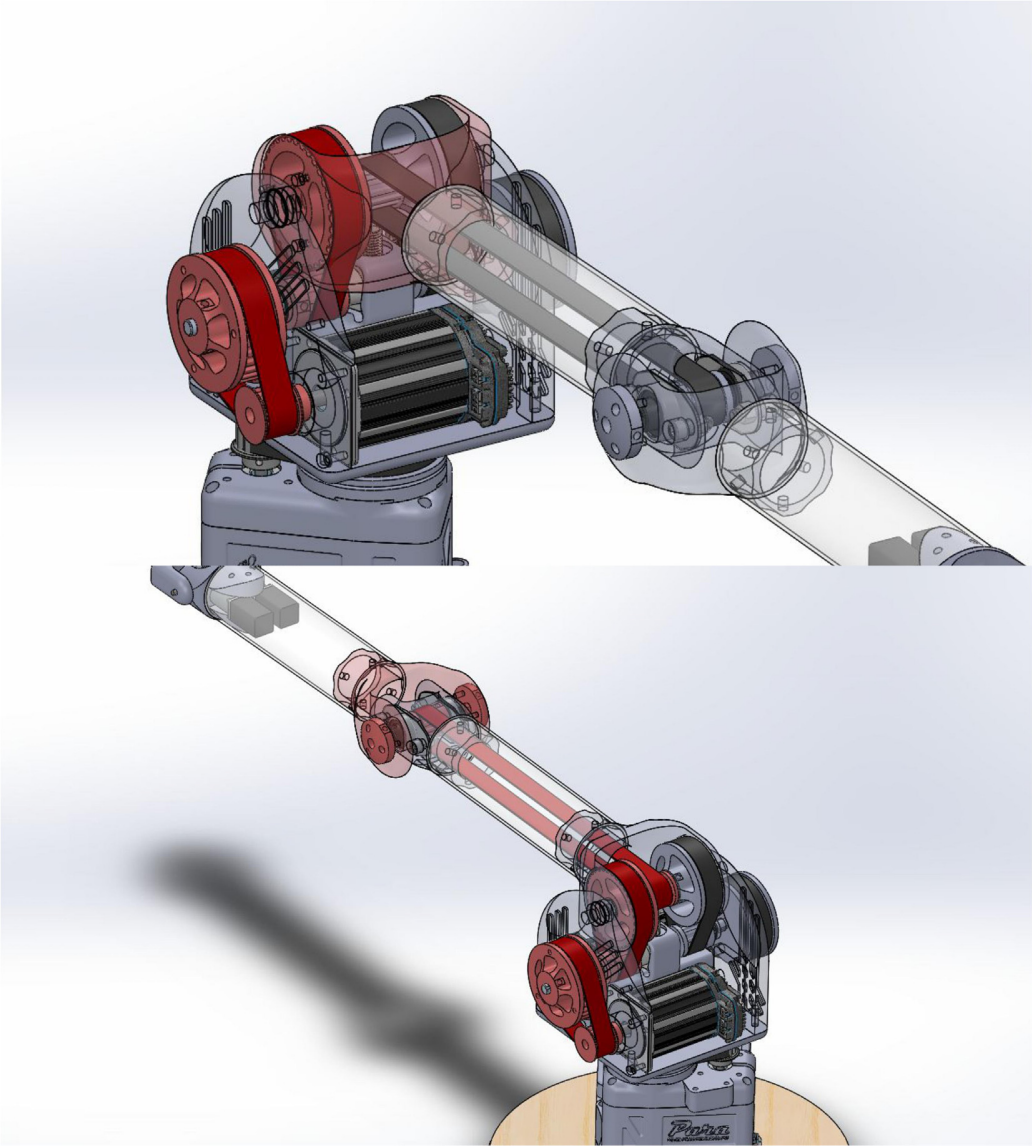
Timing belts involved in actuating shoulder joint (top) and elbow joint (bottom).

PARA’s three degrees of freedom are actuated by a NEMA 23 motor and two NEMA 34 motors, which in turn drive 1” (25.4 mm) wide XL-series timing belt pulleys. The arm features both 3D printed and aluminum pulleys. The pulleys’ thickness ensures robustness through force distribution over a greater cross-sectional area (thus leading to less elastic deformation), while the large teeth characteristic of the XL profile help prevent belt slippage. A relatively large contact area between the system’s timing belts and timing belt pulleys also aid in preventing deformation of printed pulleys. How each linkage is actuated by pulleys and one or more timing belts can be seen in Fig. 10.

Timing belts were chosen in favor of steel cables for two primary reasons. First off, the relatively small contact area of cables and the pulleys that drive them would lead to much greater stress concentrations on the pulleys, which would likely result in cables digging into the plastic pulleys. Additionally, anchoring cables to pulleys would require crimping the ends of those cables with ferrules – an extra manufacturing step not required for timing belts.

To increase load-lifting capabilities, gear ratios between driving and driven timing belt pulleys are maximized to the extent allowed by system packaging: a larger gear ratio would call for a larger driven pulley, which in turn would require greater axle distances between driven and driving gears. For the base joint, this axle distance is limited by the size of the base, which should be able to be printed on an approximately 8” × 8” (200 mm × 200 mm) baseplate. For the shoulder and elbow joints, axle distances are limited by the positions of the massive NEMA 34 motors that actuate those joints: the motors are placed as close to the base joint’s axle as possible to minimize the entire system’s moment of inertia. The base joint features a gear reduction of 1:2.7, while the shoulder and elbow joints feature gear reductions of 1:7.5.

For compact packaging and weight savings, PARA’s power transmission system shares axles between as many of the required timing belt pulleys as possible, though this is not without accompanying complications. From studying Fig. 10, one can observe how moving the bicep linkage will also move the elbow joint axle – and the 42 tooth timing belt that directly actuates forearm linkage – relative to the rest of the elbow joint’s power transmission system. Therefore, actuating the bicep linkage θ degrees will actually also actuate the forearm linkage by -θ. The controls for the arm compensate for this effect by applying power to the elbow joint while the shoulder joint is in motion. For example, if the forearm linkage has to remain a constant angle relative to the bicep linkage while the bicep linkage moves θ degrees, the control board will send commands to the elbow joint motor to also actuate that joint by θ degrees, thus cancelling out the -θ degree motion and resulting in zero net movement of the forearm linkage. Fortunately, this effect is not present the other way around – directly actuating the forearm linkage by itself will not actuate the bicep linkage. Therefore, if both the bicep and forearm linkage now have to move θ degrees, the control board would still actuate the shoulder joint by θ degrees, but now the elbow joint would be actuated by 2θ degrees. Thus PARA still maintains its three independent degrees of freedom.

**Fig. 11:**
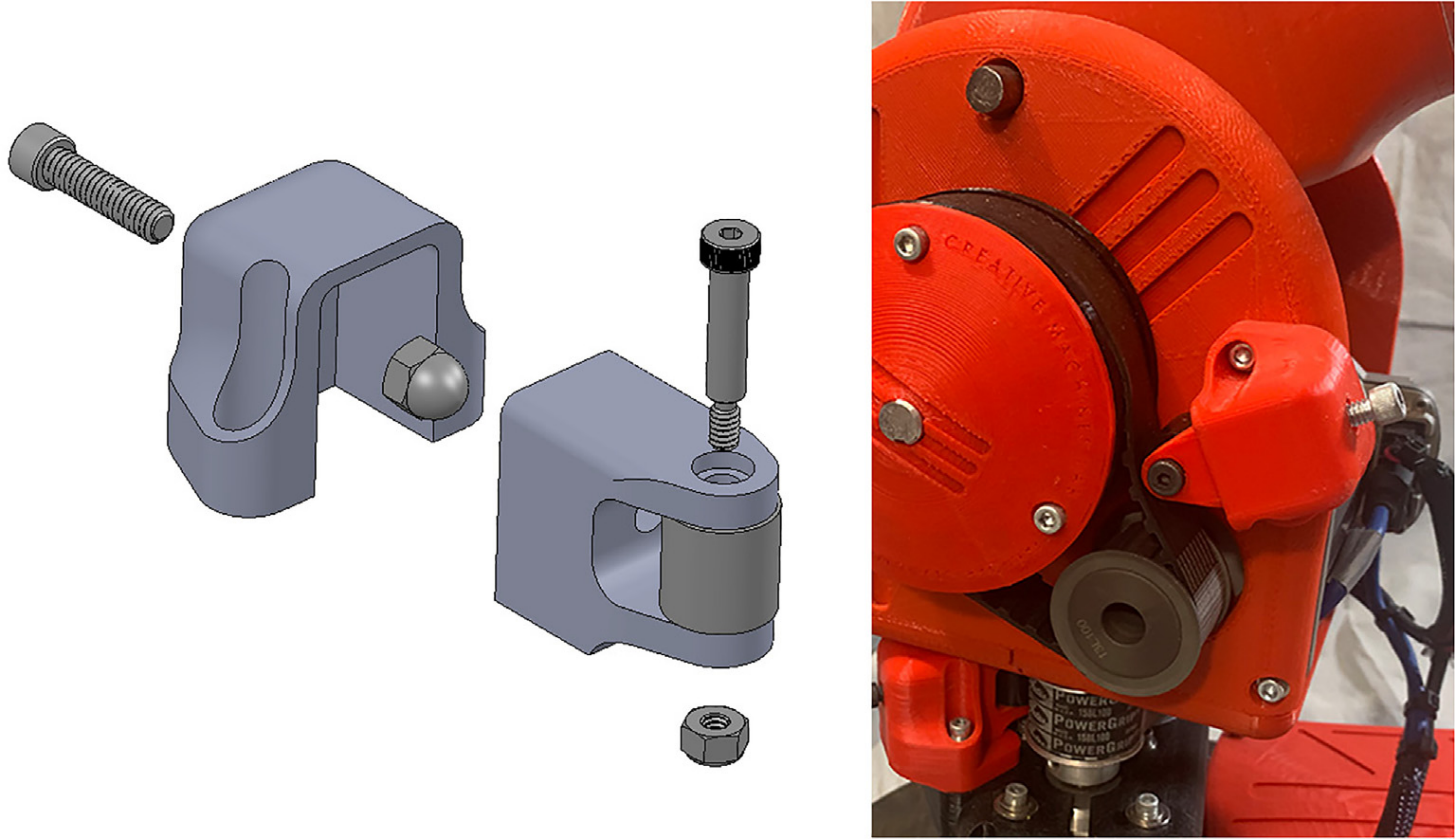
CAD model showing tensioner assembly (left) and a detail view of the implemented tensioner mechanism on the central linkage (right).

A number of idler-based belt tensioners (Fig. 11) can be mounted adjacent to many of the system’s belts should those belts lose tension. These tensioners are loosened and tightened by turning and un-turning a bolt in the tensioner mechanism.

### 2.3 Base joint

**Fig. 12:**
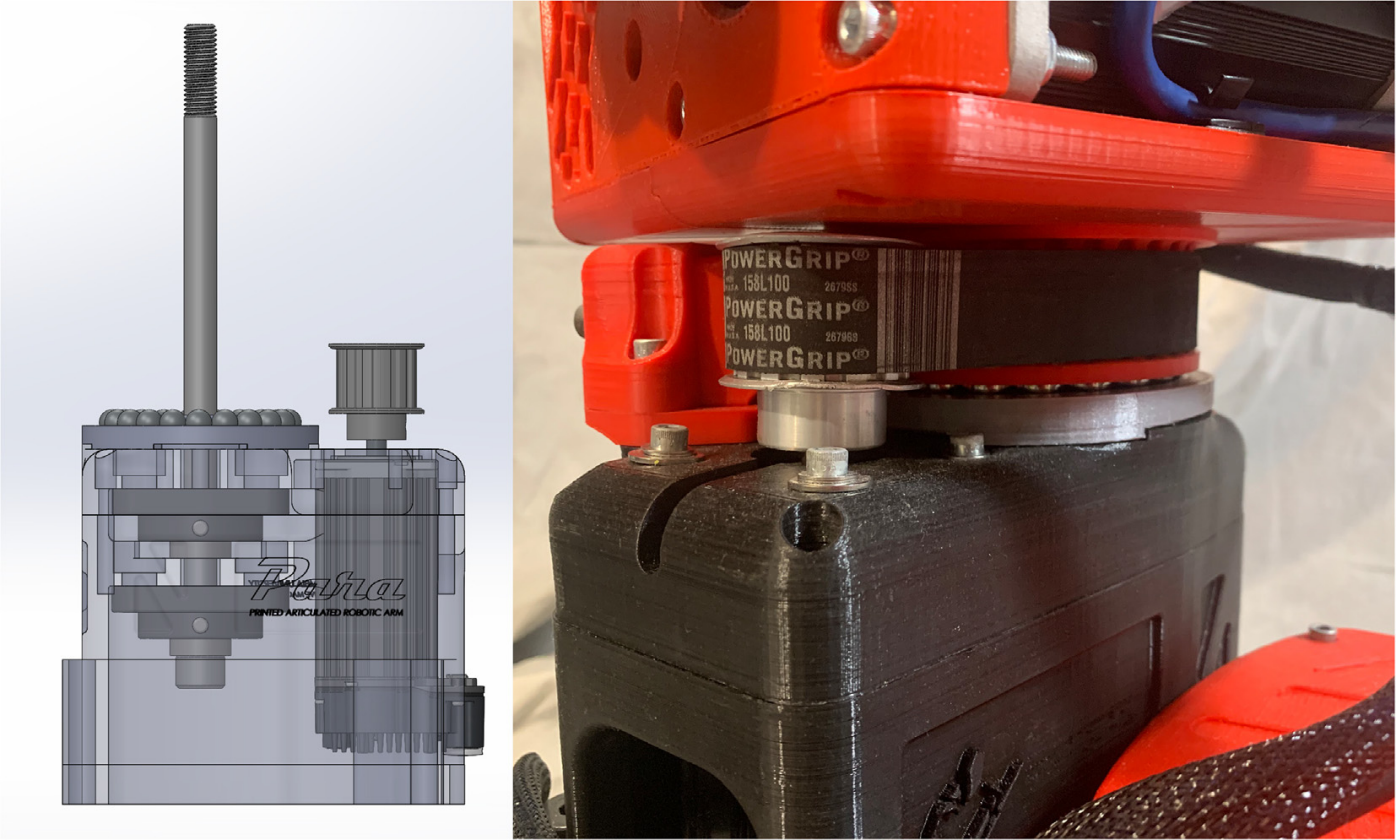
CAD model showing PARA’s central axle assembly (left) and a detail view of the implemented thrust bearing mechanism (right).

The arm’s central linkage is fixed to a 12” (304.8 mm)-long 5/8” (15.9 mm)-diameter bolt which is secured to two mounted bearings housed in the base (Fig. 12).

PARA features a custom thrust ball bearing-like system which enables motion between the central linkage and the base. 21.5” (12.7 mm) ball bearing balls are placed in a circular track about 1.68” (42.7 mm) away from the central axle (Fig. 12). Two large steel washers sandwich the steel balls, thus all the load carried by the bearings is reacted by the washers rather than bare PLA plastic (which can deform due to the stress concentrations at the contact patch of the balls). 3D printed pieces guide the steel balls to ensure that they stay in their proper positions, however the majority of the load on these balls (vertical gravitational force) is still being reacted by the washers.

These steel balls aid in supporting the central linkage by reacting gravitational forces exerted on the bicep and forearm linkages at a radius relatively far away from the central axle. Actual ball or needle thrust bearings were not used because thrust bearings of a similar diameter are both rare and costly.

### 2.4 Shoulder and elbow joints

**Fig. 13:**
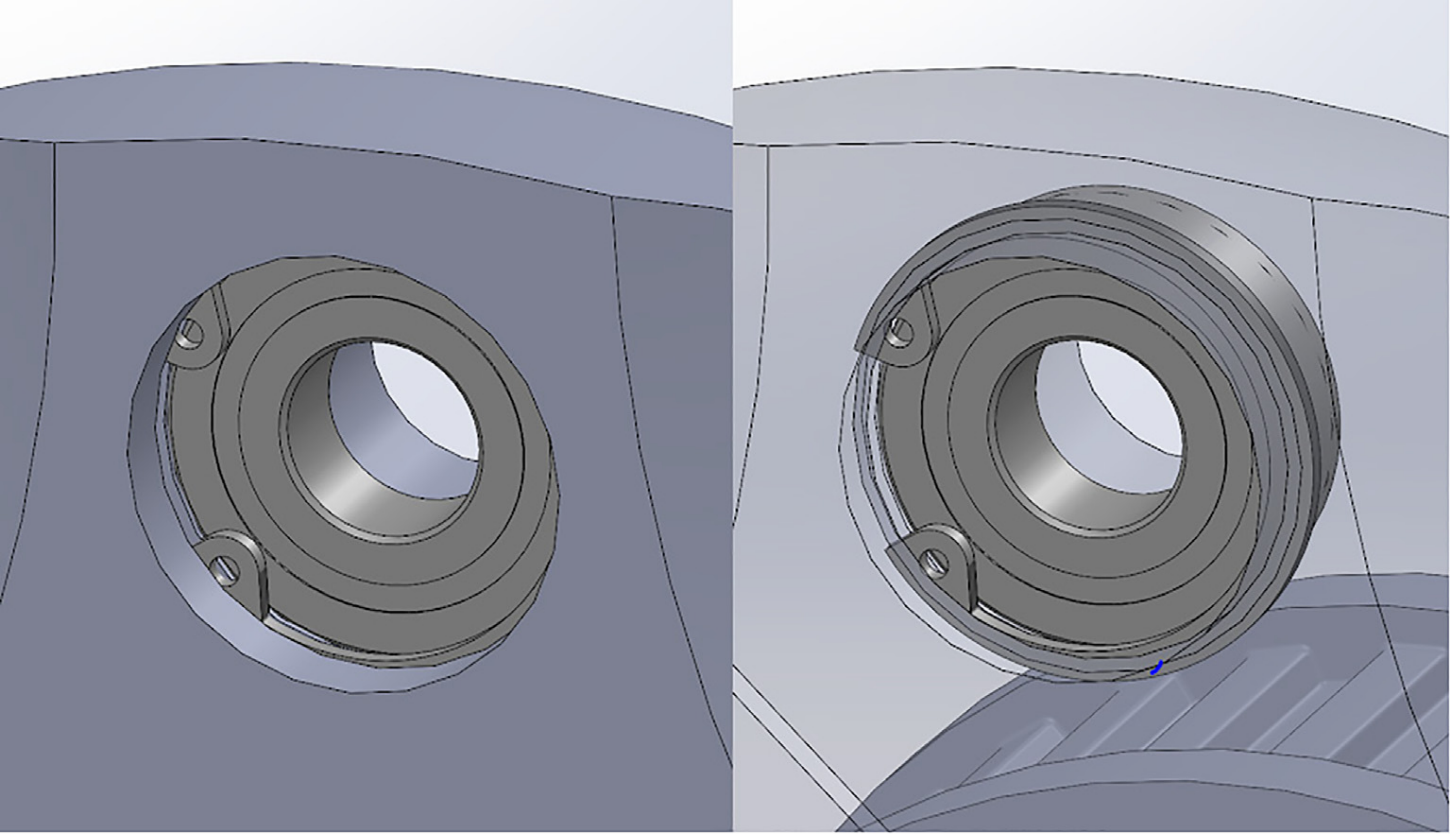
Detail view of PARA’s press-fit ball bearings with retaining rings.

PARA’s shoulder joint consists of a 9” (228.6 mm) steel axle which passes through the bicep and central linkages. The bicep link is constrained to the axle via #10–32 thread set screws, while motion between the two linkages is enabled by ball bearings press-fit into the central linkage. Retaining rings hold the bearings in place (Fig. 13). Roller thrust bearings between the bicep linkage and timing belt pulley actuating the forearm linkage reduce friction between those parts.

The elbow joint consists of a 6” (152.4 mm) aluminum axle which passes through the bicep and forearm linkages. #10–32 set screws constrain the forearm linkage to the axle, while the bicep linkage is constrained to the axle via mounted bearings with embedded set screws.

### 2.5 Mounting plate

**Fig. 14:**
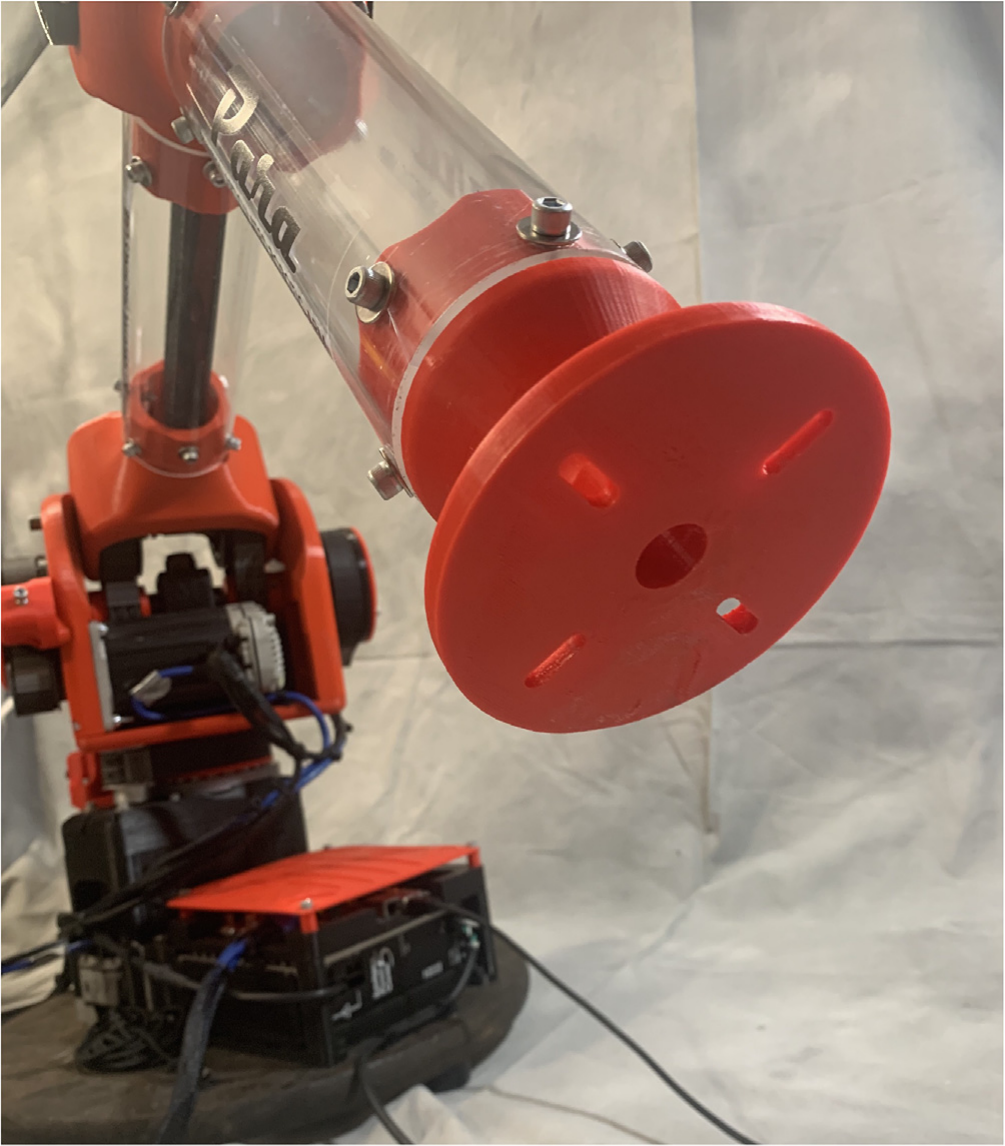
Detail view of PARA’s end effector mounting plate.

The end of the forearm linkage terminates in a mounting plate (Fig. 14) so that custom end effectors providing additional degrees of freedom can attach to PARA.

### 2.6 Motor selection and electronics hardware

**Fig. 15:**
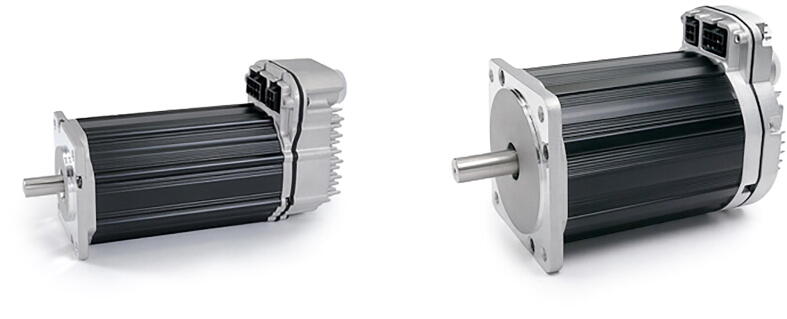
Teknic Clearpath servo motors used in PARA: NEMA 23 CPM-SCHP-2341P-ELNB [15] and NEMA 34 CPM-SCHP-3441S-ELSB [16].

Although any NEMA 23 and 34 stepper motors should have the proper shaft and bolt mounting geometry to be compatible with PARA (albeit load-handling capabilities will be affected by using motors of differing holding torques), the version of PARA built by the Creative Machines Lab features Teknic Clearpath servo motors (CPM-SCHP-2341P-ELNB [1] with a max holding torque of 2.9 Nm for the base joint and CPM-SCHP-3441S-ELSB with a max holding torque of 13.0 Nm for the shoulder and elbow joints; see Fig. 15). While expensive, these motors were useful for prototyping due to their various built-in safety features, such as shutdown protocols for excessive current draw and operating temperature.

PARA’s motors were first selected via some simplified back-of-the-envelope calculations, assuming a desired capability to lift 1 kg of load at a 1 m reach (though after design iteration and empirical evaluation it surpassed this goal and proved capable of supporting 2 kg). Once the development of the robot progressed, these calculations were then further verified through more in-depth analysis incorporating the rest of the robot’s design.

Preliminary calculations assumed two 3 kg motors (the approximate mass of most NEMA 23 and 34 models) housed 100 mm away from a central axle. The length of the arm’s bicep (shoulder joint to elbow joint) and forearm linkages (elbow joint to end effector) were both assumed to be 500 mm. The mass of the rest of the robot (which would primarily consist of 3D printed and acrylic parts) was assumed to be negligible for now.

**Fig. 16:**
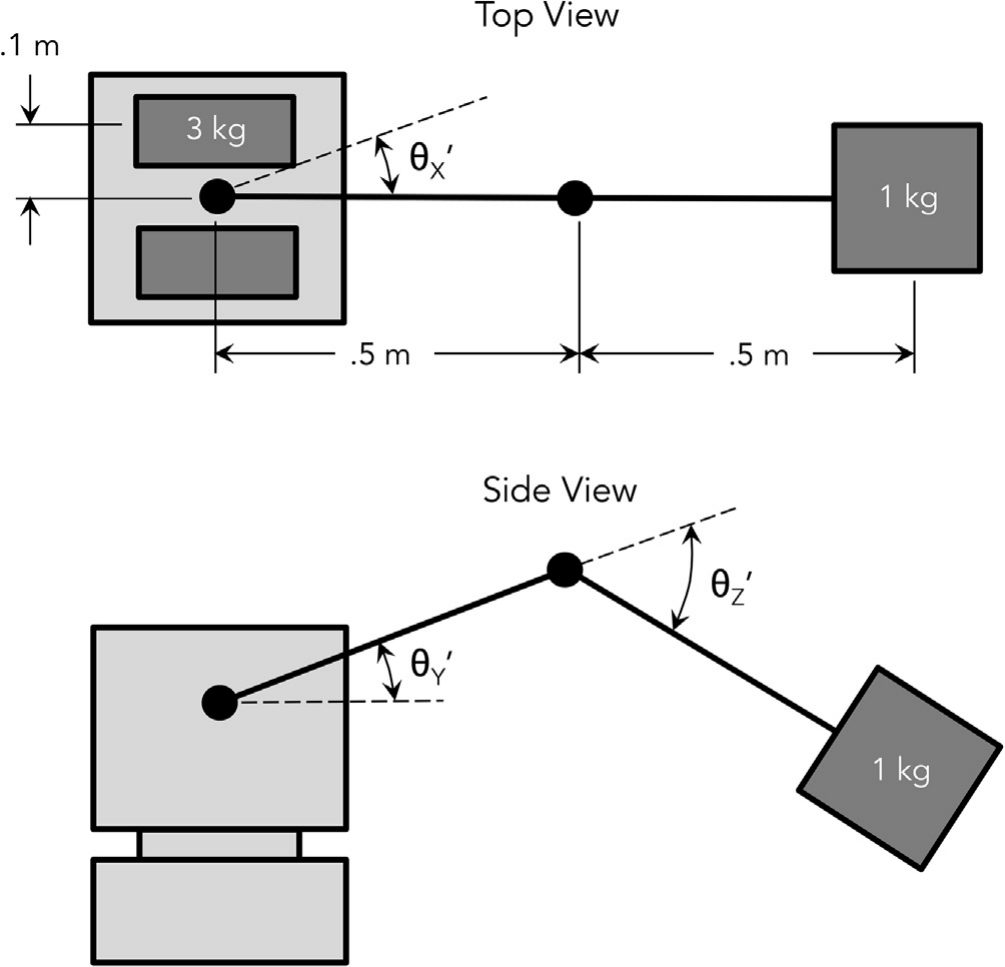
Initial model for PARA linkage mass properties.

Fig. 16 details this rudimentary model. The joints have been denoted with a “prime” symbol. The moment of inertia about the elbow ($\theta_{Z}^{\prime }$), shoulder ($\theta_{Y}^{\prime }$), and base ($\theta_{X}^{\prime }$) joints (accounting for the payload) can be calculated as follows:

(1) $$I_{\theta Z^{\prime }}=1\;\mathrm{kg \cdot}{\left(0.5\;m\right)}^{2}=0.25\;\mathrm{kg}\;m^{2}$$

(2) $$I_{\theta Y^{\prime }}=1\;\mathrm{kg}\;\cdot \;{\left(1\;m\right)}^{2}=1\;\mathrm{kg}\;m^{2}$$

(3) $$I_{\theta X^{\prime }}=2\;\cdot \;3\;\mathrm{kg \cdot}{\left(0.1\;m\right)}^{2}+1\;\mathrm{kg \cdot}{\left(1\;m\right)}^{2}=1.06\;\mathrm{kg}\;m^{2}$$

To approximate the arm’s desired angular accelerations, a desired end effector velocity of 250–1000 mm/s in each translational direction was established. Given the estimated robot dimensions, this range of end effector velocities would resolve to desired joint velocities of 0.25–1.00 rad/s (2.4–9.5 rpm) for $\theta_{X}^{\prime }$ and $\theta_{Y}^{\prime }$, and 0.5–2.00 rad/s (4.8–19.0 rpm) for $\theta_{Z}^{\prime }$.

**Fig. 17:**
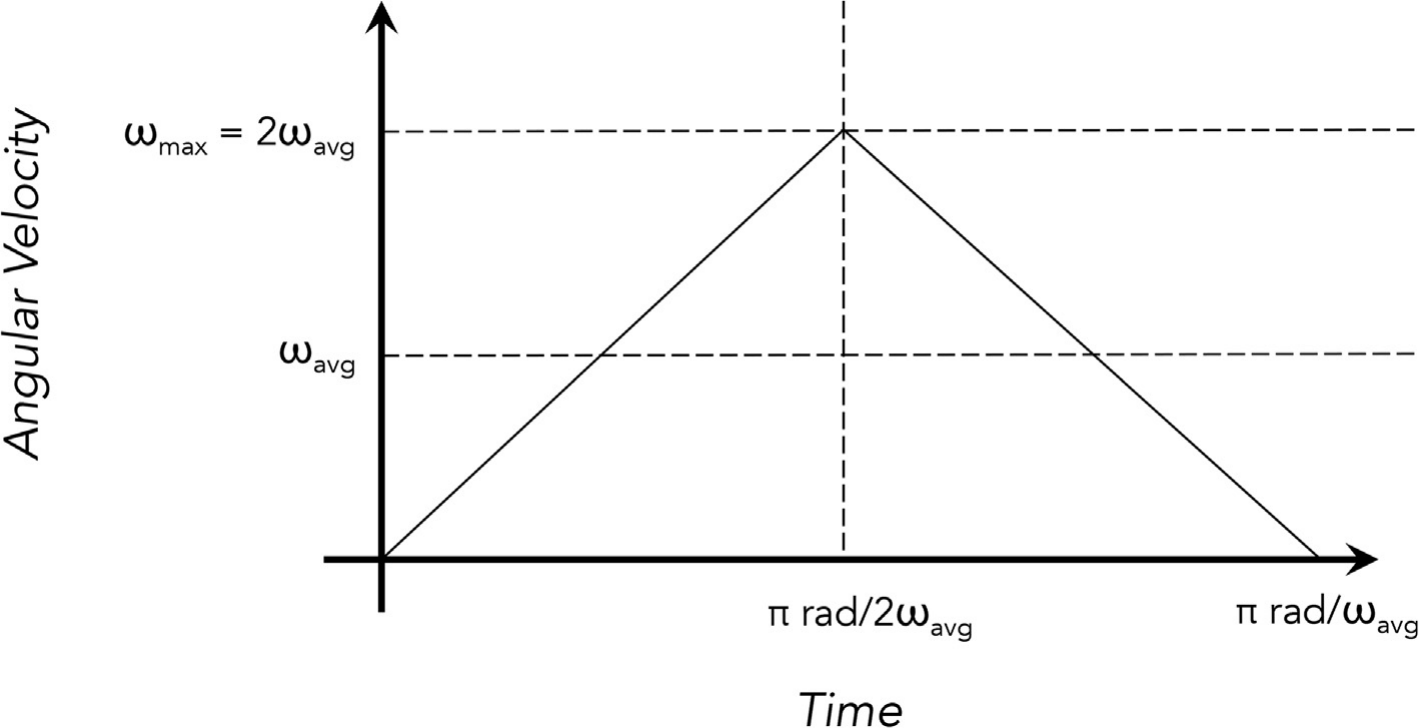
Triangular angular velocity profile with an average velocity of $\omega_{\mathrm{avg}}$.

Assuming extremely rudimentary triangular velocity profiles for each joint across an angular sweep of π radians (Fig. 17):

The desired angular acceleration for each joint would then equal $4/\pi *\omega_{\mathrm{avg}}^{2}$. These values equal 0.08–1.27 rad/s^2^ for $\theta_{X}^{\prime }$ and $\theta_{Y}^{\prime }$, and 0.32–5.09 rad/s^2^ for $\theta_{Z}^{\prime }$.

Using the simple relationship τ=Ia, the required torque ranges to accelerate the various joints to the desired values (neglecting the effects of gravity) would equal 0.08–1.34 Nm for $\theta_{X}^{\prime }$, 0.08–1.27 Nm for $\theta_{Y}^{\prime }$, and 0.08–1.27 Nm for $\theta_{Z}^{\prime }$. Meanwhile, the torques required to counteract gravitational force at the worst case scenario would equal 4.91 Nm (0.5 m ⋅ 1 kg ⋅ 9.81 m/s^2^) at $\theta_{Z}^{\prime }$ and 9.81 Nm (1 m ⋅ 1 kg ⋅ 9.81 m/s^2^) at $\theta_{Y}^{\prime }$. Combining these values yields maximum desired torque values of 1.34 Nm for $\theta_{X}^{\prime }$, 11.08 Nm at $\theta_{Y}^{\prime }$, and 6.18 Nm at $\theta_{Z}^{\prime }$.

A NEMA 23 motor with a maximum torque of 2.9 Nm was chosen to actuate $\theta_{X}^{\prime }$, while a NEMA 34 motor with a maximum torque of 13.0 Nm was chosen to actuate $\theta_{Z}^{\prime }$ (for a factor of safety of about 2 for both motors). The same NEMA 34 motor was also chosen for $\theta_{Y}^{\prime }$. Though the maximum desired torque (11.08 Nm) was close to the maximum operating torque of the NEMA 34 motor (13.0 Nm), any larger motor would’ve been impractical in terms of mass and cost. A gear ratio would have to be applied to increase the motor’s torque output.

**Fig. 18:**
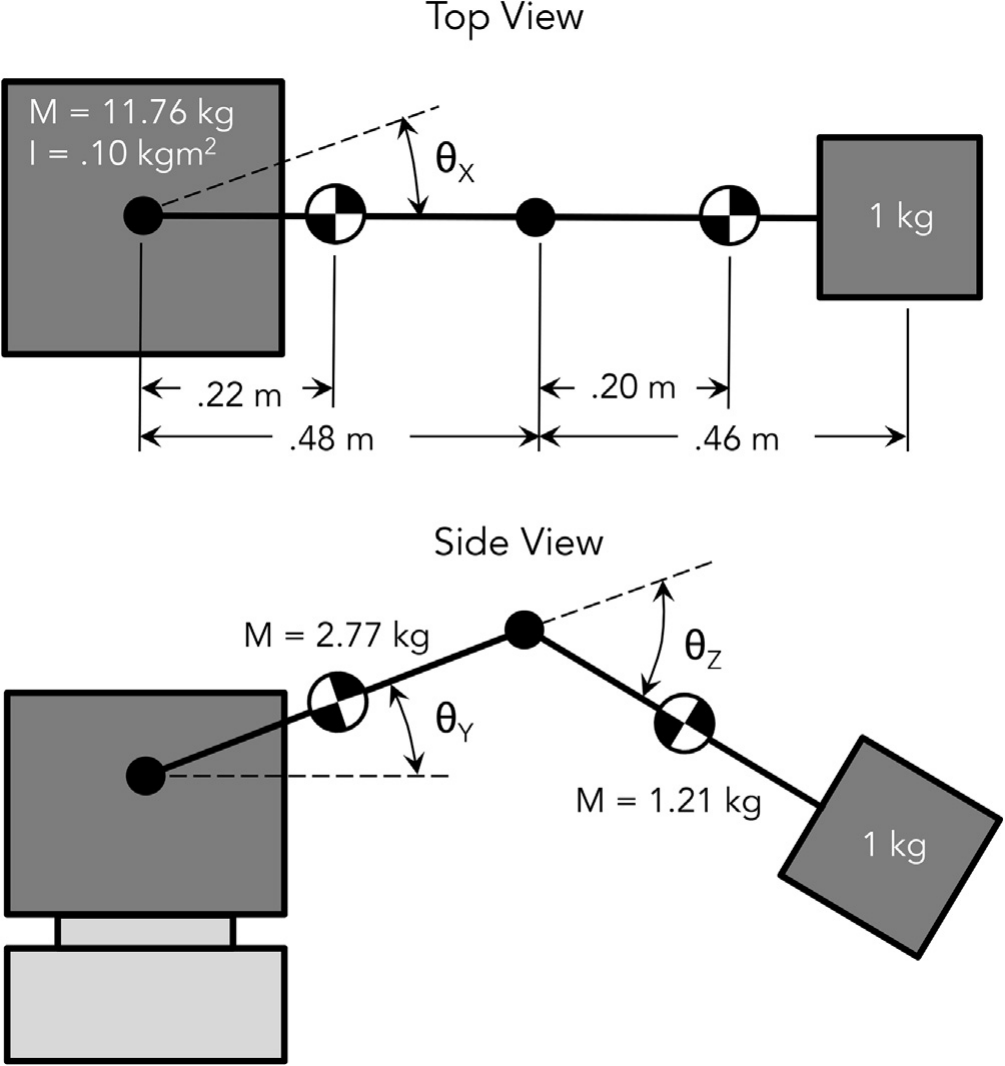
Refined model for PARA linkage mass properties informed by CAD data.

After more practical design work was conducted on the arm, the efficacy of the selected motors was once again evaluated. Applying mass properties to various linkages of the arm in CAD yielded the mass and moment of inertia seen in Fig. 18:

The moment of inertia about the elbow ($\theta_{Z}$), shoulder ($\theta_{Y}$), and base ($\theta_{X}$) joints for this practical model could then be calculated as follows:

(4) $$I_{\theta Z}=1.21\;\mathrm{kg \cdot}\;{\left(0.20\;m\right)}^{2}+1\;\mathrm{kg \cdot}\;{\left(0.46\;m\right)}^{2}=0.26\;\mathrm{kg}\;m^{2}$$

(5) $$I_{\theta Y}=2.77\;\mathrm{kg \cdot}{\;(0.22\;m)}^{2}+1.21\;\mathrm{kg \cdot}\;{\left(0.48\;m+0.20\;m\right)}^{2}+1\;\mathrm{kg \cdot}\;{\left(0.48\;m+0.46\;m\right)}^{2}=1.58\;\mathrm{kg}\;m^{2}$$

(6) $$I_{\theta X}=0.10\;\mathrm{kg}\;m^{2}+2.77\;\mathrm{kg \cdot}{\left(0.22\;m\right)}^{2}+1.21\;\mathrm{kg \cdot}\;{\left(0.48\;m+0.20\;m\right)}^{2}+1\;\mathrm{kg \cdot}\;{\left(0.48\;m+0.46\;m\right)}^{2}=1.68\;\mathrm{kg}\;m^{2}$$

Using these actual moment of inertia values and the previously-calculated desired angular acceleration values, the new desired torque ranges would equal 0.13–2.13 Nm for $\theta_{X}$, 0.13–2.00 Nm for $\theta_{Y}$, and 0.08–1.32 Nm for $\theta_{Z}$. Once again combining these values with worst-case gravitational forces yields maximum desired torque values of 2.13 Nm for $\theta_{X}$, 11.81 Nm at $\theta_{Y}$, and 6.23 Nm at $\theta_{Z}$.

Ultimately, end effector speed was sacrificed in favor of payload capacity for PARA, and the design sought to maximize the gear ratio of each joint within allowable packaging. As previously stated, $\theta_{X}$ features a gear reduction of 1:2.7, while $\theta_{Y}$ and $\theta_{Z}$ feature gear reductions of 1:7.5. Therefore, for the initial 1 kg goal, the required output torque for the base joint would be 0.79 Nm, while the required output torques for the shoulder and elbow joints respectively would equal 1.57 Nm and 0.83 Nm. These values fall comfortably into the operating torque ranges for the selected motors – in fact, working through these calculations backwards and solving for the max load which can actuated by each joint with the provided motor torque capabilities yields theoretical maximum payloads of 6.1 kg for the base joint, 8.8 kg for the shoulder joint, and 16.3 kg for the elbow joint. Actual empirical testing, however, demonstrated that these axes could only operate payloads of about 2 kg before encountering other sources of mechanical failure such as belt slippage. Still, this value is greater than the designed payload of 1 kg.

PARA's required power capacity was determined by multiplying by the worst-case motor torques by worst-case angular velocities. The arm’s motors are daisy-chained together and powered by a Teknic IPC-5 75 V power supply [3]. Performing the previous calculations once more with a payload of 2 kg yields worst-case motor torques of 1.20 Nm for $\theta_{X}$, 3.03 Nm for $\theta_{Y}$, and 1.63 Nm for $\theta_{Z}$. The maximum joint angular velocities in these events were previously calculated as 1.27 rad/s^2^ for $\theta_{X}^{\prime }/\theta_{X}$ and $\theta_{Y}^{\prime }/\theta_{Y}$, and 5.09 rad/s^2^ for $\theta_{Z}^{\prime }/\theta_{Z}$. Taking gear ratios into account, the angular velocities at the motors would equal 3.43 rad/s for $\theta_{X}$, 9.53 rad/s for $\theta_{Y}$, and 38.18 rad/s for $\theta_{Z}$. Since P=τω, the maximum power required for each joint can be assumed to be 4.12 W for $\theta_{X}$, 28.88 W for $\theta_{Y}$, and 62.23 W for $\theta_{Z}$ for a combined theoretical max power usage of 95.23 W. Thus, the 75VDC 300 W IPC-5 continuous power supply was chosen to satisfy this power requirement with a reasonable factor of safety of about 3.

**Fig. 19:**
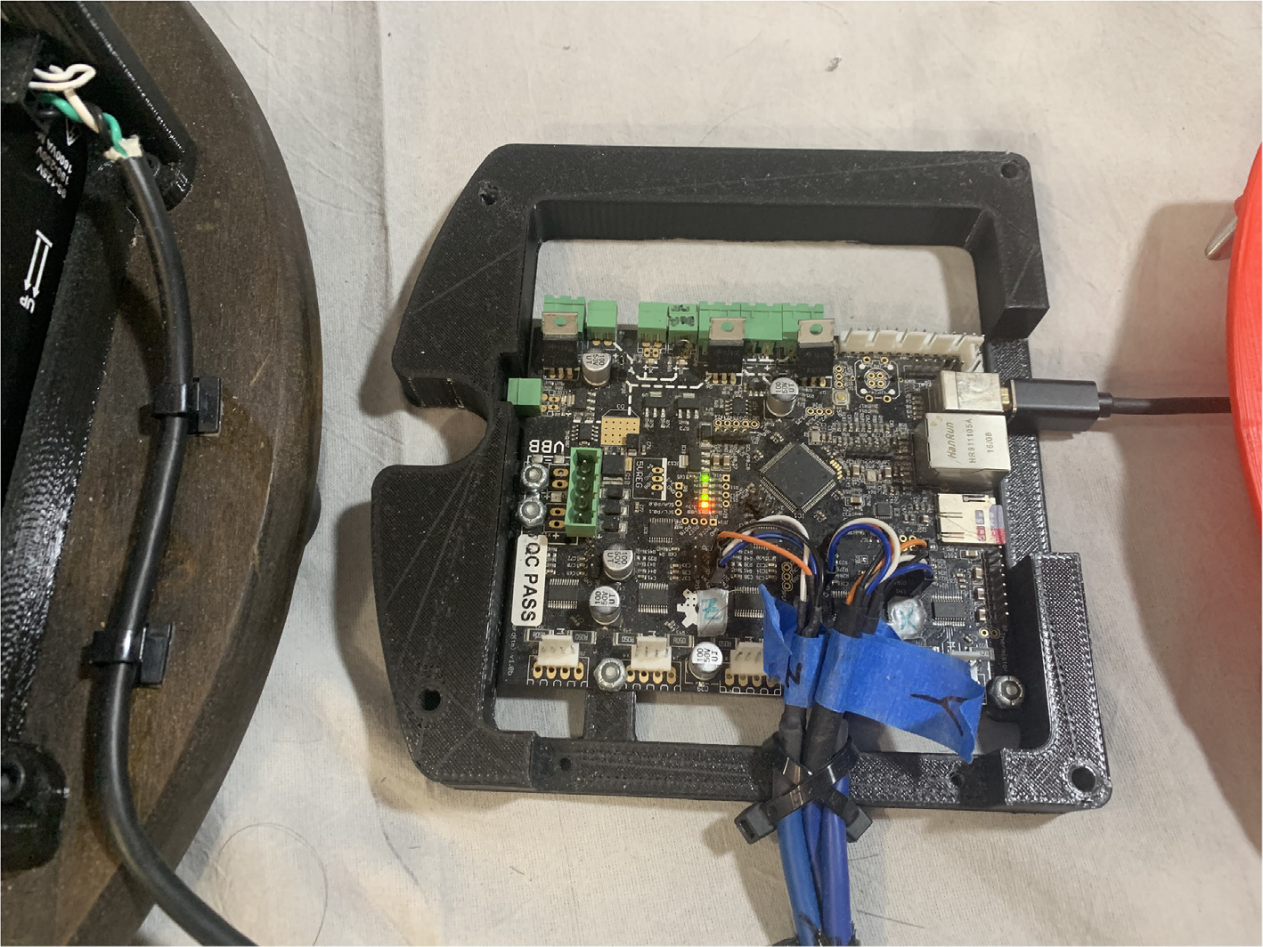
Smoothieboard used to control PARA.

The motors are controlled by a Smoothieboard control board [4] (Fig. 19). Intended for controlling 3D printers, the Smoothieboard was chosen for its on-board motor drivers and ease with interpreting commands. The Smoothieboard can support controlling up to six stepper motors at once for adding additional degrees of freedom to the arm, and its firmware can be easily modified to set motor parameters such as step size. Various free-to-download pieces of software, such as Pronterface from the Printrun suite, can be used to send G-Code commands to the Smoothieboard to control the motors.

Although alternatives for the Teknic motors and the Smoothieboard can be used, this article will focus on supporting these pieces of hardware.

**Fig. 20:**
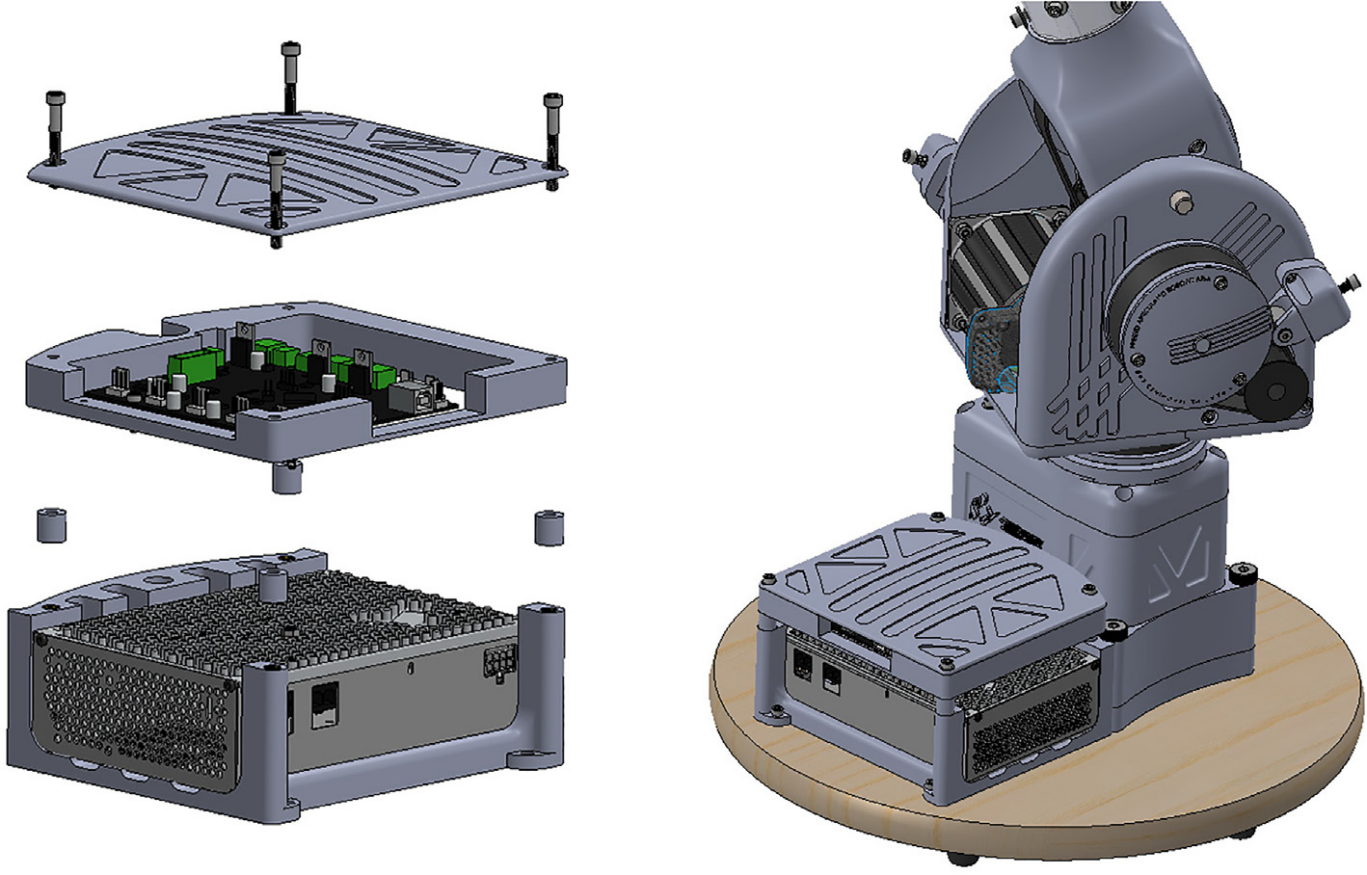
Various components of PARA electronics tower assembly (top-to-bottom): lid, control board, and power source.

An additional three-tiered electronics housing (Fig. 20) can also be printed and installed on PARA’s base to house the arm’s Smoothieboard controller and 75 V power supply. This housing also serves as a brace for the various cables linked to the Smoothieboard, which can be zip tied to the housing for strain protection.

### 2.7 Controls and software

As with most robots and robotic arms, the motor commands required to move PARA can be computed in several different ways.

**Fig. 21:**
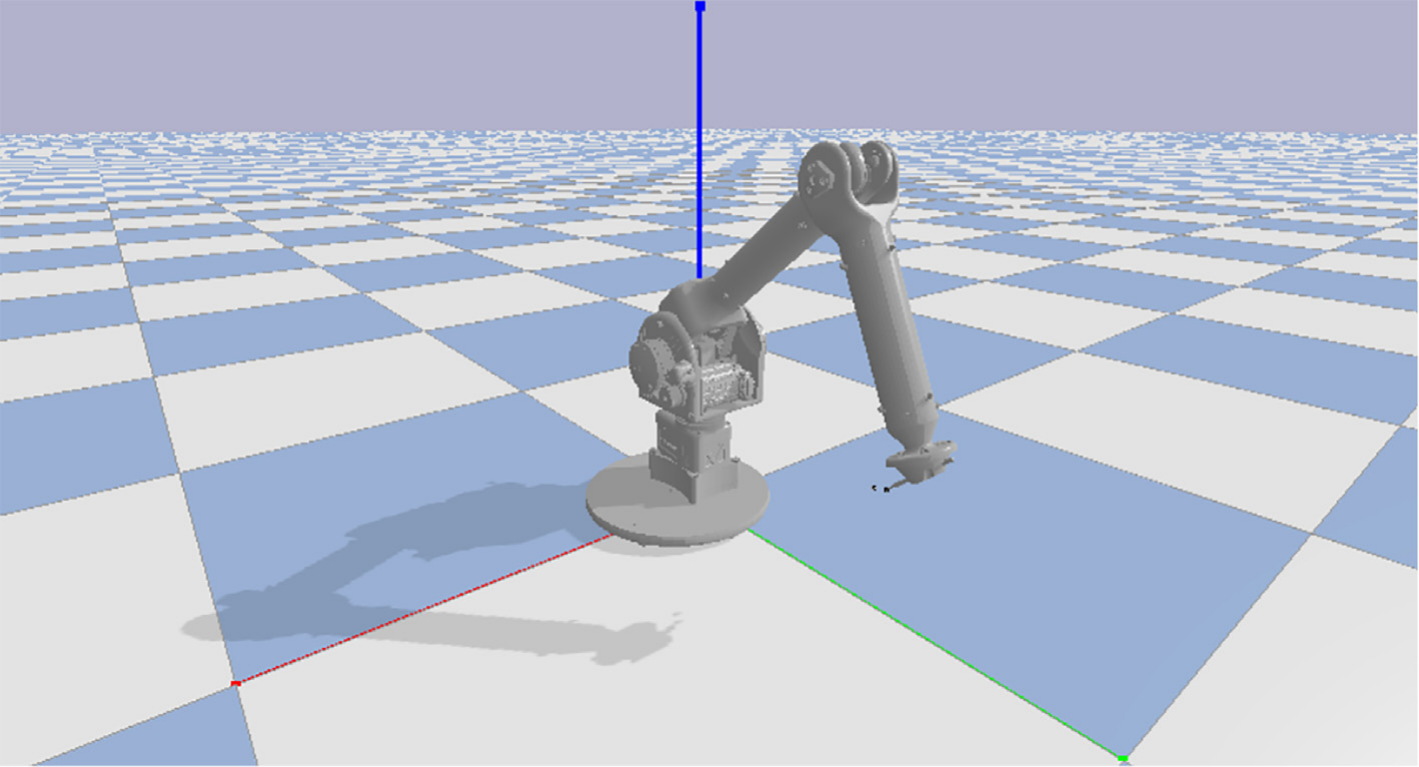
PARA URDF file loaded in PyBullet kinematics engine.

This article presents two. The first method involves loading a URDF file for PARA in a software package with kinetic-simulating capabilities such as Pybullet or ROS (Fig. 21). For those who wish to write their own kinematics engine, the second involves directly using matrix operations to manually compute forward and inverse kinematics.

These methods allow for translating Cartesian commands into desired angles and angular velocities for each motor. As the PARA project was primarily a study in hardware, the acceleration profiles for each joint were not extensively developed, and controls for PARA remain a topic of further study. Clearpath’s proprietary software was used to empirically “tune” each motor based on encountered loads and develop closed-loop control schema for each individual motor. However, much future work can still be performed in this area: rotary encoders and accelerometers mounted in various locations on the arm could allow for the development of more sophisticated PID or PIV control laws for PARA. The mass and moment of inertia information included with PARA’s CAD model could also be used to implement gravity compensation in PARA’s controls.

#### 2.7.1 URDF

As PARA is an articulated robotic arm with three degrees of freedom (DOF), there are three links: the central link, bicep link, and forearm link, and three revolute joints to connect them: the base joint, shoulder joint, and elbow joint. These links and joints are defined in a provided URDF file.

In the URDF file, the kinematic information is encapsulated in the “joint” tags. The “origin” of joint tells where this joint is placed relative to the previous coordinate system.

To aid in visual representation of the arm’s movements, also provided are.STL files representing the various robot links referenced by the URDF file.

Also provided is a sample Python script which uses the PyBullet library in order to translate Cartesian directions for end effector movement into motor angles. The script also translates these angles into GCode commands for the Teknic servos used in this article and sends these commands to the Smoothieboard.

#### 2.7.2 Forward kinematics

Forward Kinematics solves the following question: given all the joint values in the kinematic chain, what is the transform from the base of the chain to each link?

Due to the simplicity of the 3-DOF PARA, the Cartesian position of the end effector frame as functions of joint angles can be computed using just basic trigonometry. $L_{A},L_{B}$, and $L_{G}$ denote the length of the Central Link, Bicep Link, and Forearm Link. The base joint angle, shoulder joint angle, and elbow joint angle have been represented as $\left(\theta_{X},\theta_{Y},\theta_{Z}\right)$.

**Fig. 22:**
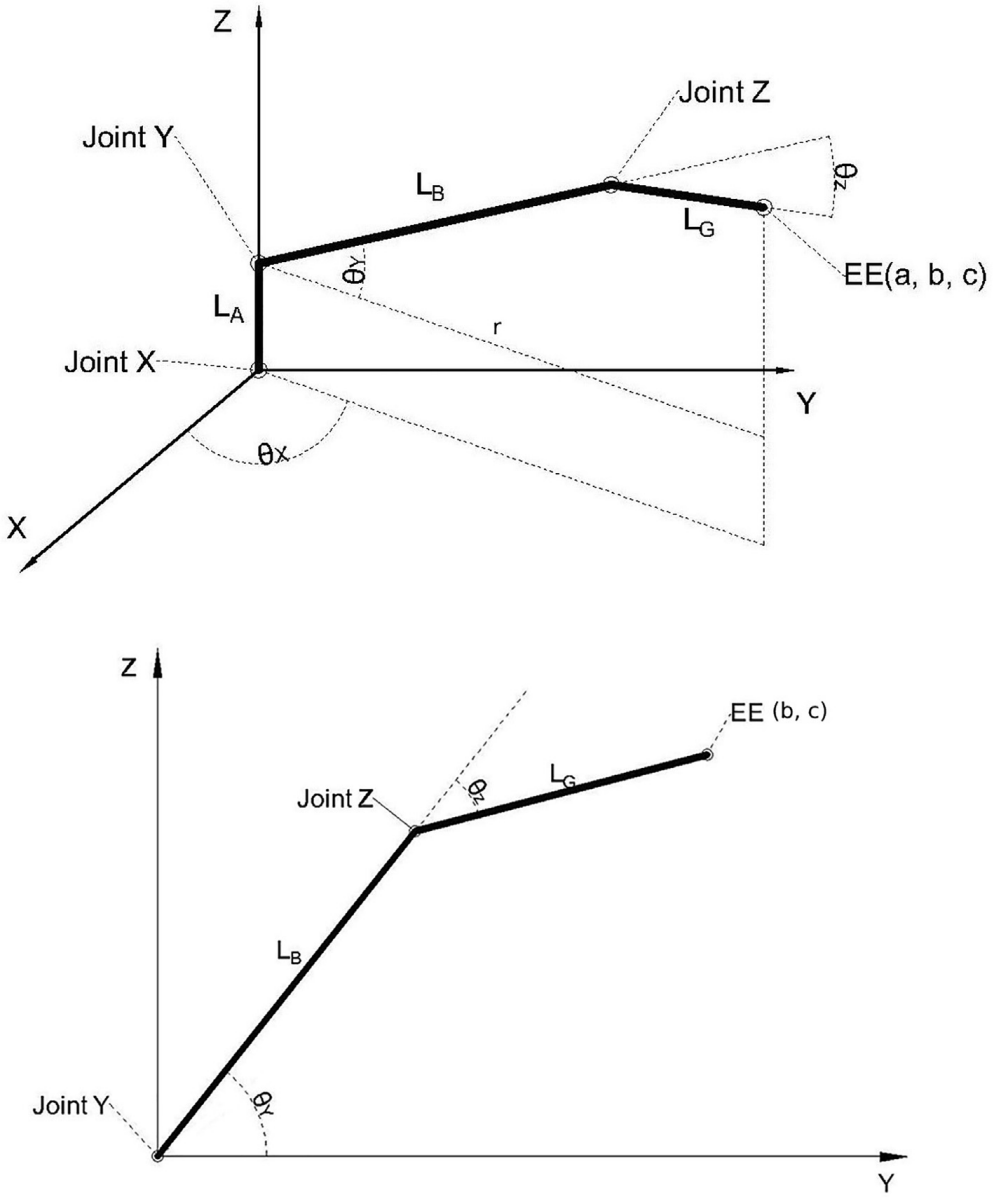
Analysis of X, Y, an Z joint angles in 3D space (top) and simplification to just Y and Z joints in 2D space (bottom).

Firstly, as joint X just rotates the arm in the vertical plane, one can simplfiy this 3D problem into a 2D problem by just analyzing the Y, Z joints (Fig. 22).

Consulting Fig. 22, b,c denote the y, z coordinates of the end effector in the 2D space. One can easily generate functions for the coordinates of the end effector:

$$\begin{array}{cc} b= & \cos\theta_{Y}\cdot L_{B}+\cos(\theta_{Y}-\theta_{Z})\cdot L_{G}\\ c= & \sin\theta_{Y}\cdot L_{B}+\sin(\theta_{Y}-\theta_{Z})\cdot L_{G} \end{array}$$

Now, back to the 3D space problem. In this case, parameters a, b, c denote the x, y, z coordinates of the end effector in 3D space.

$$\begin{array}{cc} r= & \cos\theta_{Y}\cdot L_{B}+\cos(\theta_{Y}-\theta_{Z})\cdot L_{G}\\ a= & \cos\theta_{X}\cdot r\\ b= & \sin\theta_{X}\cdot r\\ c= & L_{A}+\sin\theta_{Y}\cdot L_{B}+\sin(\theta_{Y}-\theta_{Z})\cdot L_{G} \end{array}$$

One can also compute the position of the end effector frame via linear algebra. Using URDF notation:

• ${}^{B}T_{\mathrm{ee}}$: The transformation matrix from base-link’s coordinate to the end effector’s.
• $q_{i}$: The joint value for joint i
• $T_{L_{B}}$: The translation of base-link
• $T_{L_{i}}$: The translation of link i
• $T_{J_{i}}$: The rotation matrix of joint i
• $\theta_{i}$: the rotation angle of each joint

Thus:

$${}^{B}T_{\mathrm{ee}}=T_{L_{B}}\;\cdot \;T_{J_{X}}(q_{X})\;\cdot \;T_{L_{a}}\;\cdot \;T_{J_{Y}}(q_{Y})\;\cdot \;T_{L_{b}}\;\cdot \;T_{J_{Z}}(q_{Z})\;\cdot \;T_{L_{g}}$$

Based on the inter-link translatio n and rotation matrices contained in the URDF file:

**Figure:**
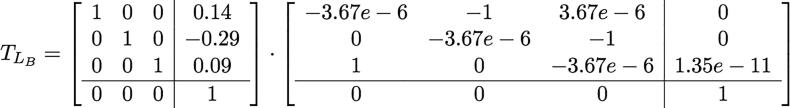

**Figure:**
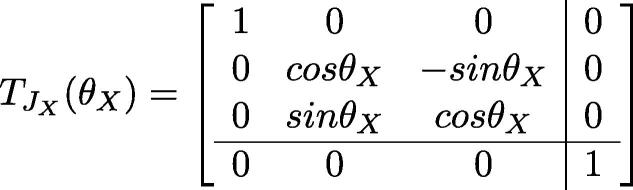

**Figure:**
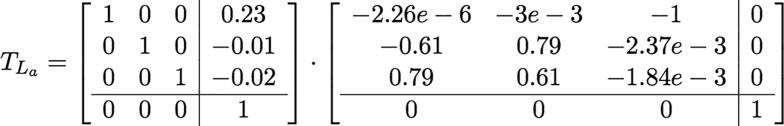

**Figure:**
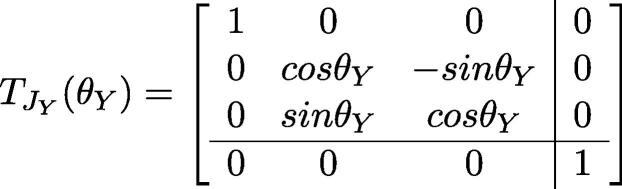

**Figure:**
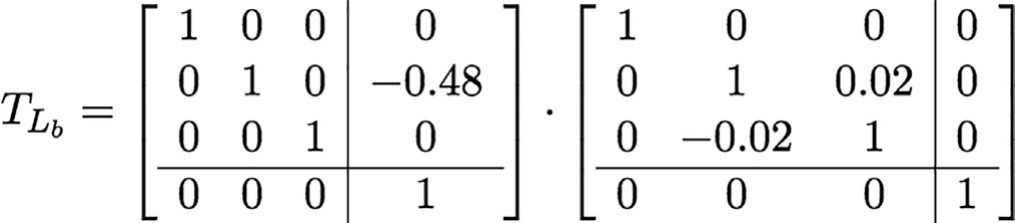

**Figure:**
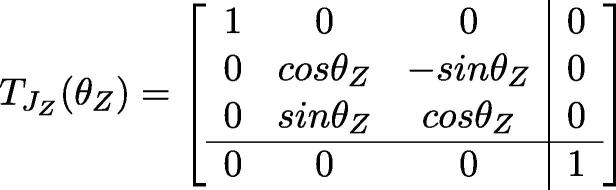

**Figure:**
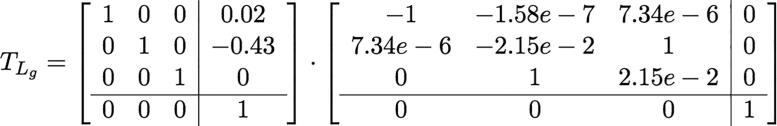

#### 2.7.3 Inverse kinematics

Simplifying the problem to the 2D plane defined by the Y and Z linkages again, the end effector’s position X$\in R^{2}$. Suppose once more X=(b,c) and $L_{B},L_{G}$ denote the length of Bicep Link and Forearm Link, as in Fig. 22. The aim is to compute the value of $\theta \in R^{2}$ which can satisfy the arm to achieve the point *X*. One can derive the following relations:

$$r=\sqrt{b^{2}+c^{2}}$$

$$r^{2}={\left(L_{B}+\cos\theta_{Z}\cdot L_{G}\right)}^{2}+{\left(\sin\theta_{Z}\cdot L_{G}\right)}^{2}$$

$$\theta_{Z}=\mathrm{acos}\left(\frac{b^{2}+c^{2}-L_{B}^{2}-L_{G}^{2}}{2L_{B}\cdot L_{G}}\right)$$

$$\theta_{\alpha }=\mathrm{atan}2(c,b)$$

$$\theta_{\beta }=\mathrm{acos}\left(\frac{L_{B}+\cos\theta_{Z}\cdot L_{G}}{r}\right)$$

$$\theta_{Y}=\theta_{\alpha }+\theta_{\beta }$$

So, the problem in 2D is solved. Moving to 3D space, where $X\in R^{3},\theta \in R^{3}$ which satisfies the arm to achieve the point $X\in R^{3}$ must be solved.

Suppose X=(a,b,c), as in Fig. 22:

$$\theta_{X}=\mathrm{atan}2(b,a)$$

Where $L_{A}$ denotes the length of the Central Link. Similarly, one can generate equations to compute $\theta_{Y}$ and $\theta_{Z}$ if *c* is replaced in previous equations with $c-L_{A}$ and *b* is replaced with $\sqrt{a^{2}+b^{2}}$.

Note: These equations use a function called atan2(y,x), which returns the angle from the origin to a point (x,y) in the plane. It is similar to the inverse tangent $\tan^{-1}(\frac{y}{x})$, but whereas $\tan^{-1}(\frac{y}{x}$) is equal to $\tan^{-1}(\frac{-y}{-x})$, and therefore $\tan^{-1}$ only returns angles in the range $\left(\frac{-\pi }{2},\frac{\pi }{2}\right)$, the atan2 function returns angles in the range (-π,π).

## 3 Design files

### 3.1 Design files summary

**Table:** 

**Design filename**	**File type**	**Open source license**	**Location of the file**
*PARA CAD Assembly*	*.SLDPRT and.SLDASM CAD files*	*MIT*	https://doi.org/10.17605/OSF.IO/5AF4V
*PARA.STL Files*	*.STL files*	*MIT*	https://doi.org/10.17605/OSF.IO/5AF4V
*PARA.URDF Files*	*.URDF and.STL files*	*MIT*	https://doi.org/10.17605/OSF.IO/5AF4V
*PARA Servo Tune Files*	*.MTR files*	*MIT*	https://doi.org/10.17605/OSF.IO/5AF4V
*PARA*_*CartesianControlsDemo*	*.PY file*	*MIT*	https://doi.org/10.17605/OSF.IO/5AF4V
*Config*	*.TXT file*	*MIT*	https://doi.org/10.17605/OSF.IO/5AF4V

***"PARA CAD Assembly"** contains the complete CAD model of PARA as constructed in SolidWorks. For those without access to the SolidWorks software, Grabcad*[5]*offers free conversion services from sldprt to formats such as STEP and IGES*.

***"PARA.STL Files"*** contains *.STL files of all the PARA components that require 3D printing.*

***"PARA.URDF Files"** contains a .URDF file of the three-axis PARA for use with software packages with kinetic-simulation capabilities like PyBullet or ROS. It also contains .STL files for visual representation of PARA’s linkages*.

***"PARA Servo Tune Files"** contains .MTR configuration files used with the Teknic Clearview Clearpath motor tuning software*.

***"PARA*****_*****CartesianControlsDemo"** is a Python script which demonstrates serial communication with a Smoothieboard and implementing commands for end effector cartesian controls*.

***".Config"** is a .txt file used to provide a Smoothieboard with the motor steps per mm travel and acceleration values used with the version of PARA presented in this article*.

## 4 Bill of materials

PARA’s components can be split into off-the-shelf purchased components and custom printed components. All components listed total around $3400.

### 4.1 Purchased components

**Table:** 

**Component**	**QTY**	**Cost/Unit**	**Total Cost**	**Source**	**Type**
Mounted Roller Bearing with Two-Bolt Flange (for 1/2” Shaft Diameter)	2	$18.24	$36.48	https://www.mcmaster.com/1434k6	Metal
2024 Aluminum Keyed Rotary Shaft (Fully Keyed, 1/2” Diameter, 6” Long)	3	$13.67	$41.01	https://www.mcmaster.com/1570k41	Metal
Needle-Roller Thrust Bearing (for 1/2” Shaft Diameter, 15/16” OD)	2	$3.33	$6.66	https://www.mcmaster.com/5909k31	Metal
0.032” Thick Washer for 1/2” Shaft Diameter Needle-Roller Thrust Bearing	4	$1.10	$4.40	https://www.mcmaster.com/5909k44	Metal
Ball Bearing (Open, Trade Number R8, for 1/2” Shaft Diameter)	7	$6.27	$43.89	https://www.mcmaster.com/60355k505	Metal
L Series Timing Belt, Trade No. 420L100	1	$38.66	$38.66	https://www.mcmaster.com/6484k358	Polymer
L Series Timing Belt, Trade No. 158L100	5	$18.44	$92.20	https://www.mcmaster.com/6484k717	Polymer
Low-Profile Mounted Sealed Steel Ball Bearing (Two-Bolt Flange, for 1/2” Shaft Diameter, Fixed Alignment)	2	$33.30	$66.60	https://www.mcmaster.com/7208k52	Metal
304 Stainless Steel Keyed Rotary Shaft (Fully Keyed, 1/2” Diameter, 9” Long)	1	$20.99	$20.99	https://www.mcmaster.com/7398k6	Metal
316 Stainless Steel Washer (for Number 10 Screw Size, 0.203” ID, 0.438” OD)	31	$0.04	$1.13	https://www.mcmaster.com/90107a011	Metal
316 Stainless Steel Washer (for 1/4” Screw Size, 0.281” ID, 0.625” OD)	36	$0.07	$2.56	https://www.mcmaster.com/90107a029	Metal
316 Stainless Steel Washer (for 5/8” Screw Size, 0.688” ID, 1.5” OD)	1	$0.63	$0.63	https://www.mcmaster.com/90107a035	Metal
316 Stainless Steel Washer (for 3/8” Screw Size, 0.406” ID, 0.75” OD)	8	$0.35	$2.82	https://www.mcmaster.com/90107a127	Metal
Zinc-Plated Alloy Steel Socket Head Screw (10–24 Thread Size, 5/8” Long)	21	$0.14	$3.01	https://www.mcmaster.com/90128a221	Metal
Zinc-Plated Alloy Steel Socket Head Screw (10–24 Thread Size, 1–1/2” Long)	22	$0.22	$4.76	https://www.mcmaster.com/90128a226	Metal
Zinc-Plated Alloy Steel Socket Head Screw (1/4”-20 Thread Size, 1” Long)	46	$0.25	$11.72	https://www.mcmaster.com/90128a247	Metal
Zinc-Plated Alloy Steel Socket Head Screw (7/16”-14 Thread Size, 1–1/4” Long)	12	$0.86	$10.36	https://www.mcmaster.com/90128a406	Metal
Zinc-Plated Alloy Steel Socket Head Screw (3/8”-16 Thread Size, 1” Long)	4	$0.51	$2.05	https://www.mcmaster.com/90128a624	Metal
Low-Strength Steel Thin Nylon-Insert Locknut (Zinc-Plated, 7/16”-14 Thread Size)	4	$0.07	$0.27	https://www.mcmaster.com/90566a032	Metal
Low-Strength Steel Thin Nylon-Insert Locknut (Zinc-Plated, 5/8”-11 Thread Size)	1	$0.21	$0.21	https://www.mcmaster.com/90566a035	Metal
High-Strength Steel Nylon-Insert Locknut (Grade 8, 7/16”-14 Thread Size)	4	$0.39	$1.56	https://www.mcmaster.com/90630a112	Metal
High-Strength Steel Nylon-Insert Locknut (Grade 8, 3/8”-16 Thread Size)	9	$0.16	$1.44	https://www.mcmaster.com/90630a121	Metal
Low-Strength Steel Nylon-Insert Locknut (Zinc-Plated, 10–24 Thread Size)	16	$0.03	$0.53	https://www.mcmaster.com/90631a011	Metal
Low-Strength Steel Nylon-Insert Locknut (Zinc-Plated, 10–24 Thread Size)	6	$0.03	$0.19	https://www.mcmaster.com/90633A011	Metal
Black-Oxide Alloy Steel Socket Head Screw (5/8”-11 Thread Size, 12” Long)	1	$15.40	$15.40	https://www.mcmaster.com/91251a137	Metal
Alloy Steel Shoulder Screw (1/4” Shoulder Diameter, 5/8” Shoulder Length, 10–24 Thread)	1	$1.21	$1.21	https://www.mcmaster.com/91259A539	Metal
Alloy Steel Shoulder Screw (1/4” Shoulder Diameter, 1” Shoulder Length, 10–24 Thread)	3	$1.31	$3.93	https://www.mcmaster.com/91259A542	Metal
Alloy Steel Shoulder Screw (1/2” Shoulder Diameter, 3–3/4” Shoulder Length, 3/8”-16 Thread)	5	$4.96	$24.80	https://www.mcmaster.com/91259a727	Metal
18–8 Stainless Steel Socket Head Screw (M3.5 x 0.60 mm Thread, 14 mm Long)	5	$2.26	$11.31	https://www.mcmaster.com/91292A351/	Metal
Low-Strength Steel Cap Nut (Zinc-Plated, 1/4”-20 Thread Size)	4	$0.32	$1.29	https://www.mcmaster.com/91875A130	Metal
316 Stainless Steel SAE Washer (for 7/16” Screw Size, 0.469” ID, 0.922” OD)	16	$0.53	$8.52	https://www.mcmaster.com/91950a048	Metal
18–8 Stainless Steel Washer (for 1–3/4” Screw Size, 1.812” ID, 3.5” OD)	2	$2.31	$4.62	https://www.mcmaster.com/92141a044	Metal
18–8 Stainless Steel Cup-Point Set Screw (10–24 Thread, 1/2” Long)	17	$0.06	$1.02	https://www.mcmaster.com/92311a242	Metal
LDPE Unthreaded Spacer (3/4” OD, 3/4” Long, for 1/4” Screw Size)	3	$0.50	$1.51	https://www.mcmaster.com/92825A146	Polymer
LDPE Unthreaded Spacer (3/4” OD, 3/8” Long, for 1/4” Screw Size)	1	$0.39	$0.39	https://www.mcmaster.com/92825A415	Polymer
Tapered Heat-Set Inserts for Plastic (10–24 Thread Size, 0.225” Installed Length, Brass)	39	$0.20	$7.85	https://www.mcmaster.com/93365a150	Metal
Tapered Heat-Set Inserts for Plastic (1/4”-20 Thread Size, 0.3” Installed Length, Brass)	48	$0.40	$19.37	https://www.mcmaster.com/93365a160	Metal
Nylon Hex Nut (M3.5 x 0.6 mm Thread)	3	$0.12	$0.37	https://www.mcmaster.com/93800A450-93800A120/	Polymer
Hardened 440C Stainless Steel Ball (1/2” Diameter)	21	$0.81	$16.95	https://www.mcmaster.com/9529k22	Metal
Medium-Strength Steel Hex Nut (Grade 5, Zinc-Plated, 1/4”-20 Thread Size)	2	$0.05	$0.10	https://www.mcmaster.com/95462a029	Metal
Internal Retaining Ring (for 1–1/8” ID, Black-Phosphate 1060–1090 Spring Steel)	5	$0.24	$1.22	https://www.mcmaster.com/99142a480	Metal
VXB.625 in Square Flanged Cast Housing Mounted Bearing (UCF 201–8)	2	$12.95	$25.90	https://www.vxb.com/1-2-UCF-201-8-Square-Flanged-Cast-Housing-Mounted-p/kit16127.htm?gclid=EAIaIQobChMIr6_Ih4_L6AIVAo3ICh3S-wKDEAYYASABEgKGfPD_BwE	Metal
13 Tooth, 1/2” Inside x 1.522” Outside Diam, Timing Belt Pulley (1” Belt Width, 1.552” Pitch Diam, Steel & Cast Iron)	4	$34.21	$136.84	https://www.mscdirect.com/product/details/35376375	Metal
13 Tooth, 3/8” Inside x 1.522” Outside Diam, Hub & Flange Timing Belt Pulley (1” Belt Width, 1.552” Pitch Diam, 1–1/4” Face Width, Aluminum)	1	$27.10	$27.10	https://www.mscdirect.com/product/details/86235058	Metal
Smoothieboard	1	$94.74	$94.74	https://www.amazon.com/Motherboard-Engraving-Smoothieboard-Firmware-Accessories/dp/B08D75ZZ2H	
CPM-SCHP-2341P-ELNB Servo Motor	1	$631.00	$631.00	https://www.teknic.com/model-info/CPM-SCHP-2341P-ELNB/	Electronic
CPM-SCHP-3441S-ELSB Servo Motor	2	$713.00	$1,426.00	https://www.teknic.com/model-info/CPM-SCHP-3441S-ELSB/	Electronic
IPC-5 75 V Power Supply	1	$248.00	$248.00	https://www.teknic.com/ipc-5/	Electronic
DC Power Cable (CPM-CABLE-PWR-MS120)	1	$19.00	$19.00	https://www.teknic.com/accessories-wizard/	Electronic
24 Volt Power, 2-Pin Molex Cable (CPM-CABLE-M2P2P-120)	2	$19.00	$38.00	https://www.teknic.com/accessories-wizard/	Electronic
Controller Cable (CPM-CABLE-CTRL-MU120)	3	$23.00	$69.00	https://www.teknic.com/clearpath-accessories-guide/accessories-guide-sc-dc/#controller-cables	Electronic
AC Power Cable for IPC-3 and IPC-5 (IPC35-CABLE110)	1	$14.00	$14.00	https://www.teknic.com/clearpath-accessories-guide/accessories-guide-sd-dc/#power-supplies	Electronic
3.25” Diameter 12” Length Acrylic Tube	2	$7.32	$14.64	https://www.usplastic.com/catalog/item.aspx?itemid=32524	Polymer
Base Plate	1	$22.84	$22.84	https://www.homedepot.com/p/1-in-x-2-ft-x-2-ft-Pine-Edge-Glued-Panel-Round-Board-ZPRLR0124/204485287	Wood
Rubber Feet Bumper	6	$2.75	$16.49	https://www.amazon.com/Extra-Large-Round-Rubber-Bumpers/dp/B00S4HVES8	
PLA Filament (1 kg)	6	$19.99	$119.94	https://www.matterhackers.com/store/l/300mm-pla-filament-red-1-kg/sk/MM8DY4PS	Polymer

### 4.2 Printed parts

Note: Recommended support density is 8%. Print times were calculated assuming a 0.6 mm nozzle at 70 mm/s print speed.

**Table:** 

**Part Name**	**QTY**	**Layer Height (mm)**	**Infill %**	**Support?**	**Est. Print Time**	**Print Orientation**

13T Elbow Timing Pulley	1	0.2	80	N/A	2:19:00	
34T Central Link Timing Pulley	1	0.2	60	N/A	10:23:00	
34T Shoulder Timing Pulley	1	0.2	60	N/A	7:45:00	
Base Body	1	0.3	40	Everywhere	22:31:00	
Base Lid	1	0.25	40	Touching Buildplate	11:16:00	
Base Rim	1	0.25	40	N/A	5:15:00	
Base Tensioner Housing	1	0.15	60	Touching Buildplate	2:20:00	
Base Tensioner Spacer	1	0.2	60	N/A	0:47:00	
Base/Central Link Tensioner Body	3	0.15	100	N/A	1:52:00	
Base Joint Inner Lower Race	1	0.15	80	Touching Buildplate	2:27:00	
Base Joint Outer Lower Race	1	0.15	80	Touching Buildplate	5:00:00	
Bicep Link Inboard Cap	1	0.25	60	Touching Buildplate	21:39:00	
Bicep Link Outboard Cap	1	0.25	60	Everywhere	12:53:00	
Bicep Link Tensioner Body	1	0.15	100	N/A	2:39:00	
Bicep Link Tensioner Housing	1	0.15	100	Touching Buildplate	4:40:00	
Central Link Base Plate	2	0.2	60	Touching Buildplate	9:19:00	
Central Link Base Timing Pulley	1	0.2	80	N/A	9:39:00	
Central Link Central Strut	1	0.25	80	N/A	13:25:00	
Central Link Side Plate	2	0.25	40	Everywhere	16:45:00	
Central Link Tensioner Body	2	0.15	100	N/A	2:29:00	
Central Link Tensioner Housing	2	0.15	60	Touching Buildplate	2:20:00	
Compound Timing Pulley	1	0.2	80	N/A	10:04:00	
Drill Jig	1	0.25	40	N/A	5:21:00	
Elbow Joint Coupler	2	0.15	80	N/A	1:27:00	
Electronics Tower Lid	1	0.15	20	Touching Buildplate	9:37:00	
Electronics Tower Spacer	4	0.25	20	N/A	0:04:00	
Forearm Link Inboard Cap	1	0.25	60	Touching Buildplate	15:00:00	
Mounting Plate	1	0.25	60	N/A	8:43:00	
Power Supply Housing	1	0.25	20	N/A	9:08:00	
Smoothieboard Housing	1	0.25	20	N/A	5:00:00	
Timing Pulley Cap	2	0.15	40	Touching Buildplate	4:00:00	

## 5 Build instructions

### 5.1 Safety concerns

Before preparing and assembling PARA’s components, note the following safety concerns. For concerns pertaining to operating PARA once it is built, see Section 6.1.

• Though designed to be minimal, assembling PARA requires some drilling and cutting operations involving hand tools. Wear appropriate personal protective equipment (safety goggles) while performing these operations and ensure that parts are properly secured using clamps or a vice before being cut or drilled.
• NEMA 34 and 23 stepper motors have relatively high input voltages and operating currents compared to most motors used in the consumer electronics and hobby sectors (for example, the motors suggested in this article draw 4 amps per phase at up to 75 volts), thus appropriate caution should be exercised when handling them. Ensure power supply is off and/or disconnected from AC wall supply before plugging and unplugging cables from steppers. Ensure hands are dry when handling power electronics.
• The tip of a soldering iron can reach hundreds of degrees Celsius and leave serious burns if in contact with skin. Exercise caution when using a soldering iron to either connect wire leads or install heat-set inserts.
• Several of PARA’s subassemblies, such as the base and central link, are considerably heavy. If dropped, these parts can result in damages to the hardware, user, or both. During assembly and service, take care to prevent fingers and skin from being pinched near the arm’s joints.
• When assembling PARA, tighten all fasteners until a reasonable amount of resistance is encountered to ensure that the arm doesn’t fall apart during operation. All nuts used on PARA have thread locking features to ensure that they don’t loosen over time, but the arm’s set screws do not. Apply Loctite Threadlocker adhesive to these set screws for more secure retention.

### 5.2 Tools needed

• 1/8” Hex Wrench
• 5/32” Hex Wrench
• 3/16” Hex Wrench
• 1/4” Hex Wrench
• 5/16” Hex Wrench
• 3/8” Hex Wrench
• 1/2” Hex Wrench
• 2.5 mm Hex Wrench
• 7/16” Wrench
• 3/8” Wrench
• 9/16’ Wrench
• Adjustable Wrench
• 1/4” and 1/8” Drill Bits
• 3D Printer (With at least 8” × 8” × 8” buidpltae)
• Soldering Iron
• Hand Saw or Bandsaw
• Rubber Mallet (Helpful)

### 5.3 Build instructions

**Step 1:** Use soldering iron to set heat-set inserts into base lid.

**Figure:**
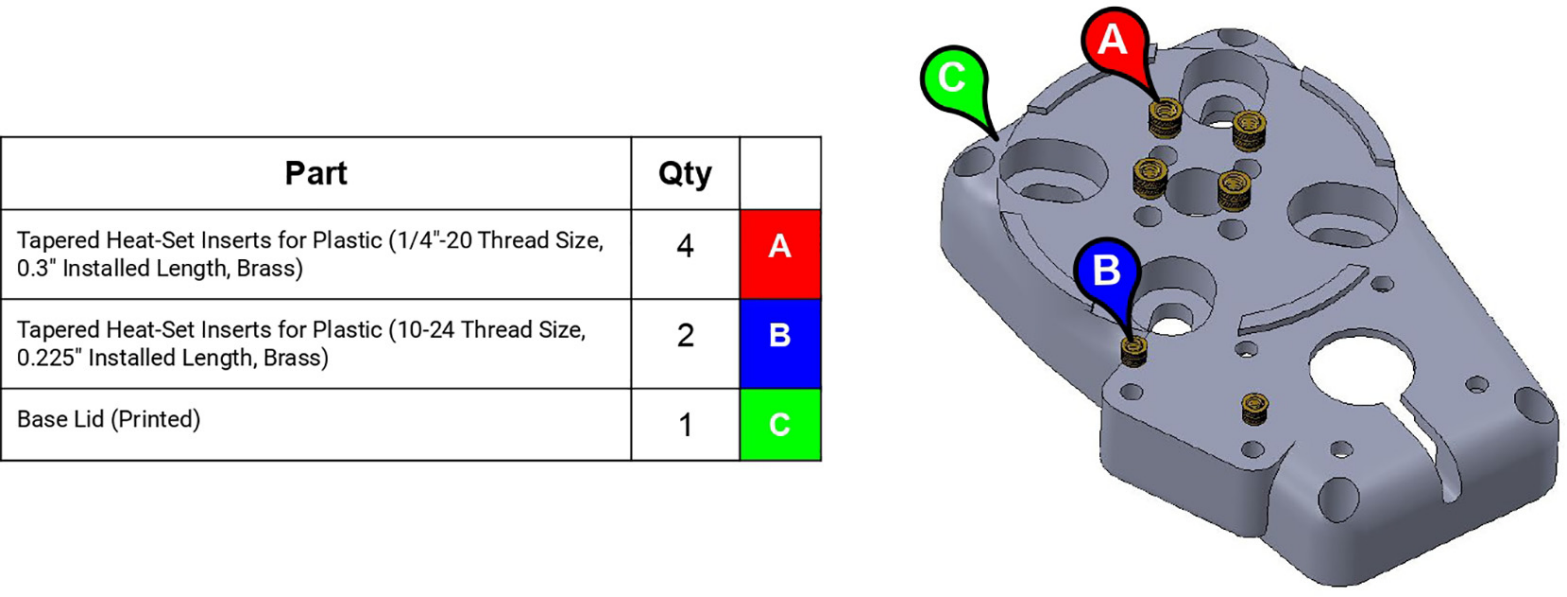

**Step 2:** Fasten servo motor to base lid.

**Figure:**
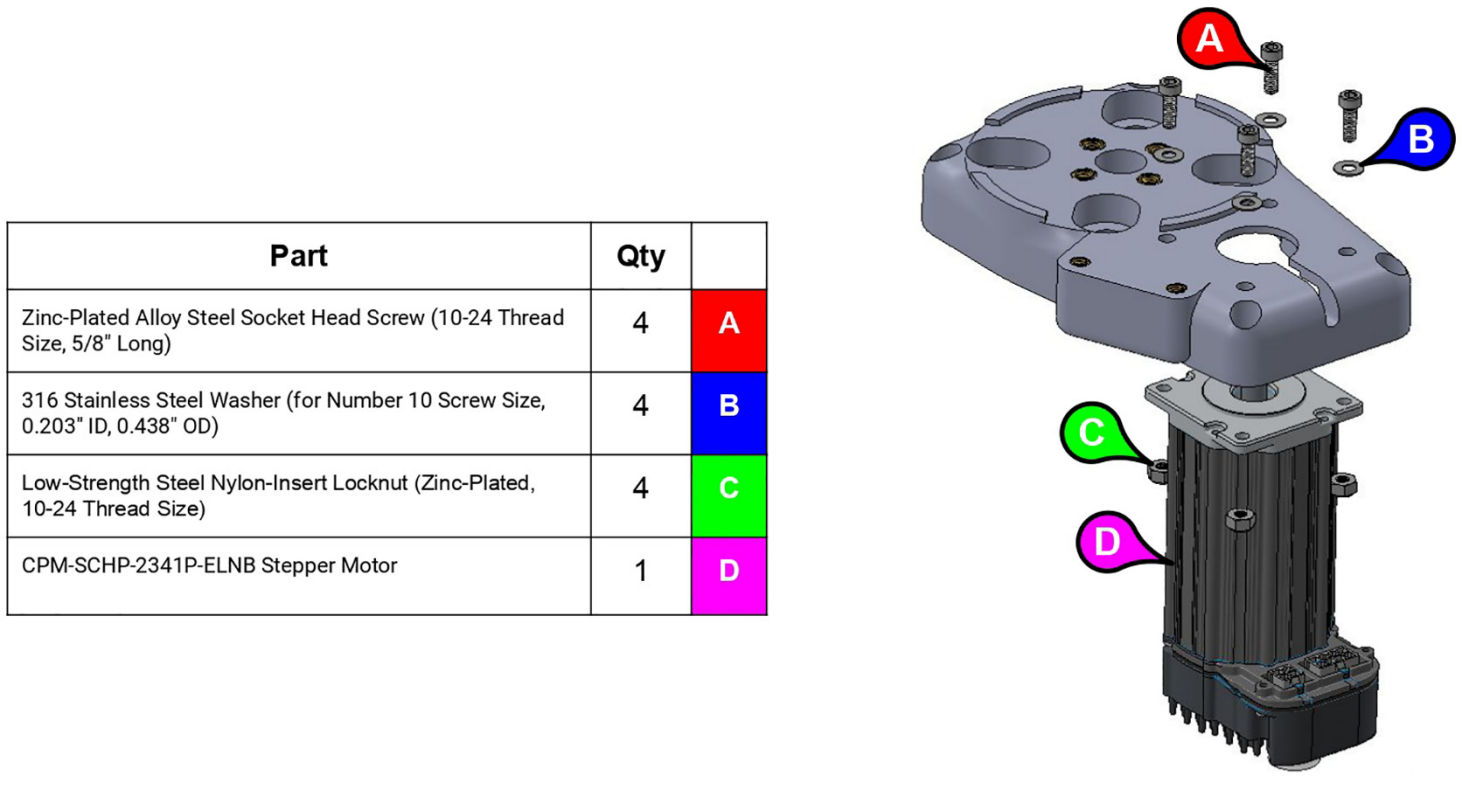

**Step 3:** Fasten square mounted bearing to base lid.

**Figure:**
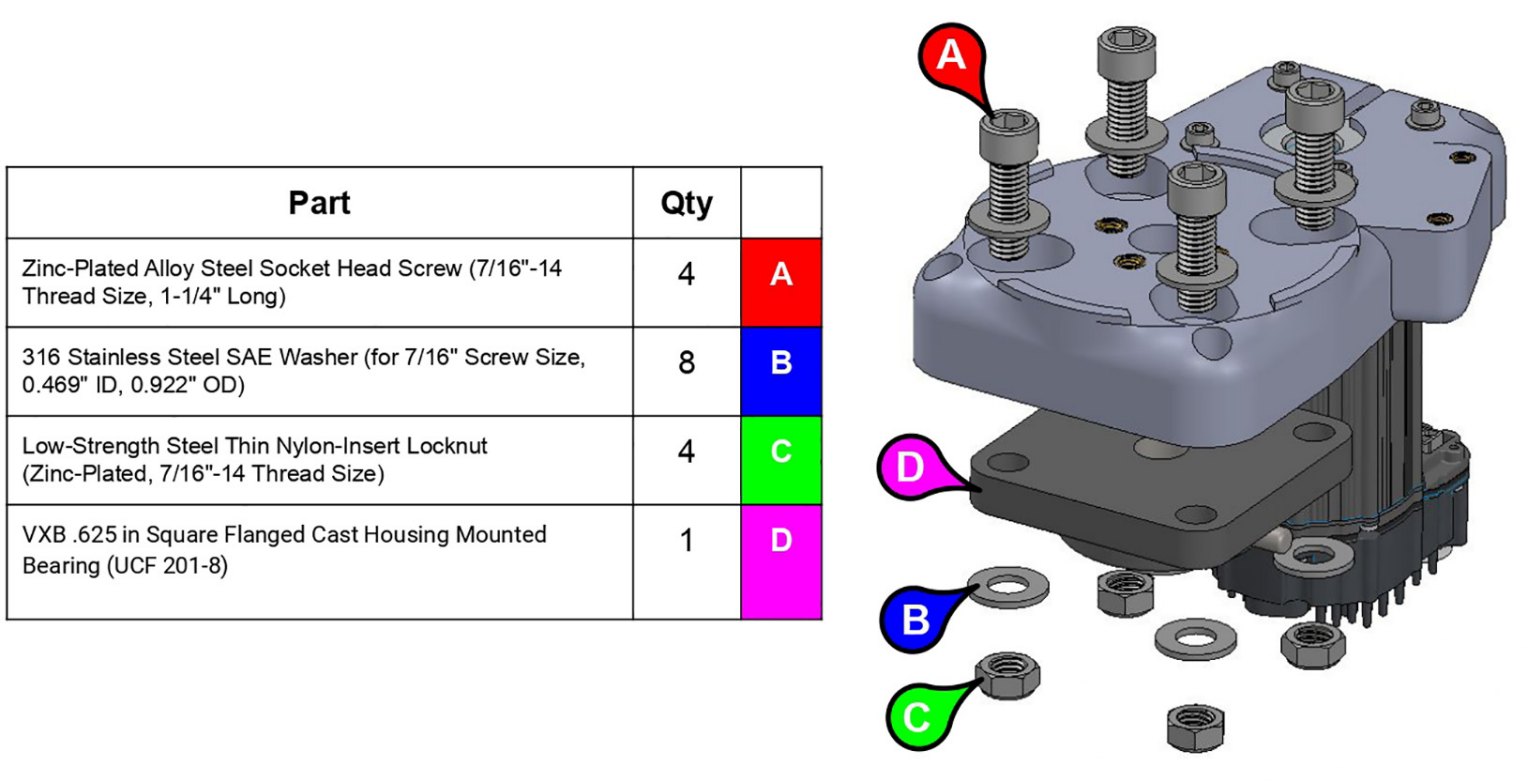

**Step 4:** Use soldering iron to set heat-set inserts into base body.

**Figure:**
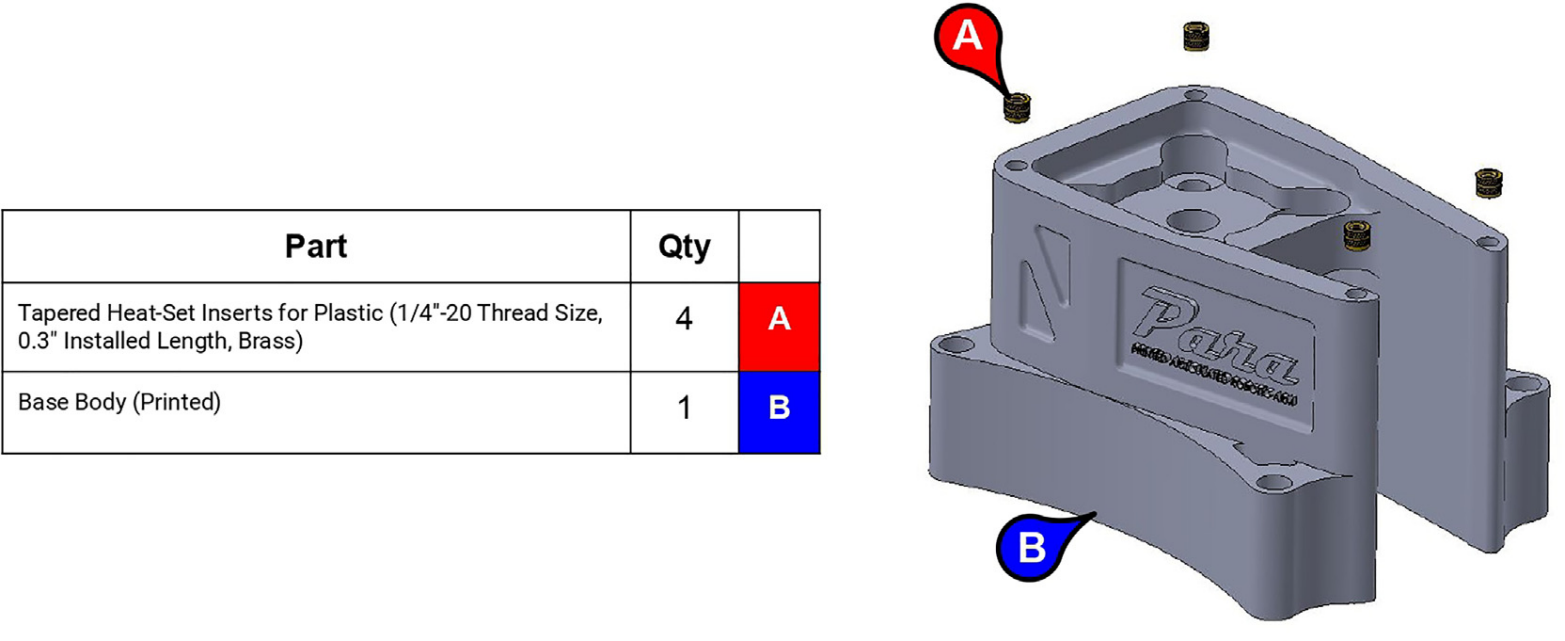

**Step 5:** Fasten square mounted bearing to base body.

**Figure:**
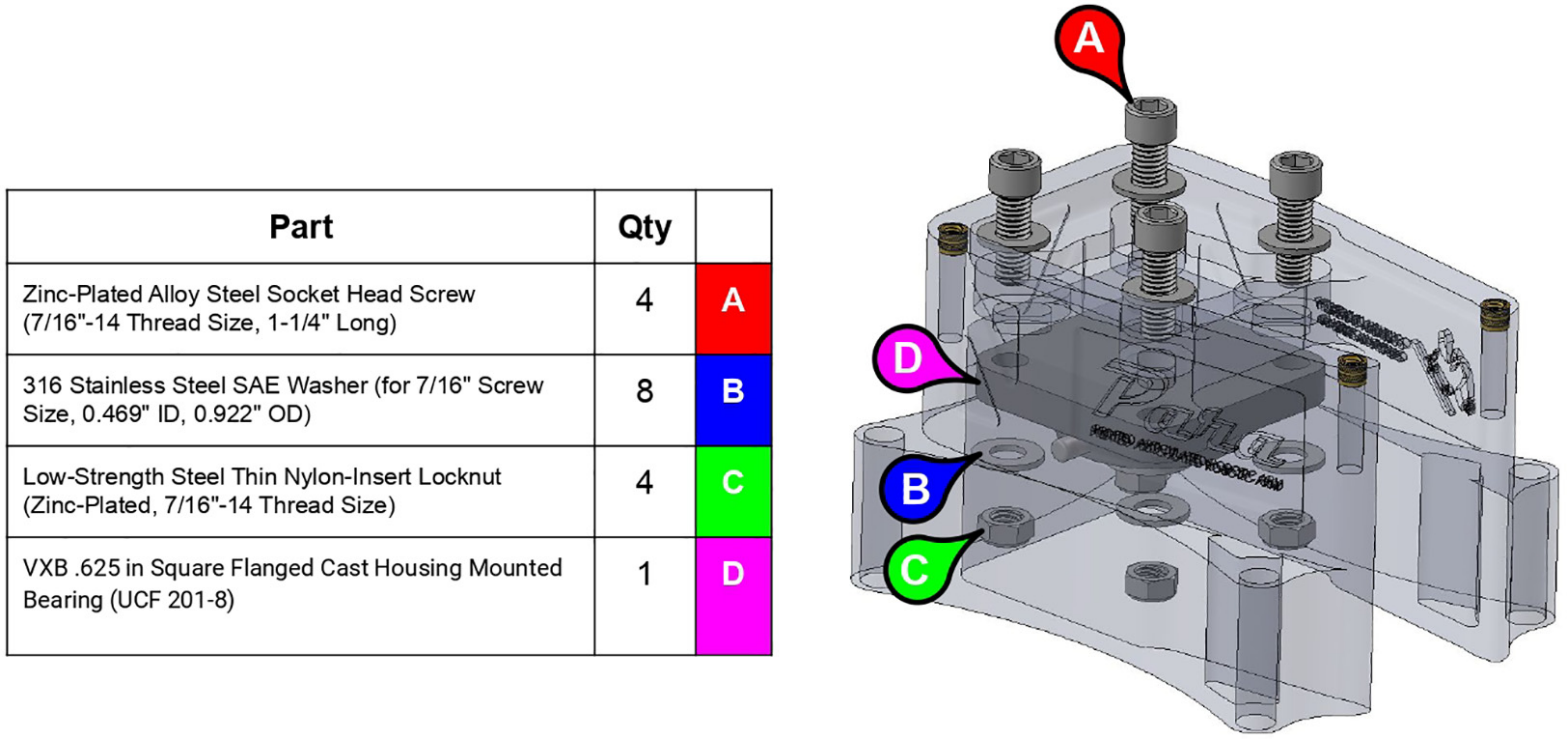

**Step 6:** Fasten assembled base lid onto assembled base body.

**Figure:**
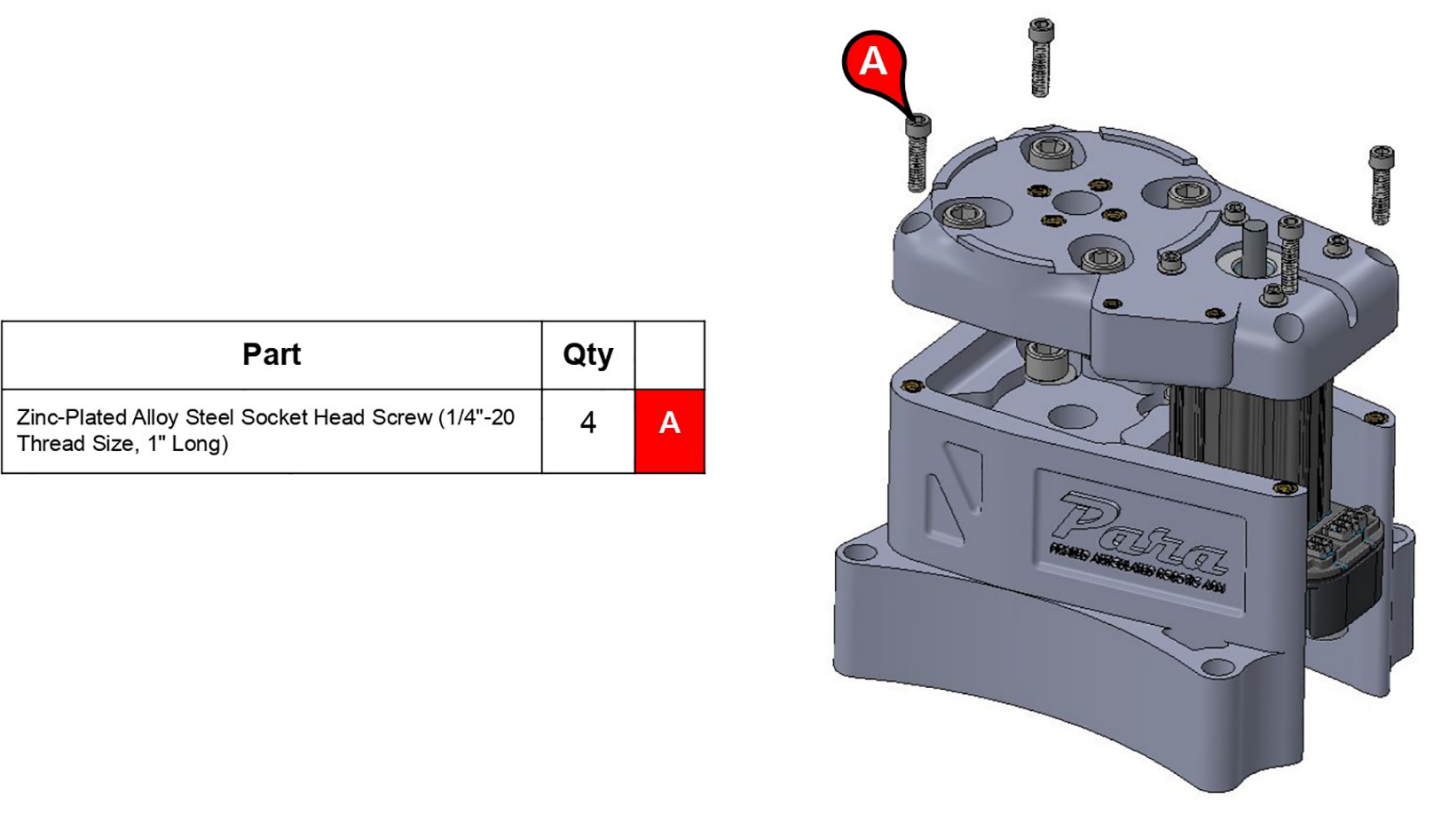

**Step 7:** Sandwich washer between lower race pieces and fasten assembly onto base lid.

**Figure:**
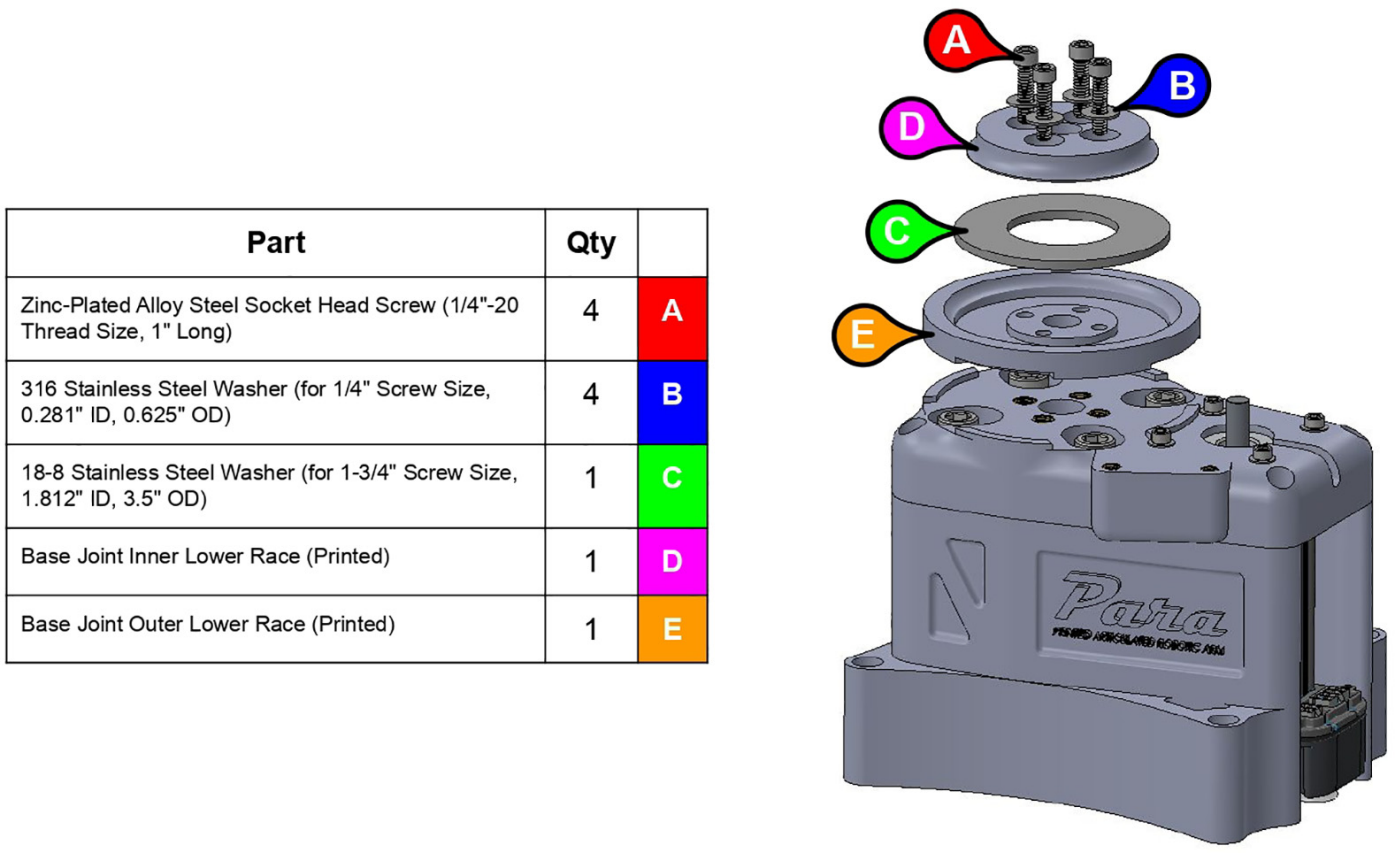

**Step 8:** Insert bolt into mounted bearings and tighten set screws.

**Figure:**
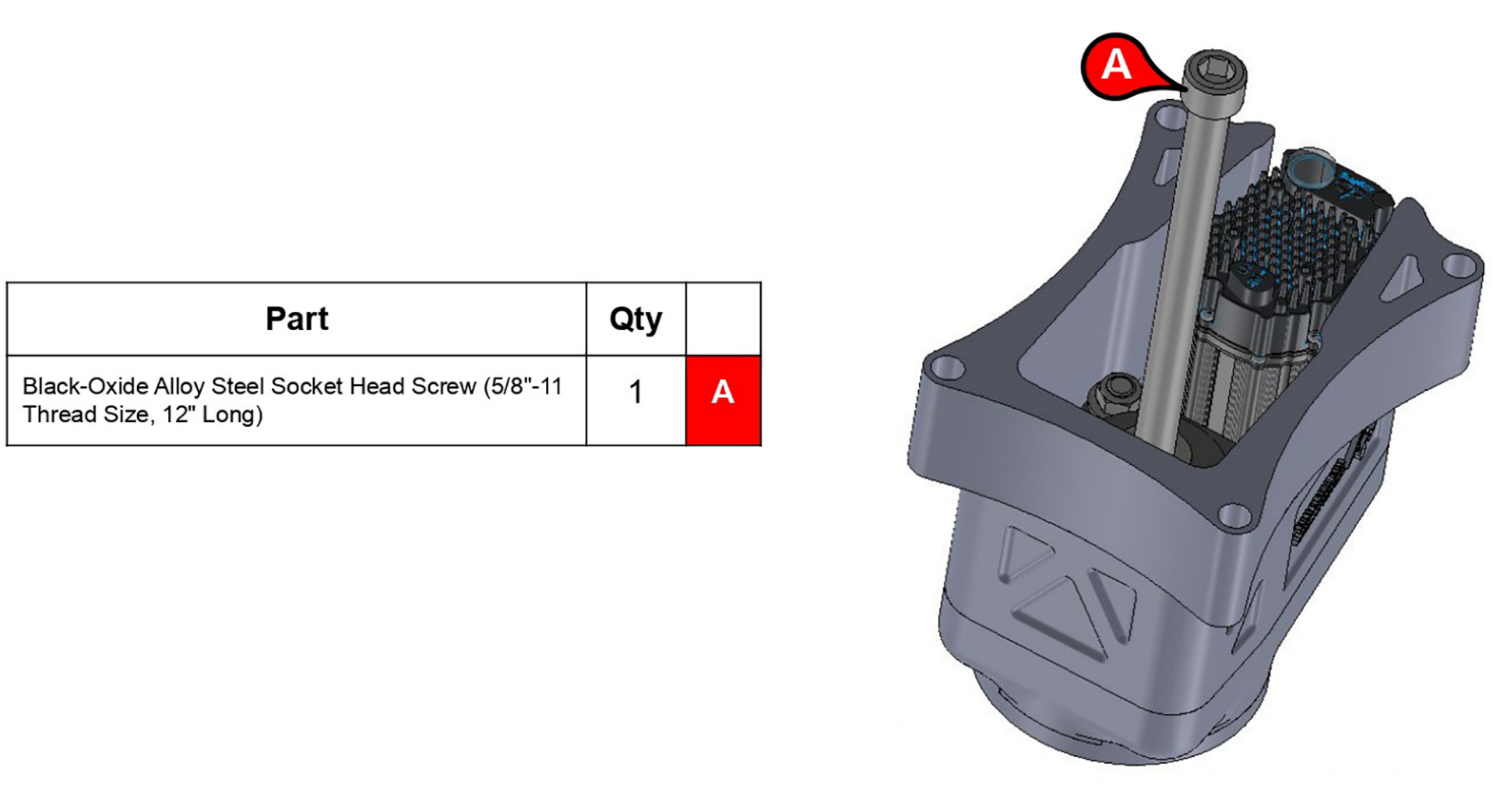

**Step 9:** Drill the indicated hole geometry in a 1” thick base plate.

**Figure:**
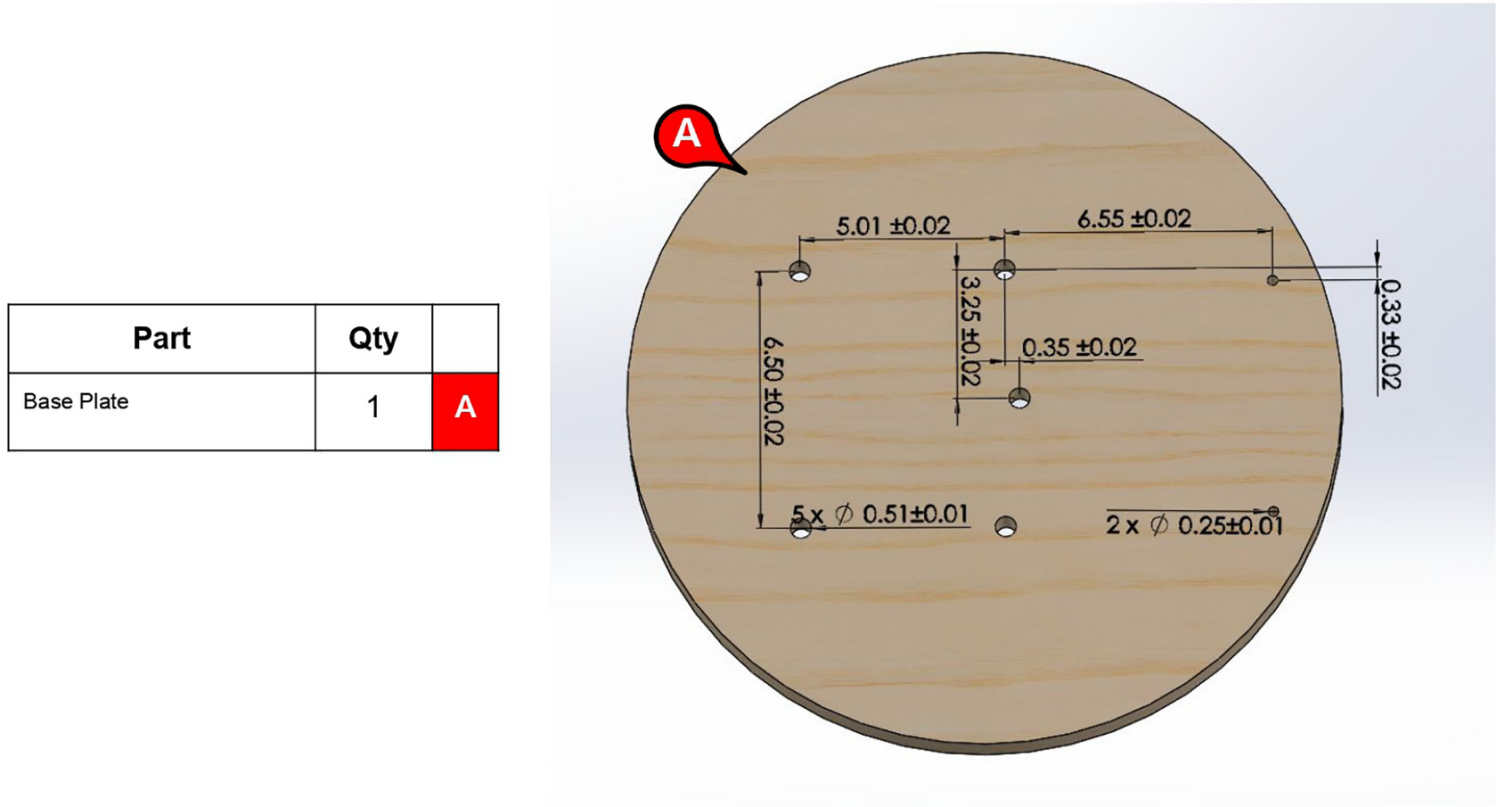

**Step 10:** Fasten base body and base rim to base plate.

**Figure:**
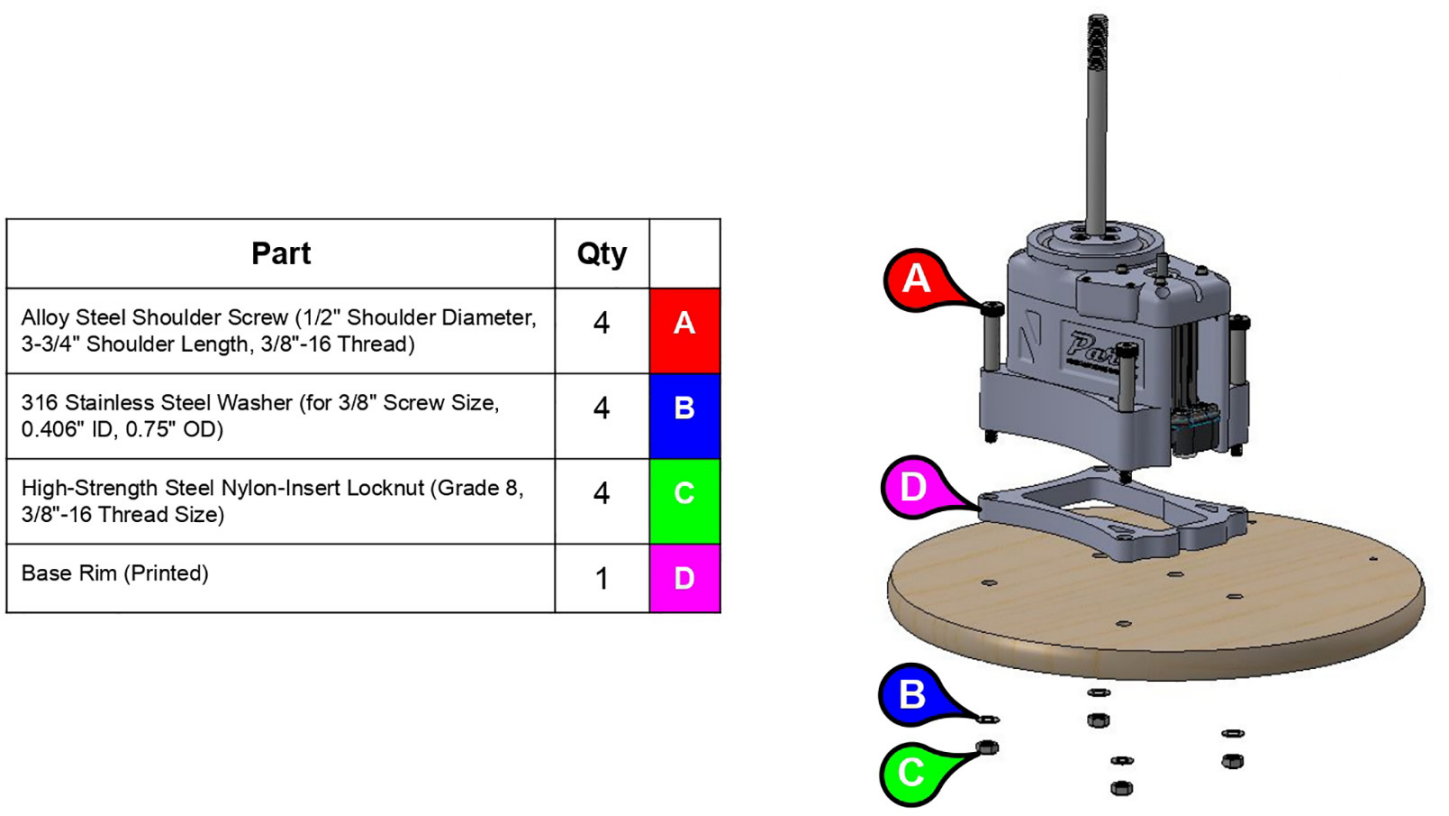

**Step 11:** Fix rubber feet.75” or longer on underside of base.

**Figure:**
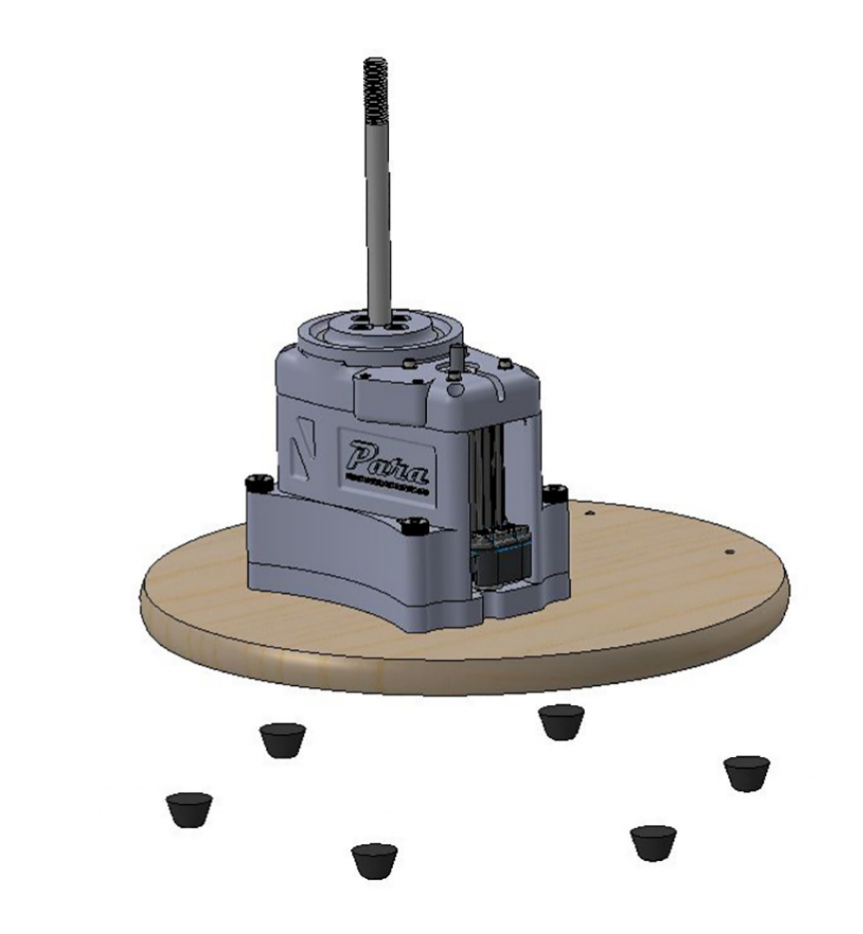

**Step 12:** Use soldering iron to set heat-set inserts into side plates.

**Figure:**
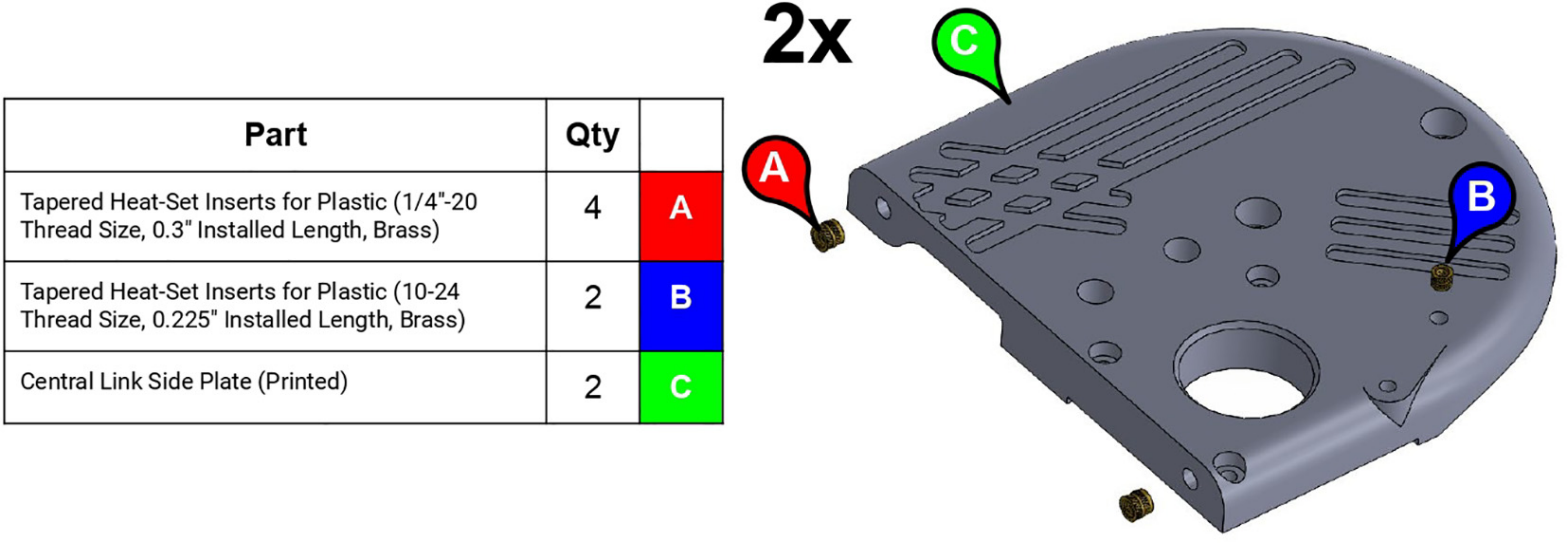

**Step 13:** Press-fit ball bearings into side plates.

**Figure:**
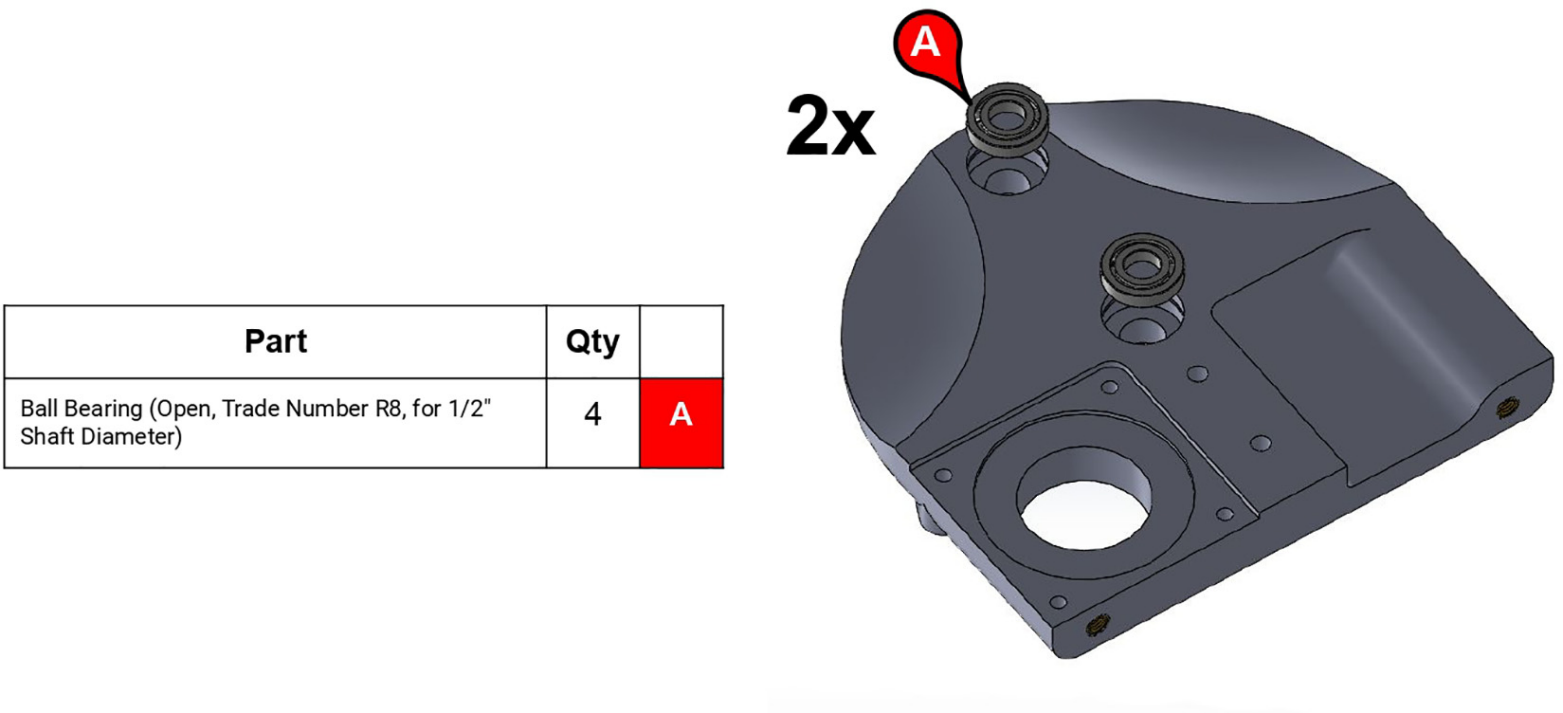

**Step 14:** Snap retaining rings into grooves adjacent to ball bearings.

**Figure:**
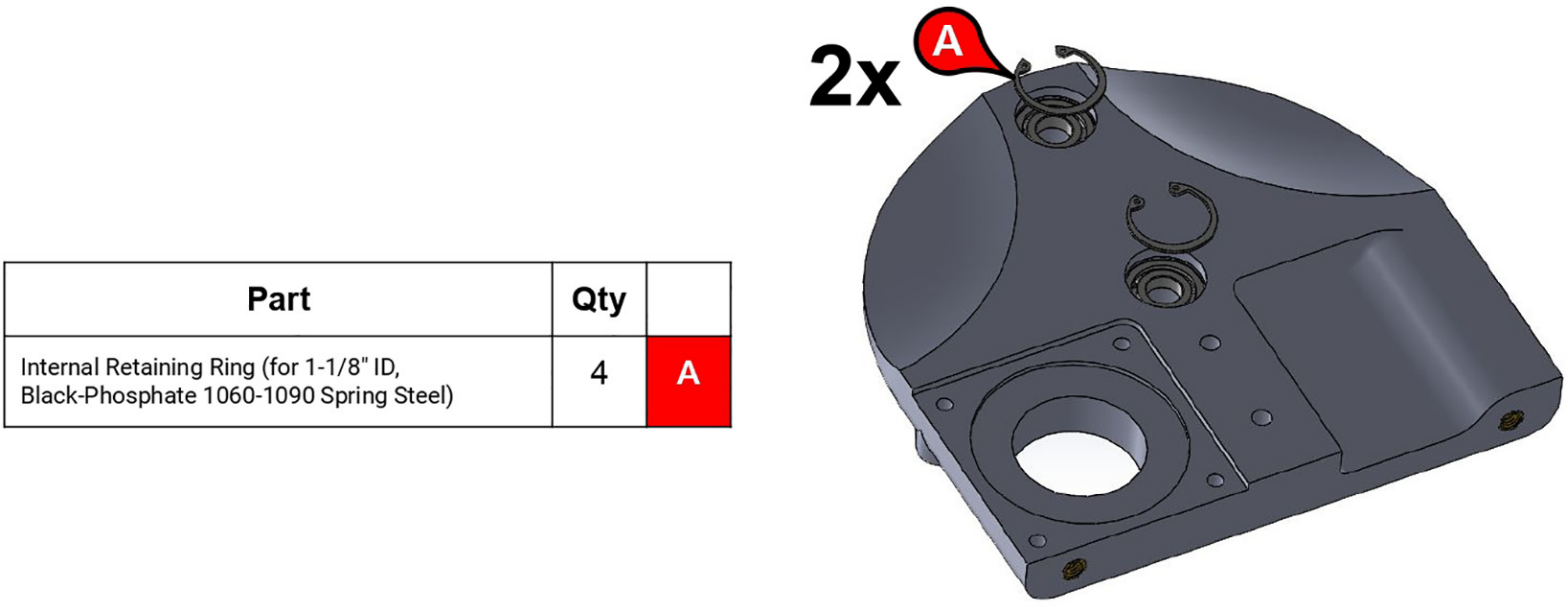

**Step 15:** Fasten motors to side plates.

**Figure:**
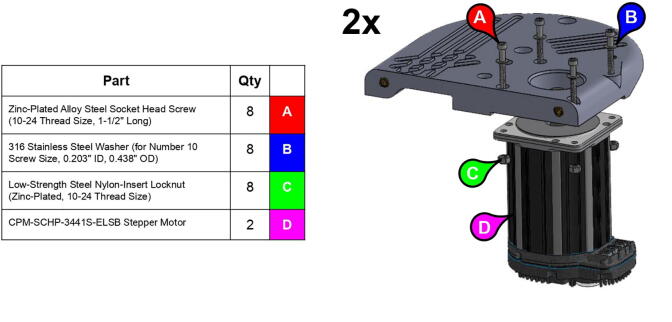

**Step 16:** Use soldering iron to set heat-set inserts into central strut.

**Figure:**
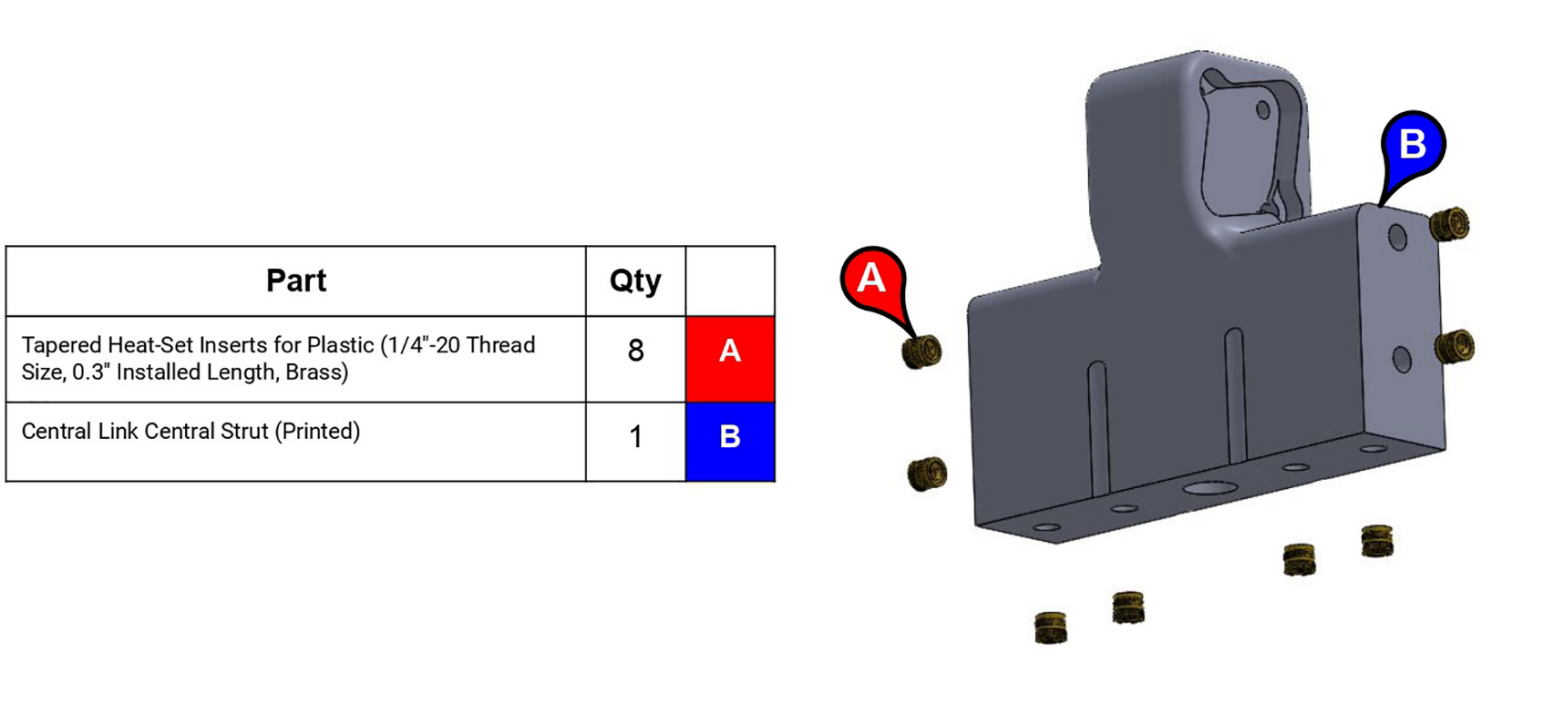

**Step 17:** Fasten roller bearings to central strut. Take care to install fasteners in correct order.

**Figure:**
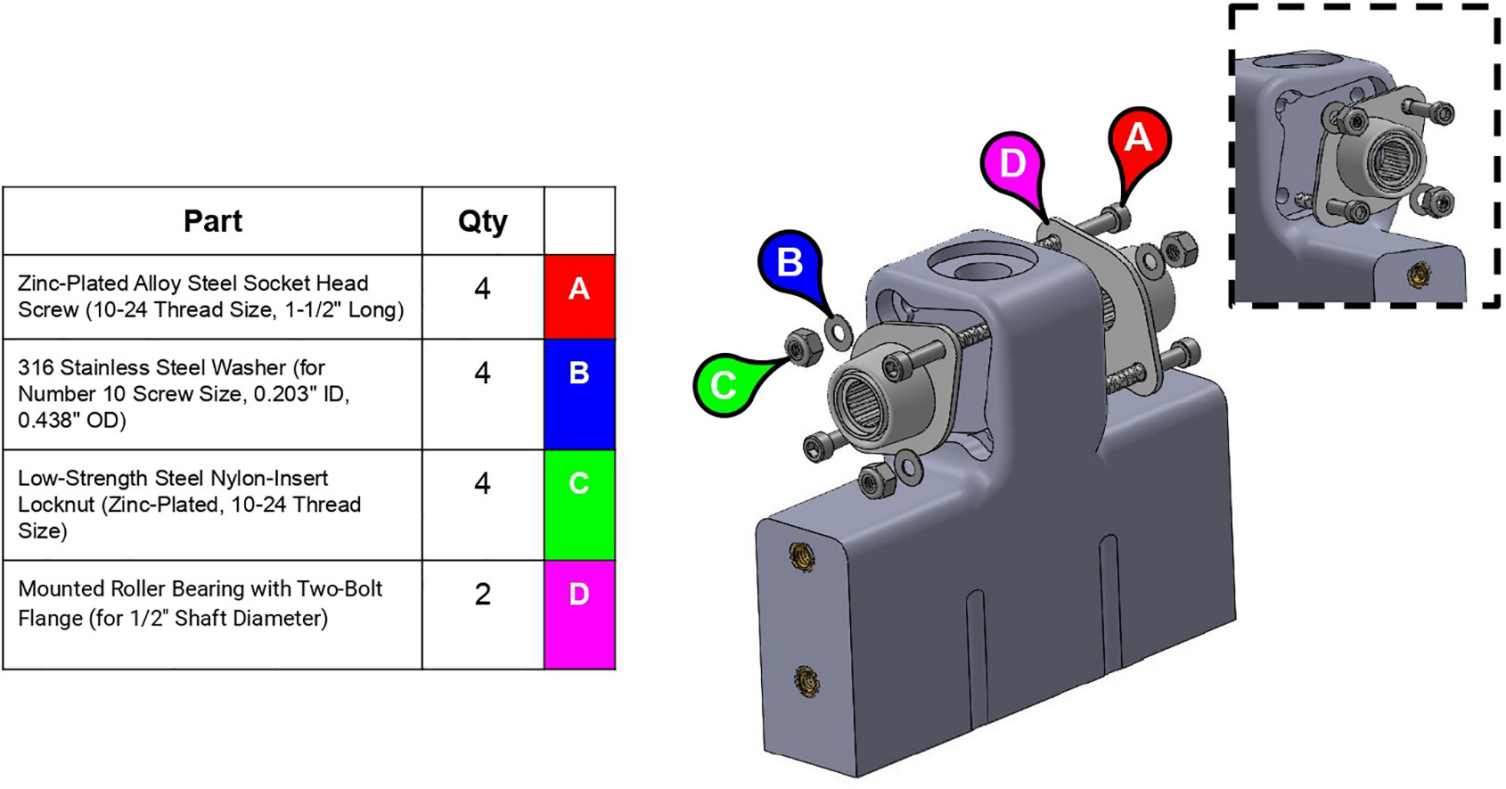

**Step 18:** Fasten base plates to central strut.

**Figure:**
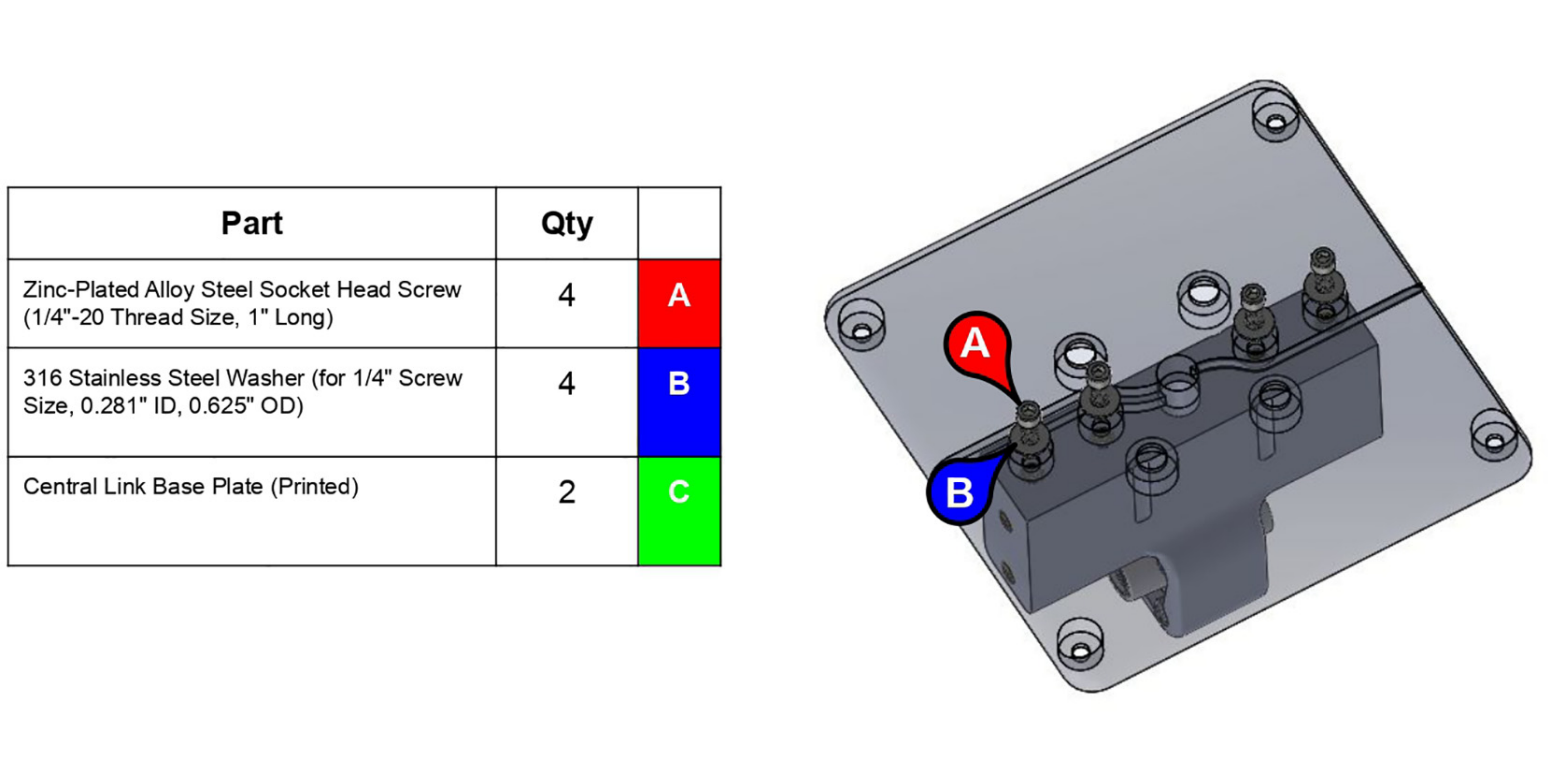

**Step 19:** Slide nuts into sockets of base timing pulley.

**Figure:**
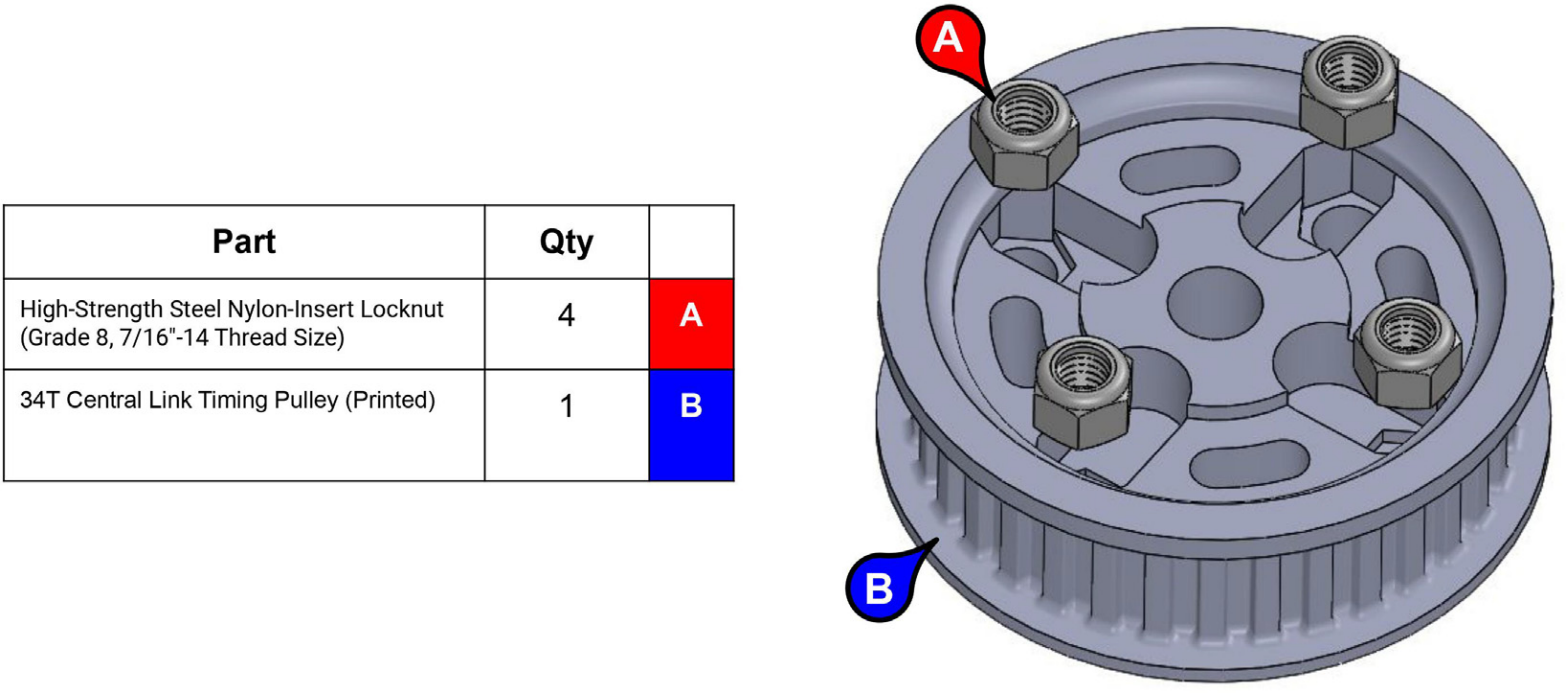

**Step 20:** Press-fit washer onto base timing pulley.

**Figure:**
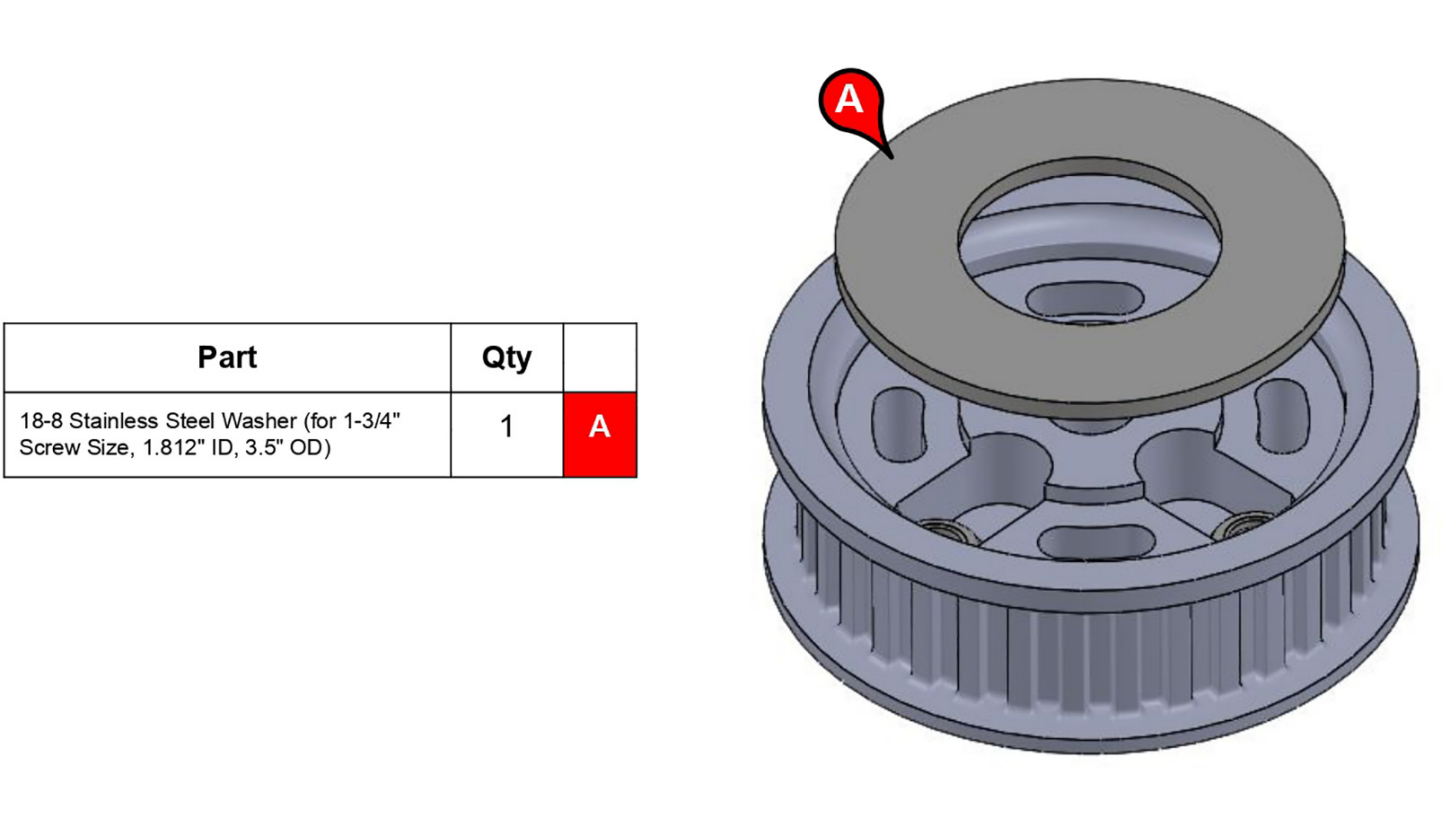

**Step 21:** Place base timing pulley onto base with steel balls in between grooves.

**Figure:**
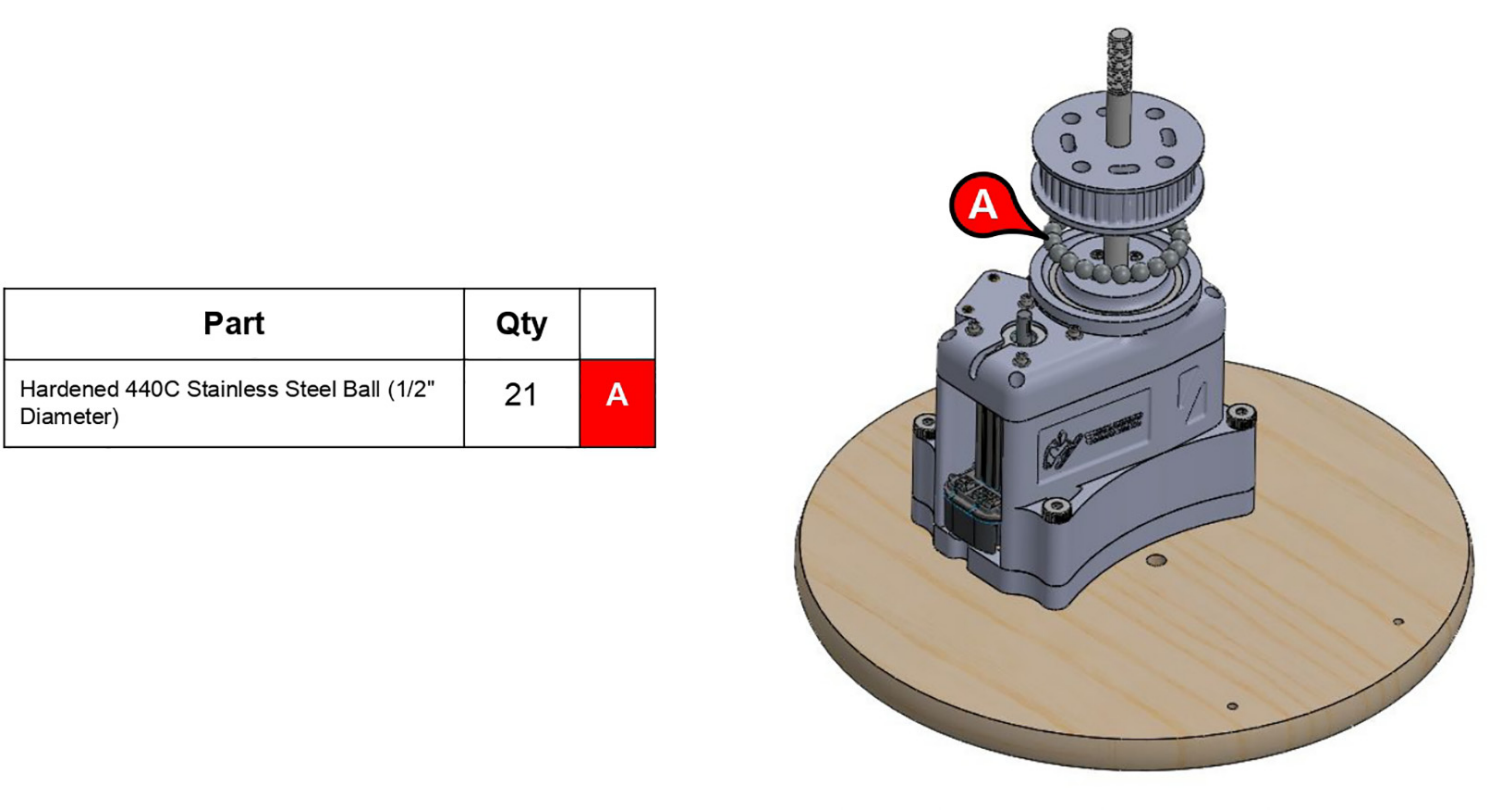

**Step 22:** Place L series timing belt around timing pulleys and fix 13T steel timing pulley to base motor shaft with set screw.

**Figure:**
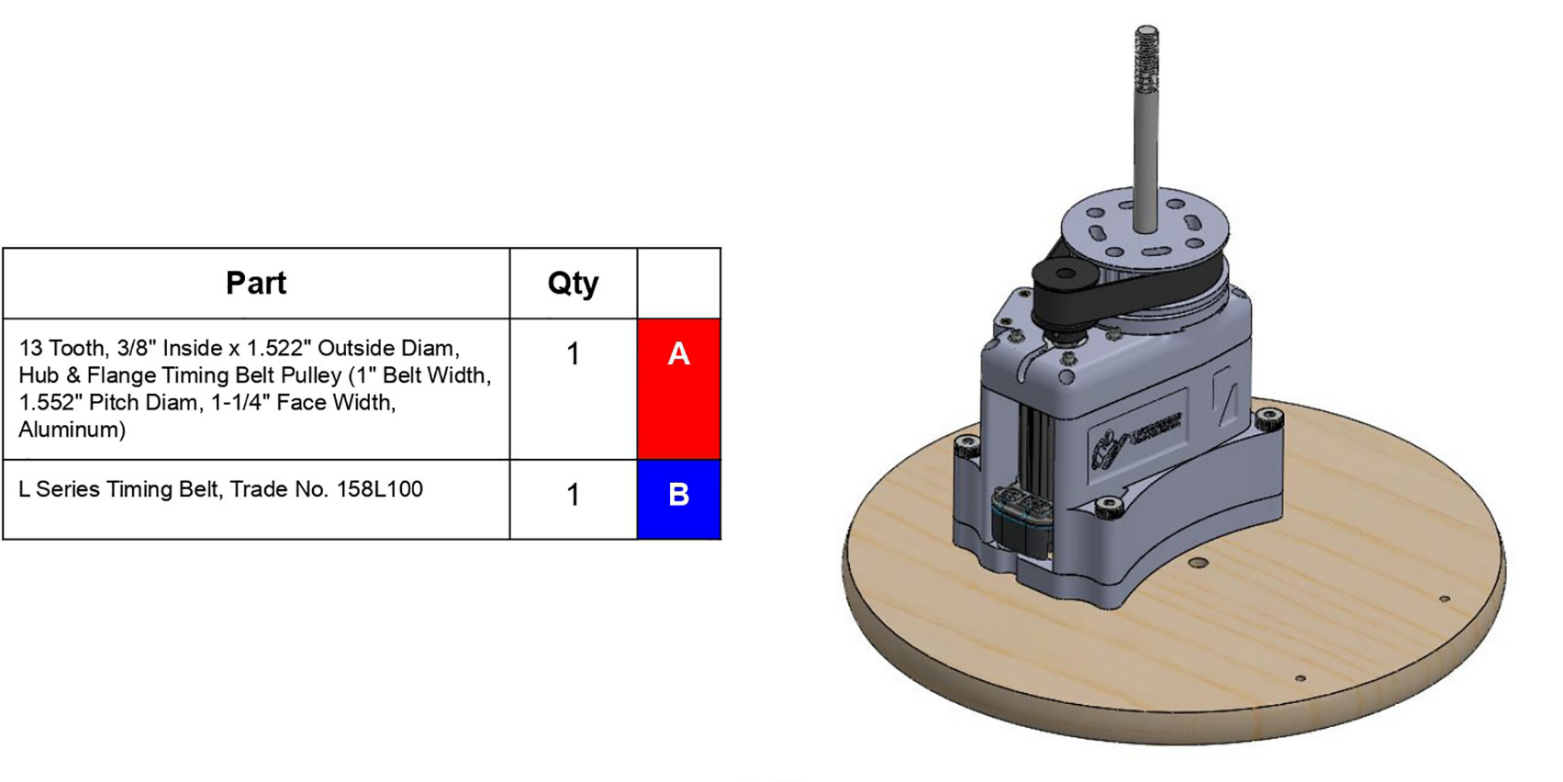

**Step 23:** Use soldering iron to set heat-set inserts into base tensioner housing.

**Figure:**
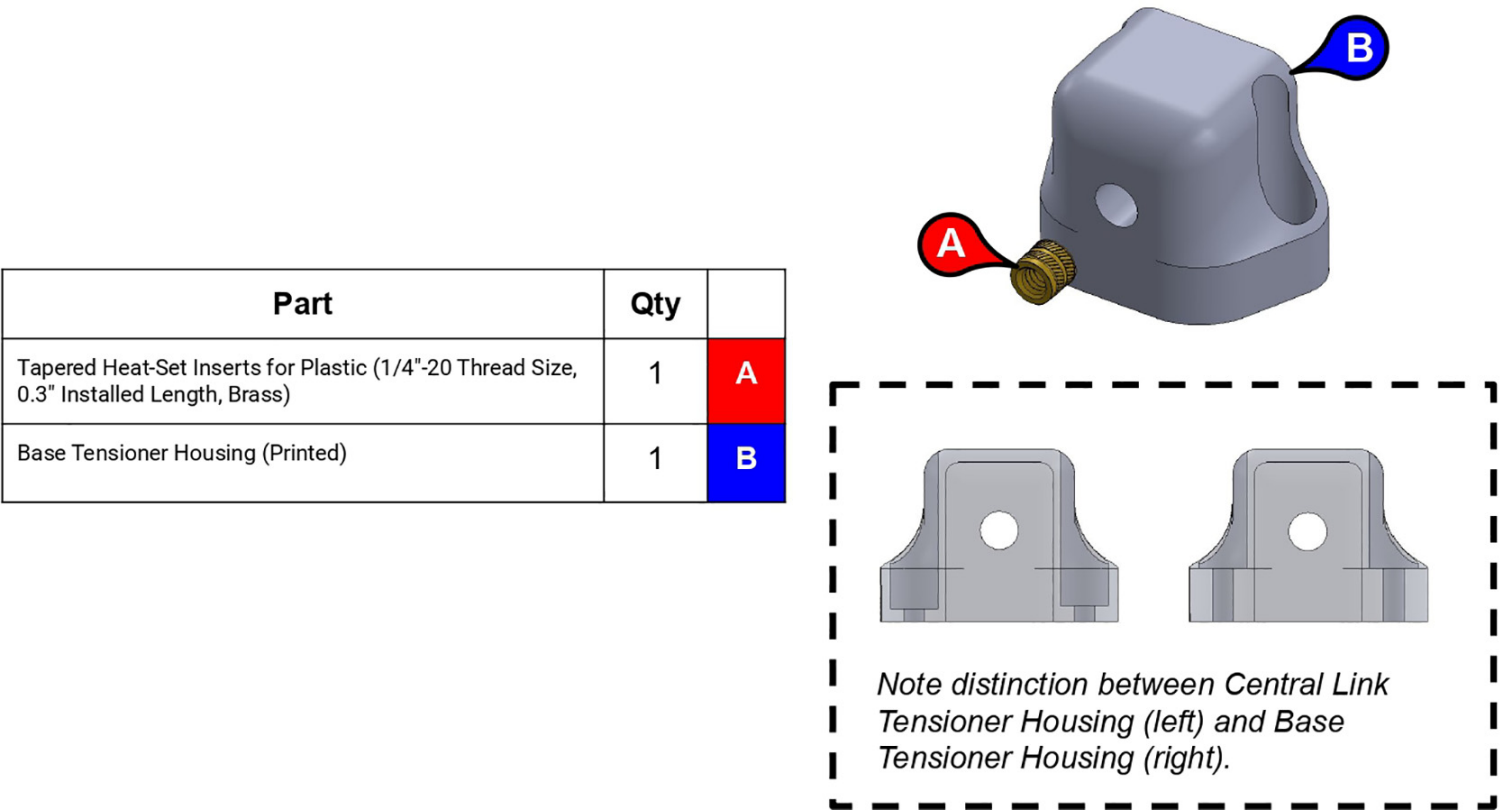

**Step 24:** Assemble various components of base tensioner.

**Figure:**
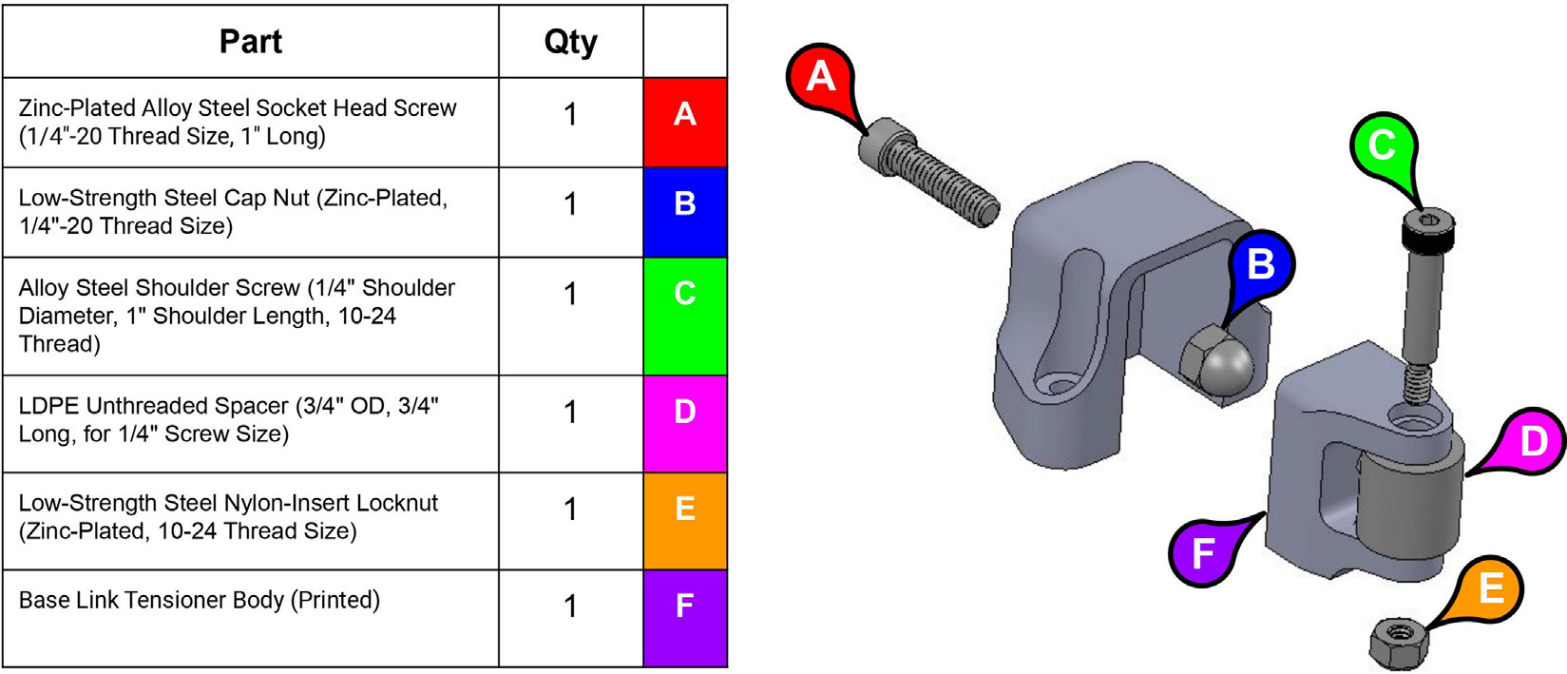

**Step 25:** Mount base tensioner and base tensioner spacer onto base. Turn tensioner bolt to tension timing belt.

**Figure:**
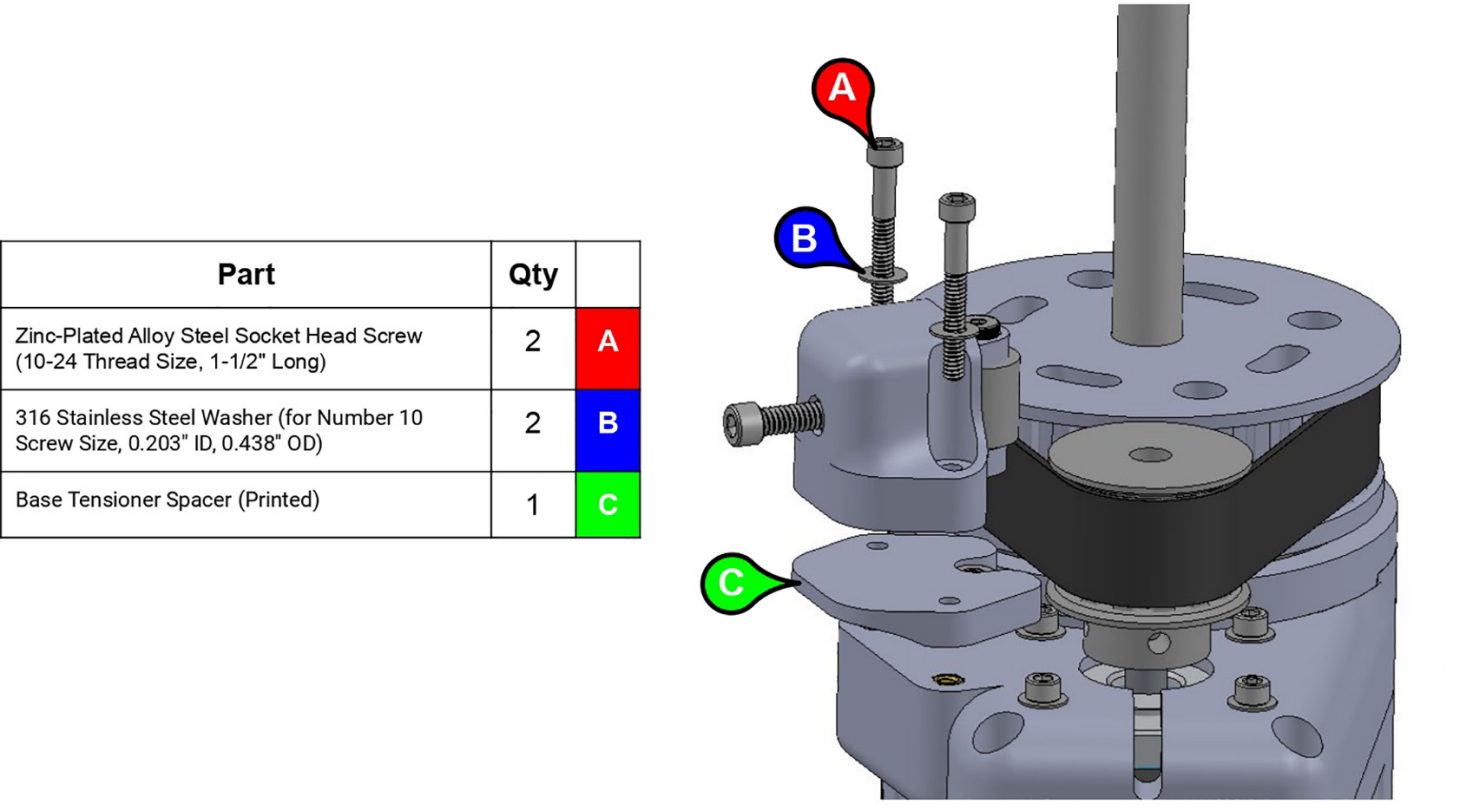

**Step 26:** Fasten central link base plates and central strut to base timing pulley. Secure central link to central axle with nut and washer.

**Figure:**
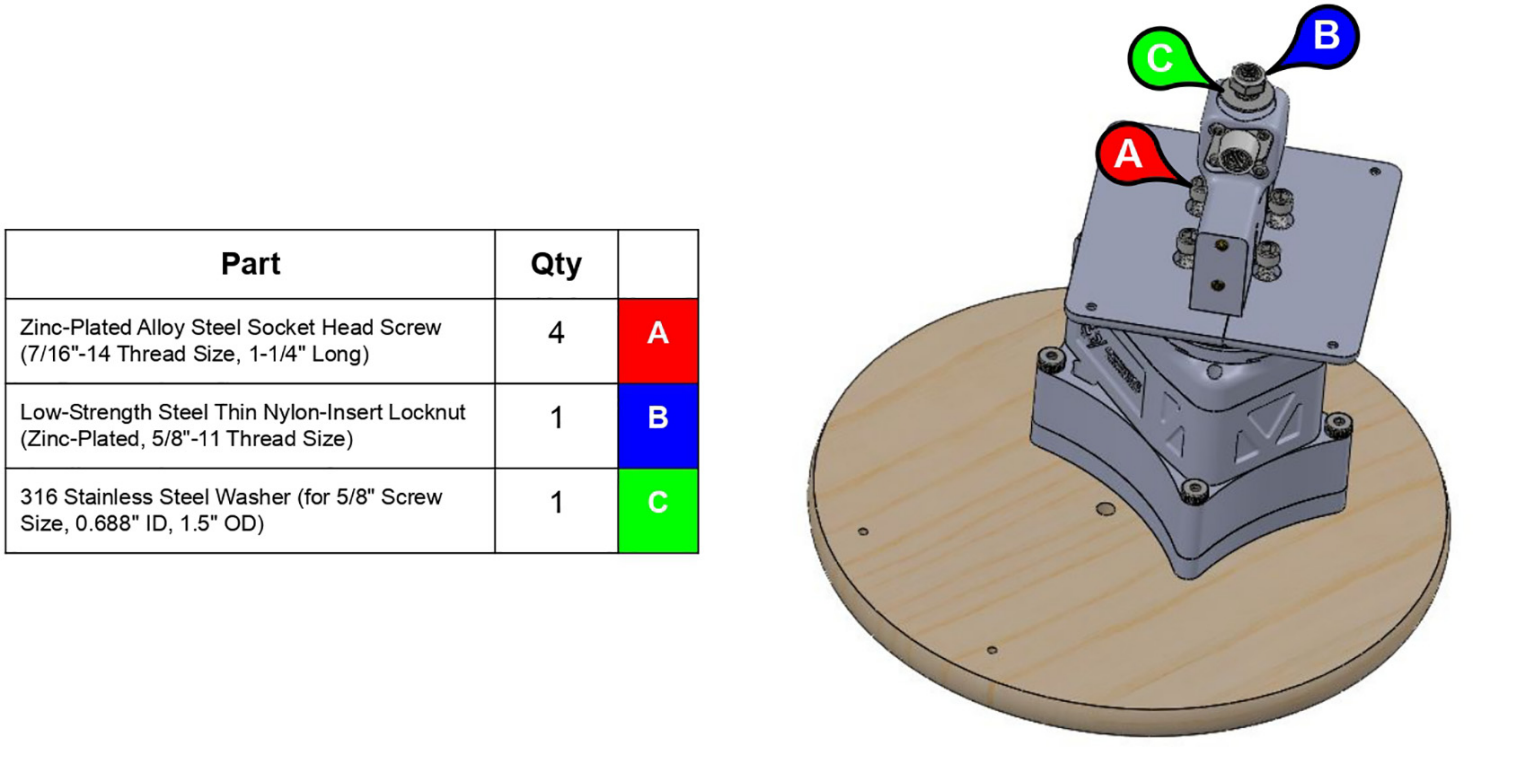

**Step 27:** Use soldering iron to set heat-set inserts into compound elbow timing pulley.

**Figure:**
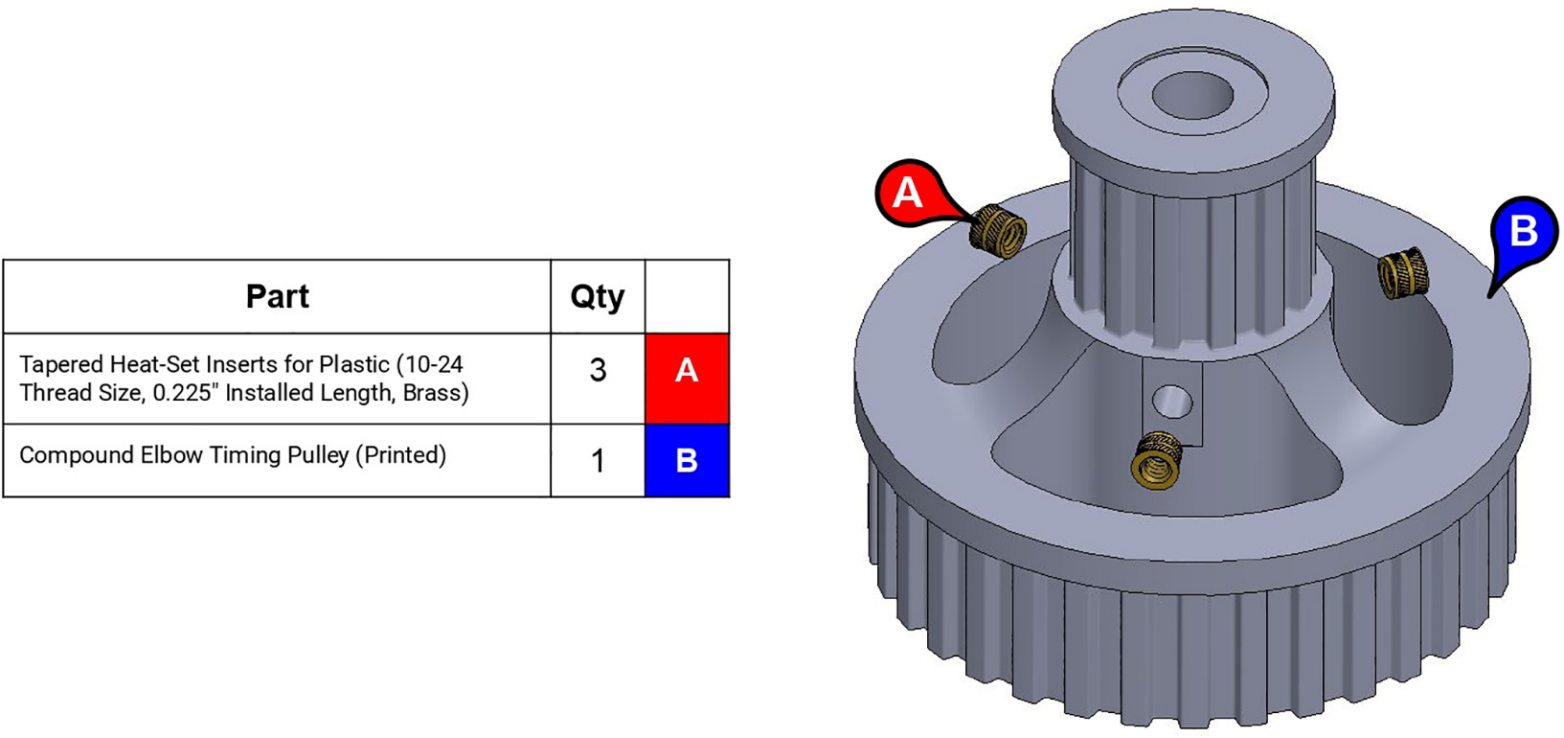

**Step 28:** Use soldering iron to set heat-set inserts into 34T shoulder timing pulley.

**Figure:**
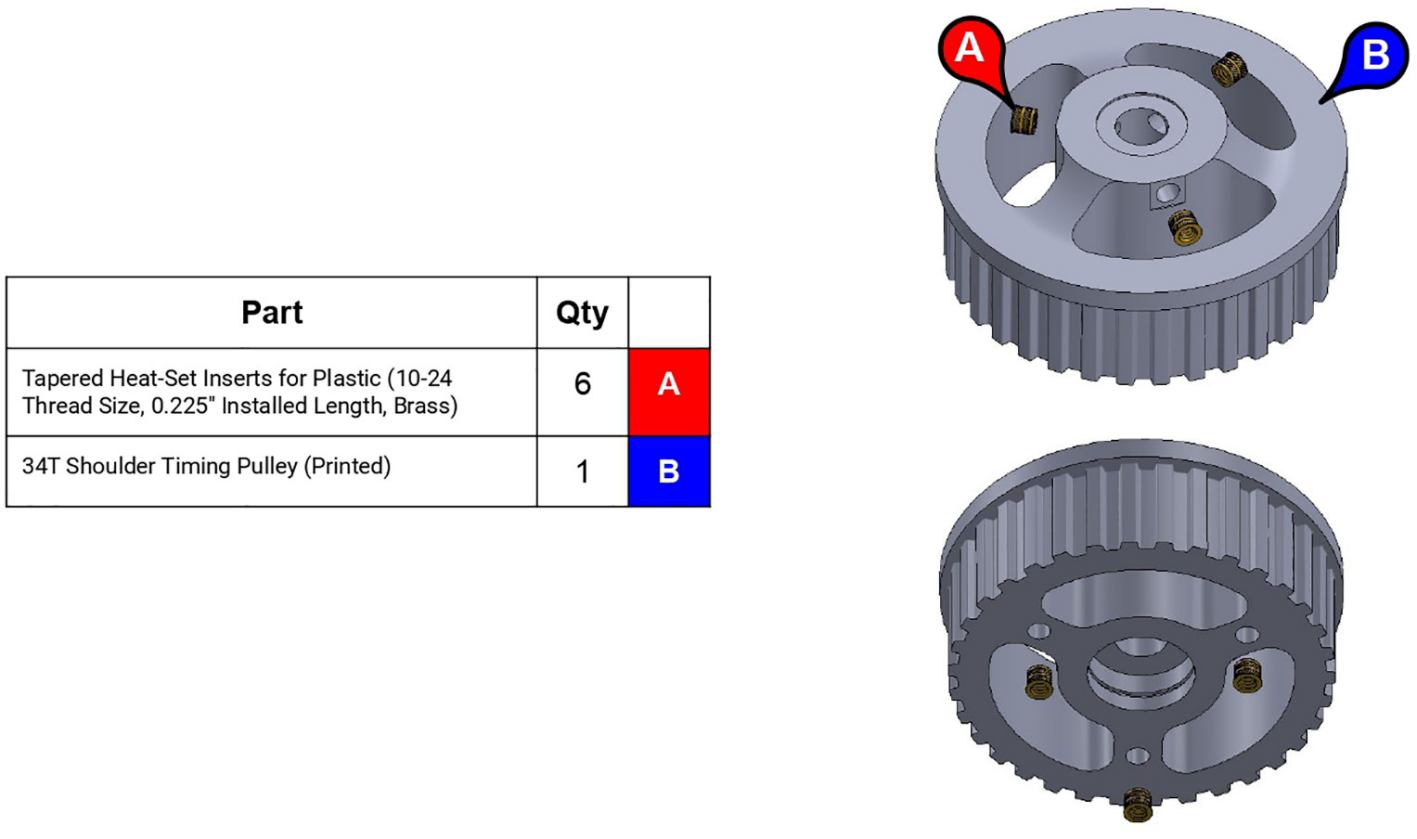

**Step 29:** Press-fit ball bearing into 34T shoulder timing pulley and snap retaining ring into adjacent groove.

**Figure:**
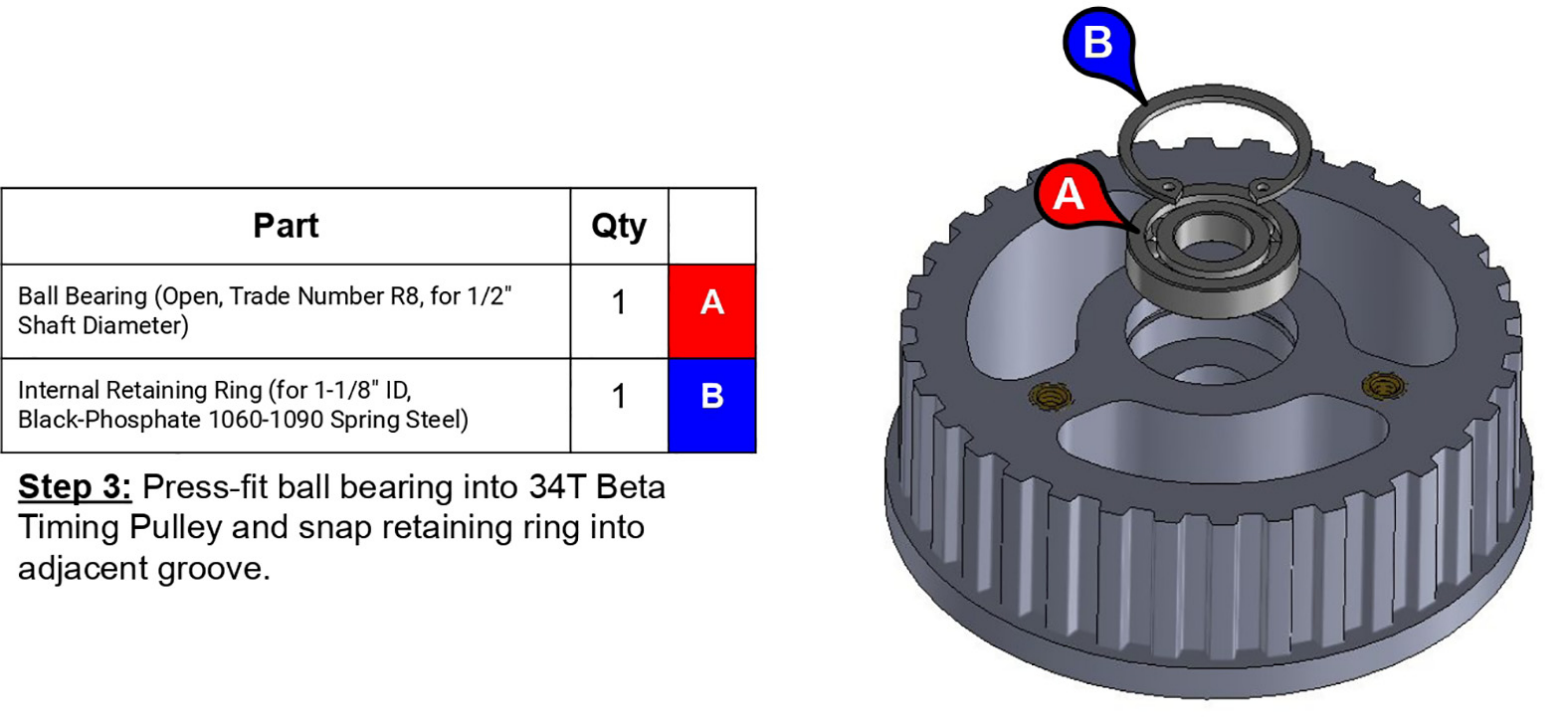

**Step 30:** Use soldering iron to set heat-set inserts into bicep link inboard cap.

**Figure:**
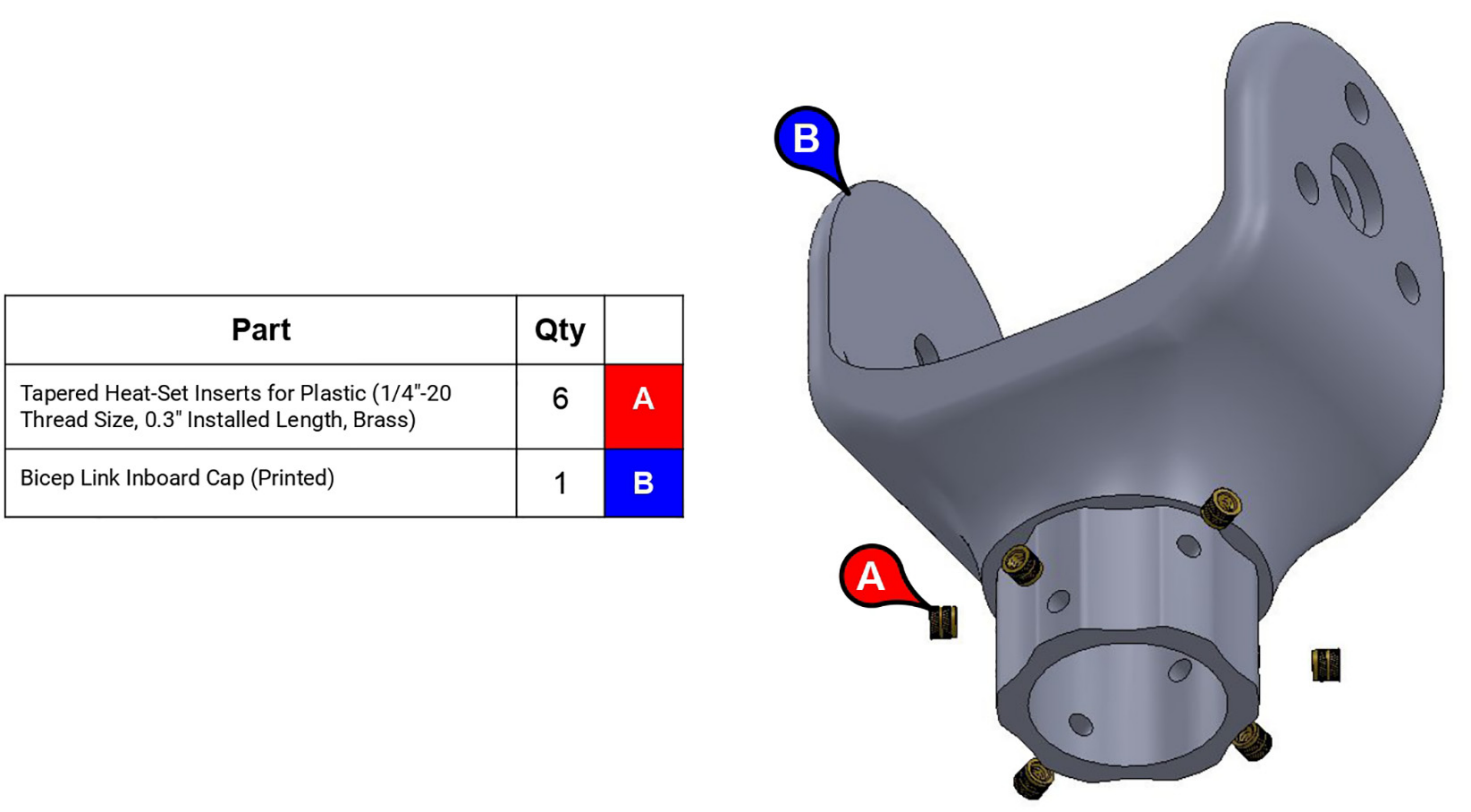

**Step 31:** Press-fit ball bearings into bicep link inboard cap.

**Figure:**
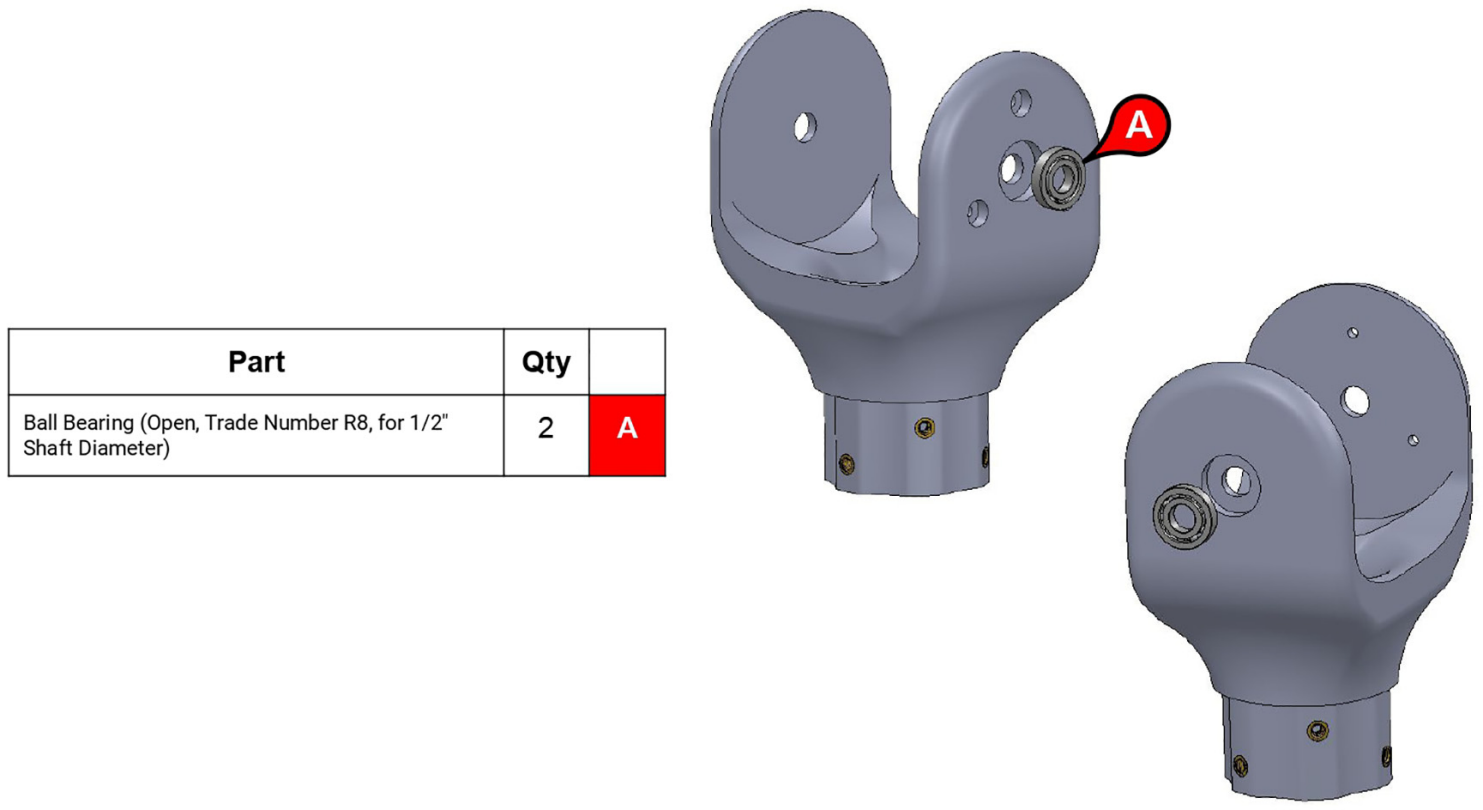

**Step 32:** Fasten 34T shoulder timing pulley to bicep link inboard cap. Ensure that L series timing belt rests between pulley flange and inboard cap.

**Figure:**
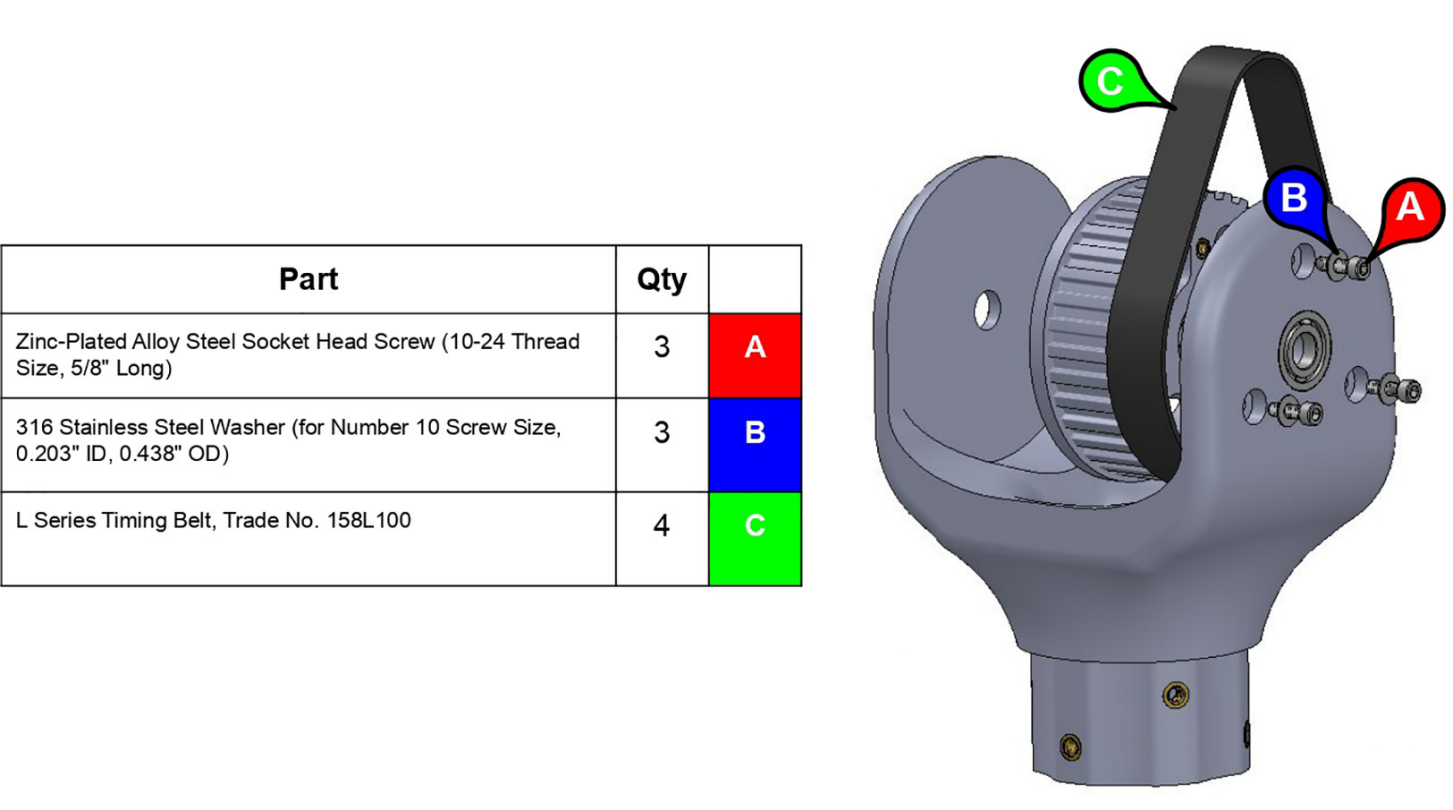

**Step 33:** Pass keyed rotary shaft through bicep link inboard cap, compound elbow timing pulley, and indicated components. Take care to assemble with components in correct order.

**Figure:**
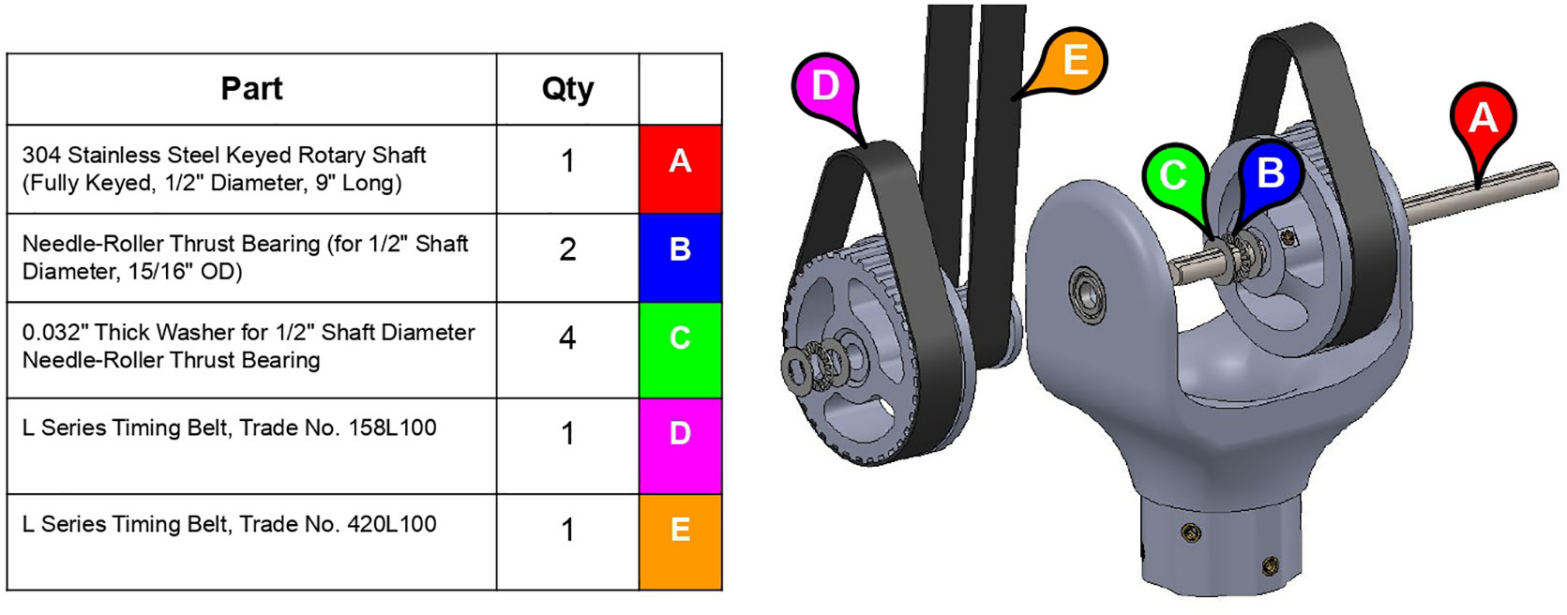

**Step 34:** Fix compound elbow timing pulley to rotary shaft with set screws and loop 42T timing belt through bicep link inboard cap.

**Figure:**
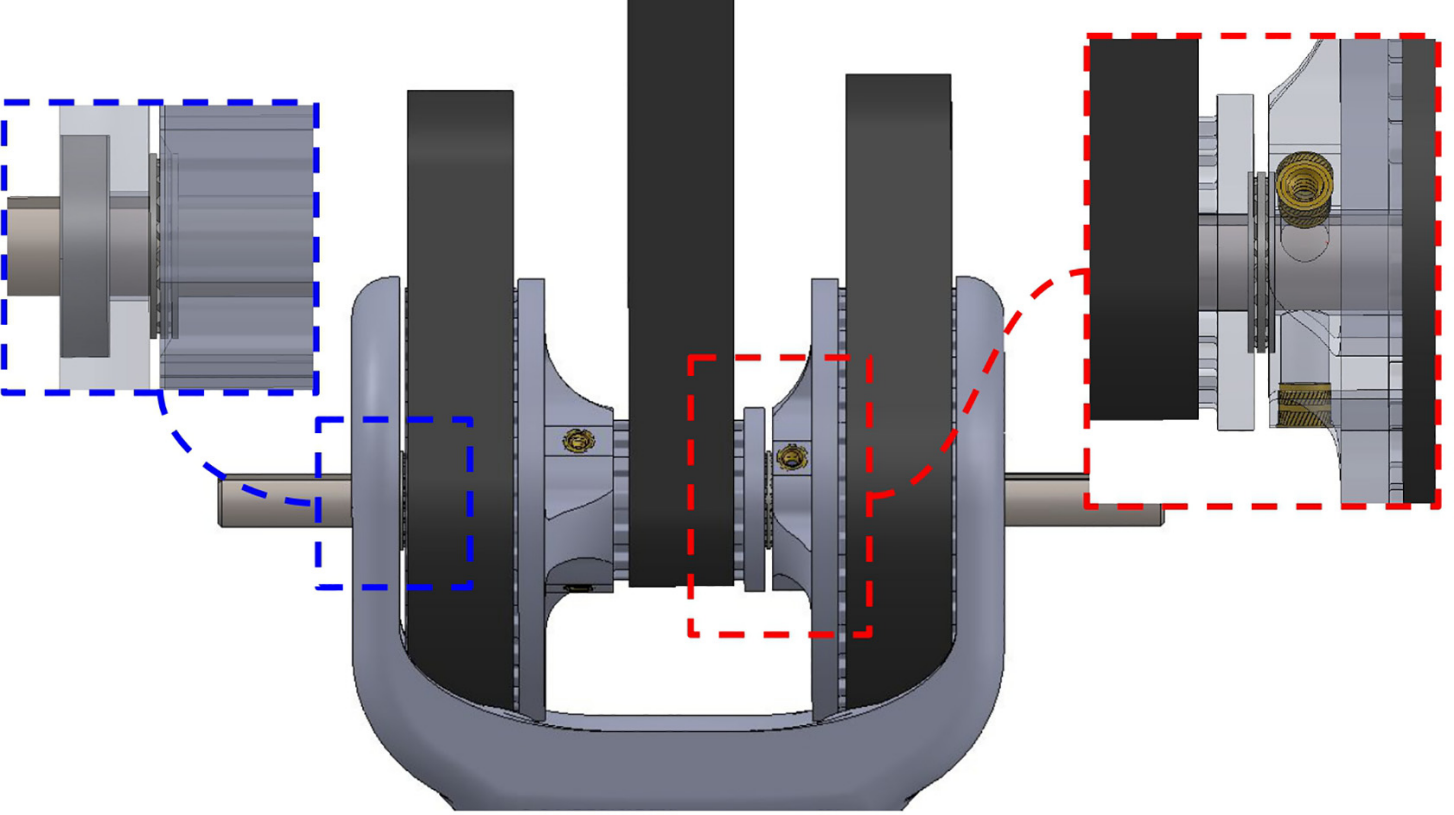

**Step 35:** Pass rotary shaft through central link side assemblies.

**Figure:**
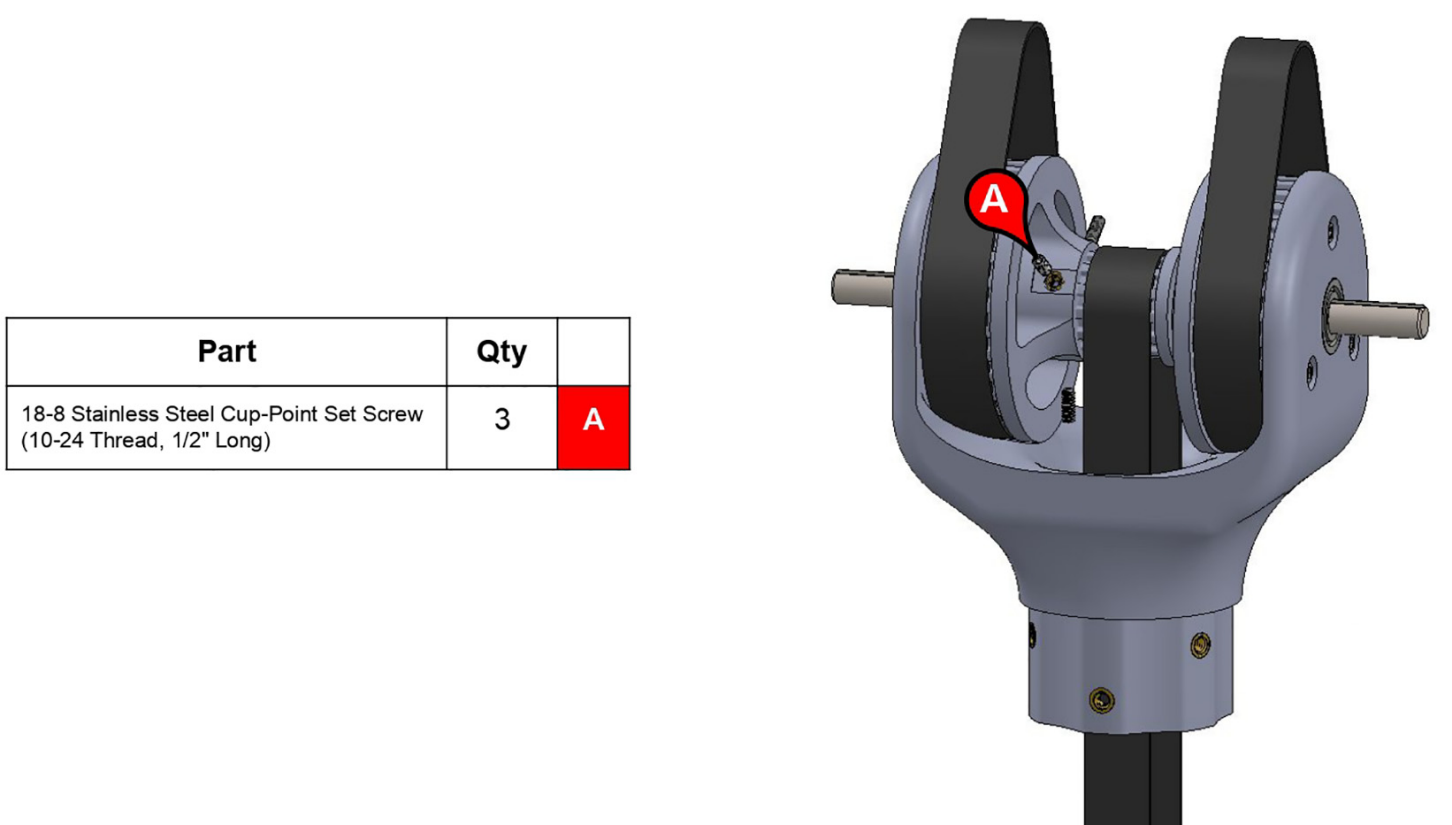

**Step 36:** Fasten central link side assemblies to central link base plates and central strut.

**Figure:**
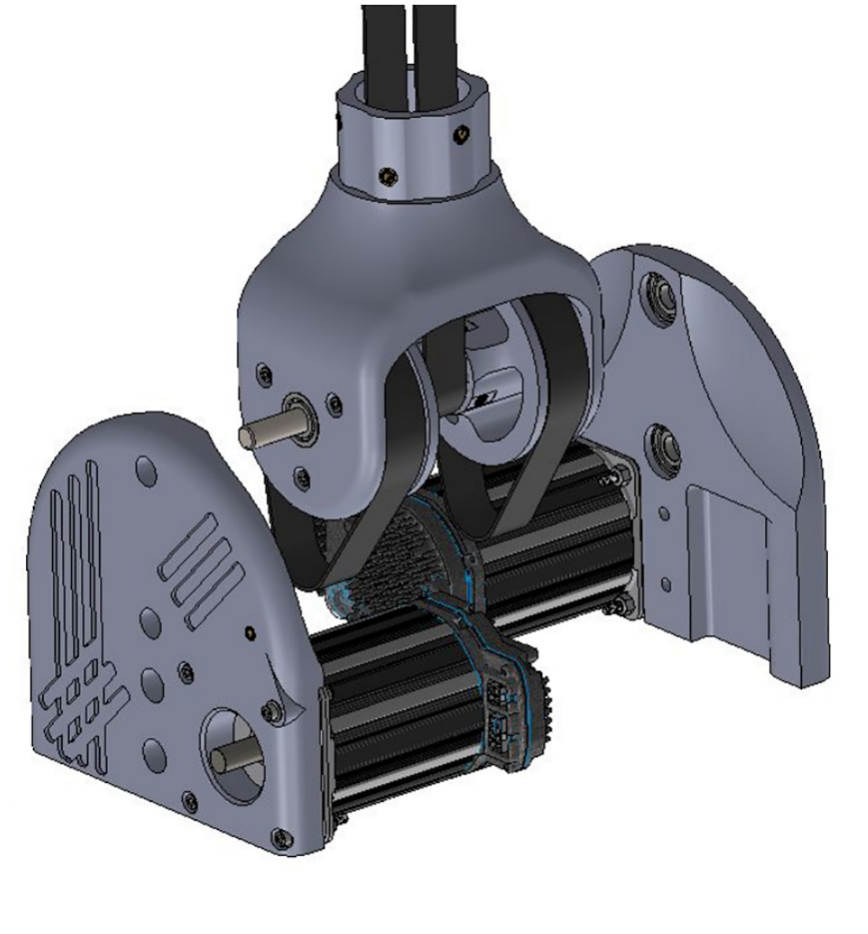

**Step 37:** Engage 13T steel timing pulleys with timing belts and pass keyed rotary shafts through central link ball bearings, pulleys, and roller bearings. Fix 13T pulleys to rotary shafts with set screws.

**Figure:**
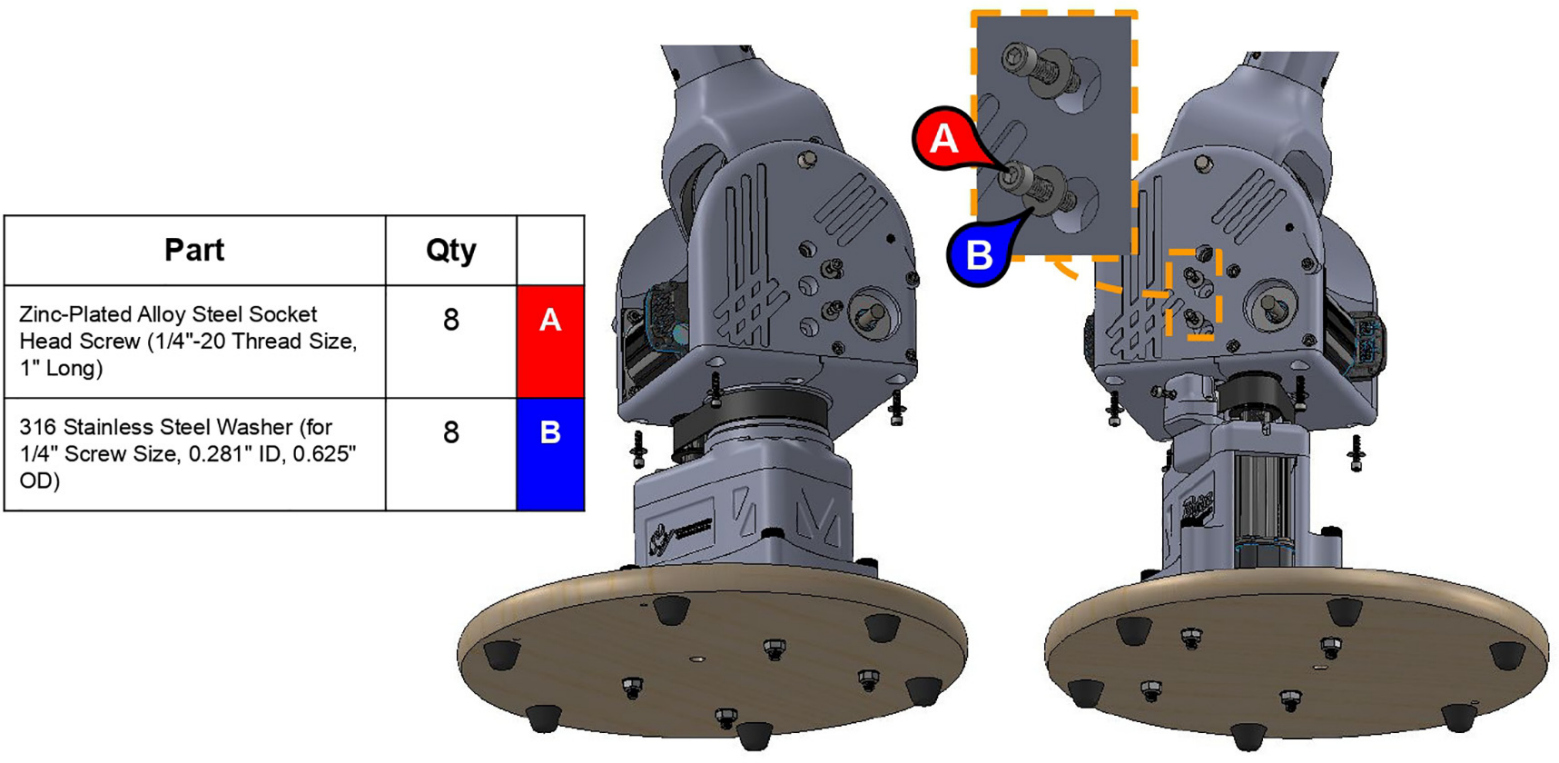

**Step 38:** Use soldering iron to set heat-set inserts into 34T central link timing pulley.

**Figure:**
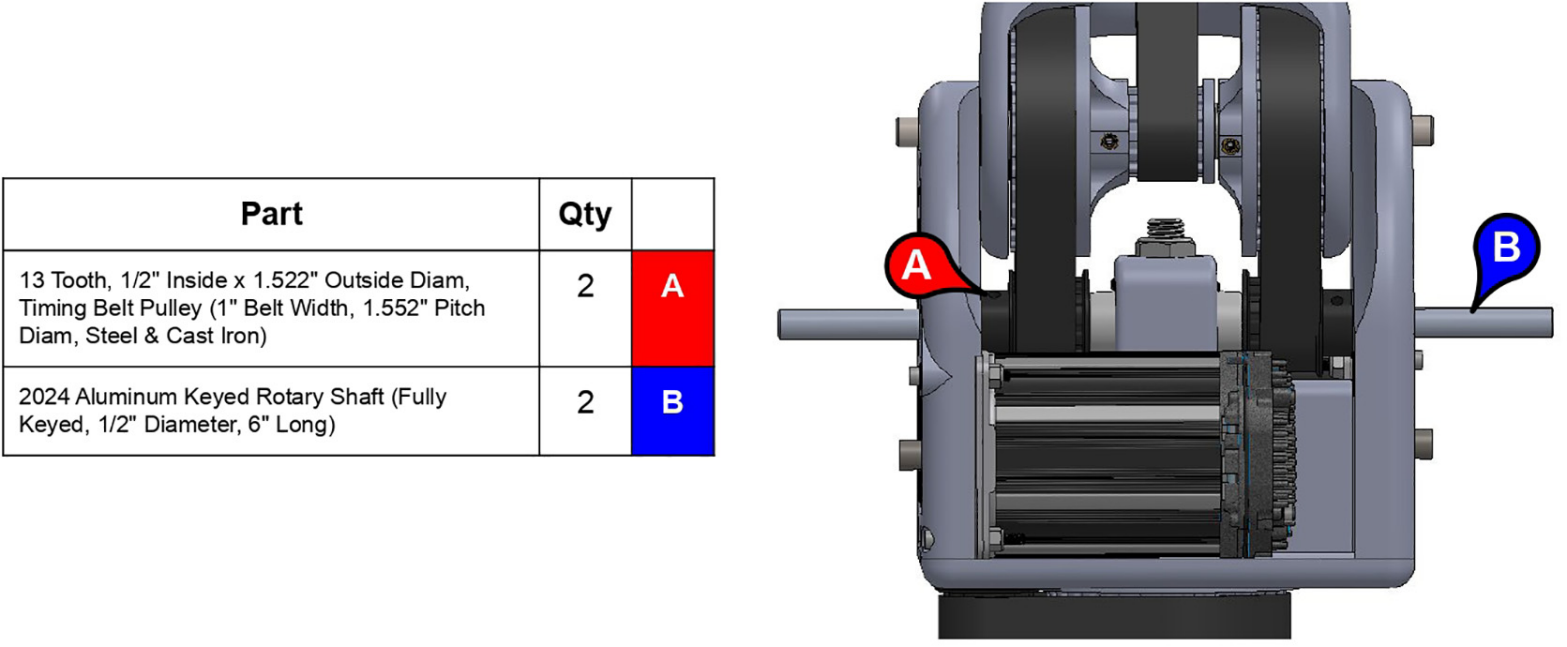

**Step 39:** Slide 34T central link timing pulley, 13T steel timing pulley, and timing belt onto each side of the central link. Use set screws to fix 13T steel timing pulleys to motor shafts.

**Figure:**
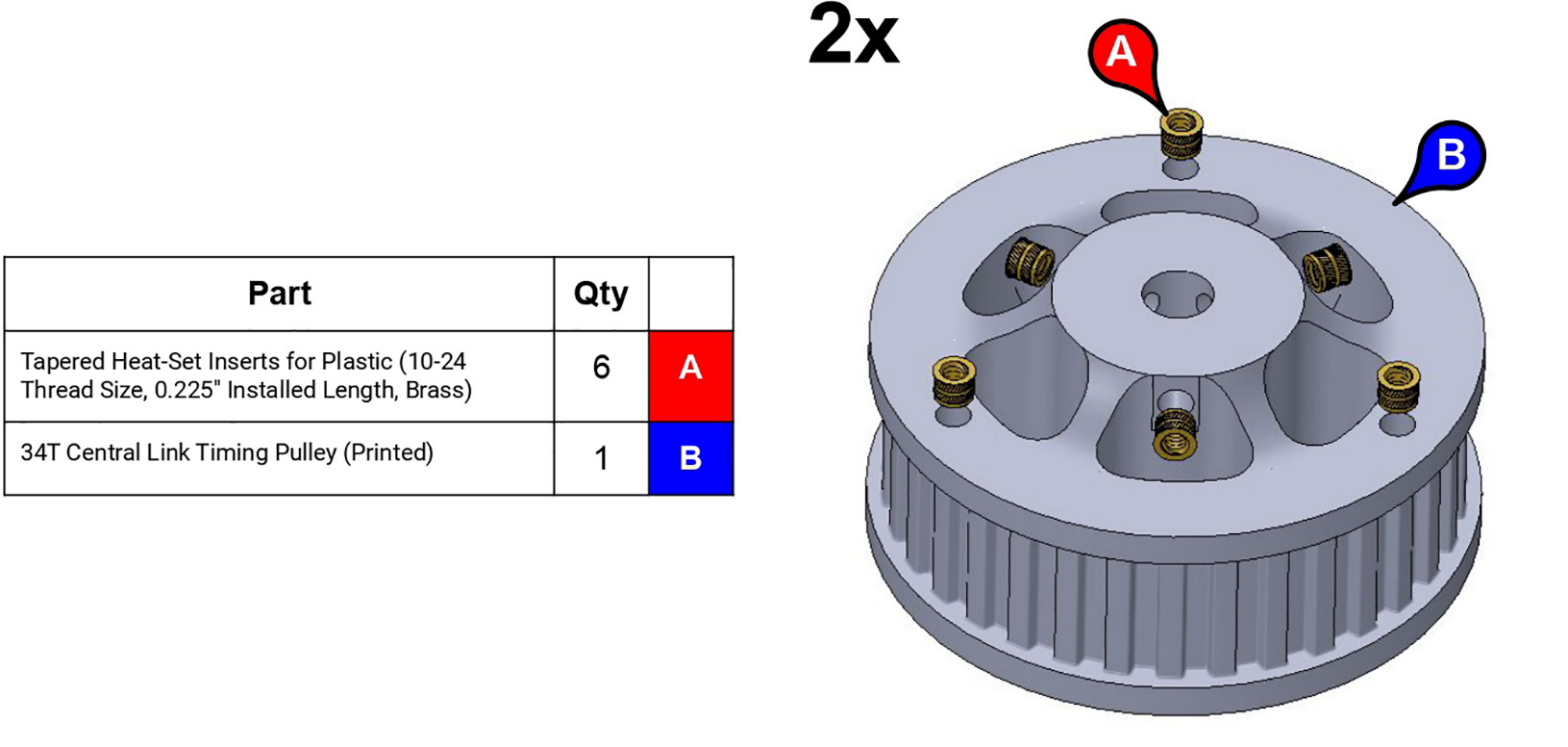

**Step 40:** Use set screws to fix 34T central link timing pulleys to rotary shafts.

**Figure:**
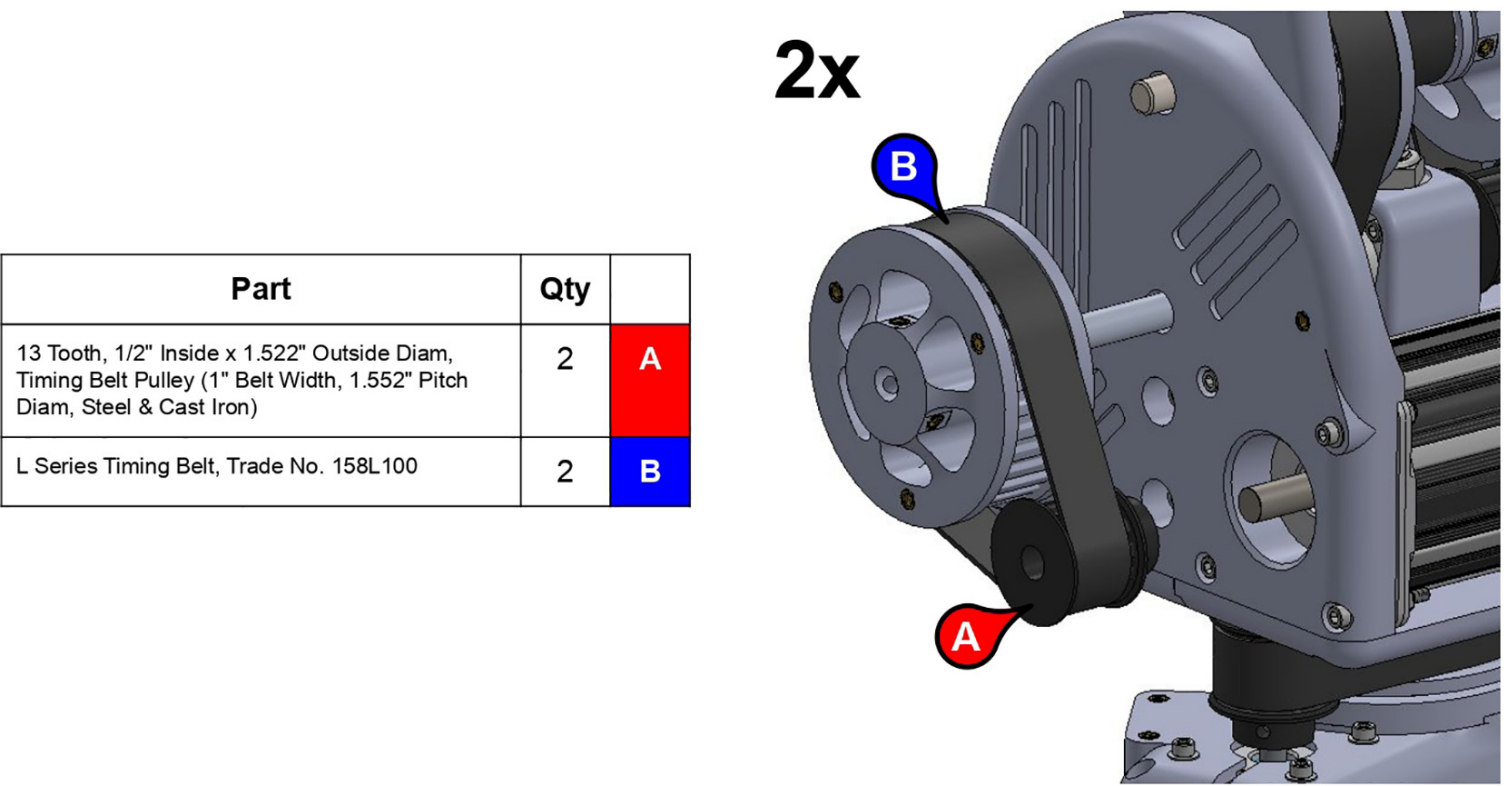

**Step 41:** Fasten timing pulley caps to 34T central link timing pulleys.

**Figure:**
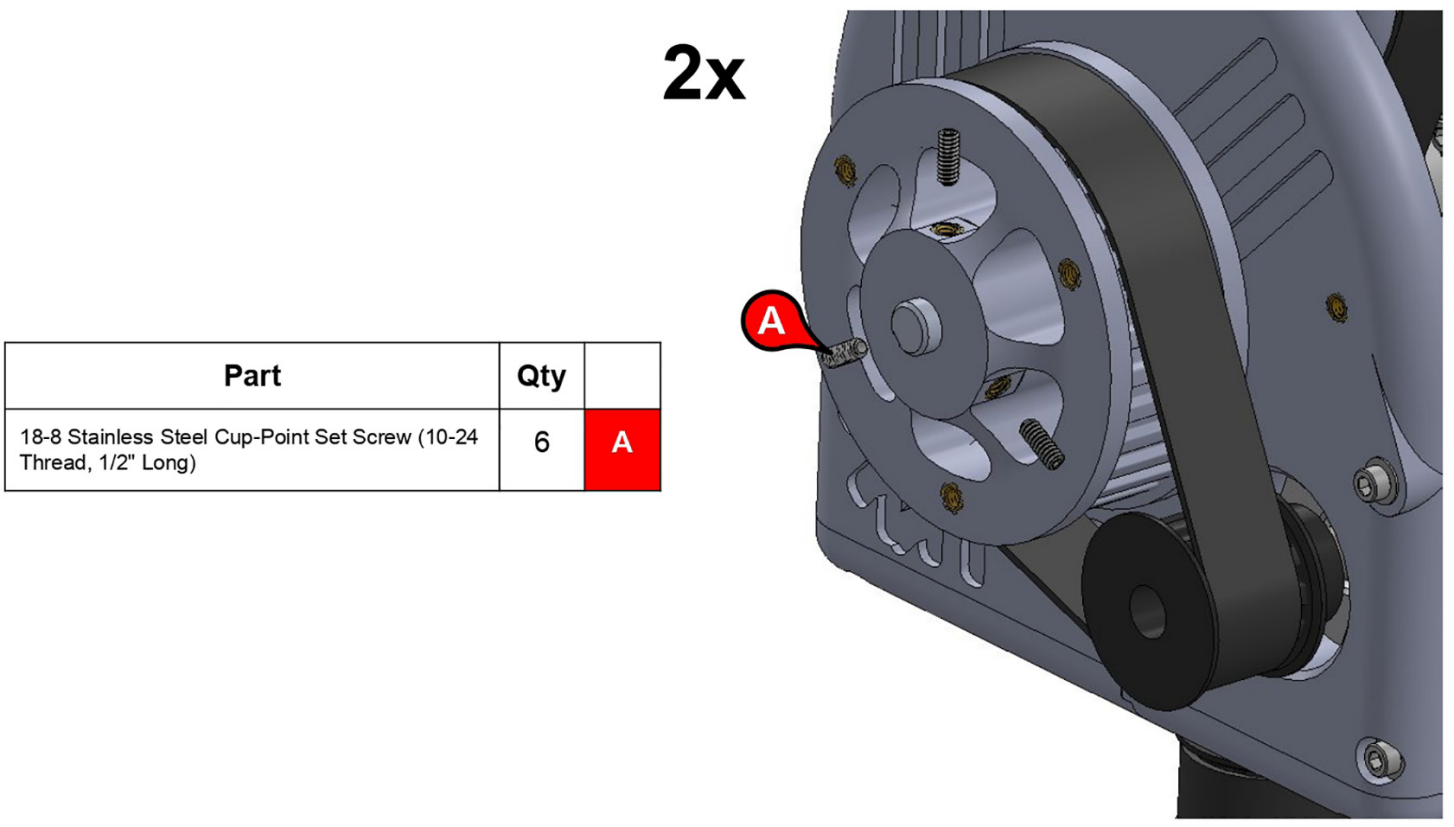

**Step 42:** Use soldering iron to set heat-set inserts into central link tensioner housing.

**Figure:**
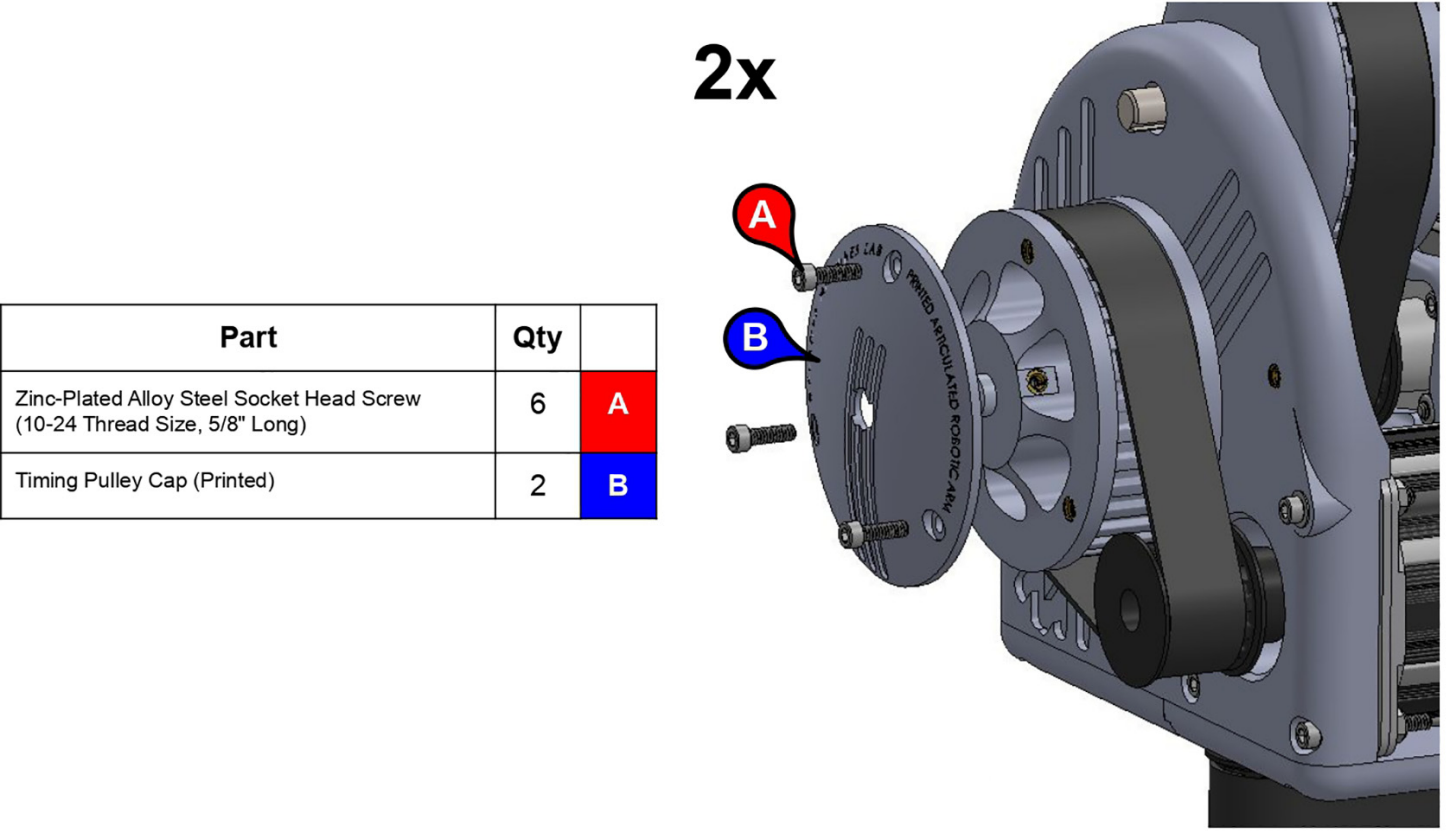

**Step 43:** Assemble various components of central link tensioner.

**Figure:**
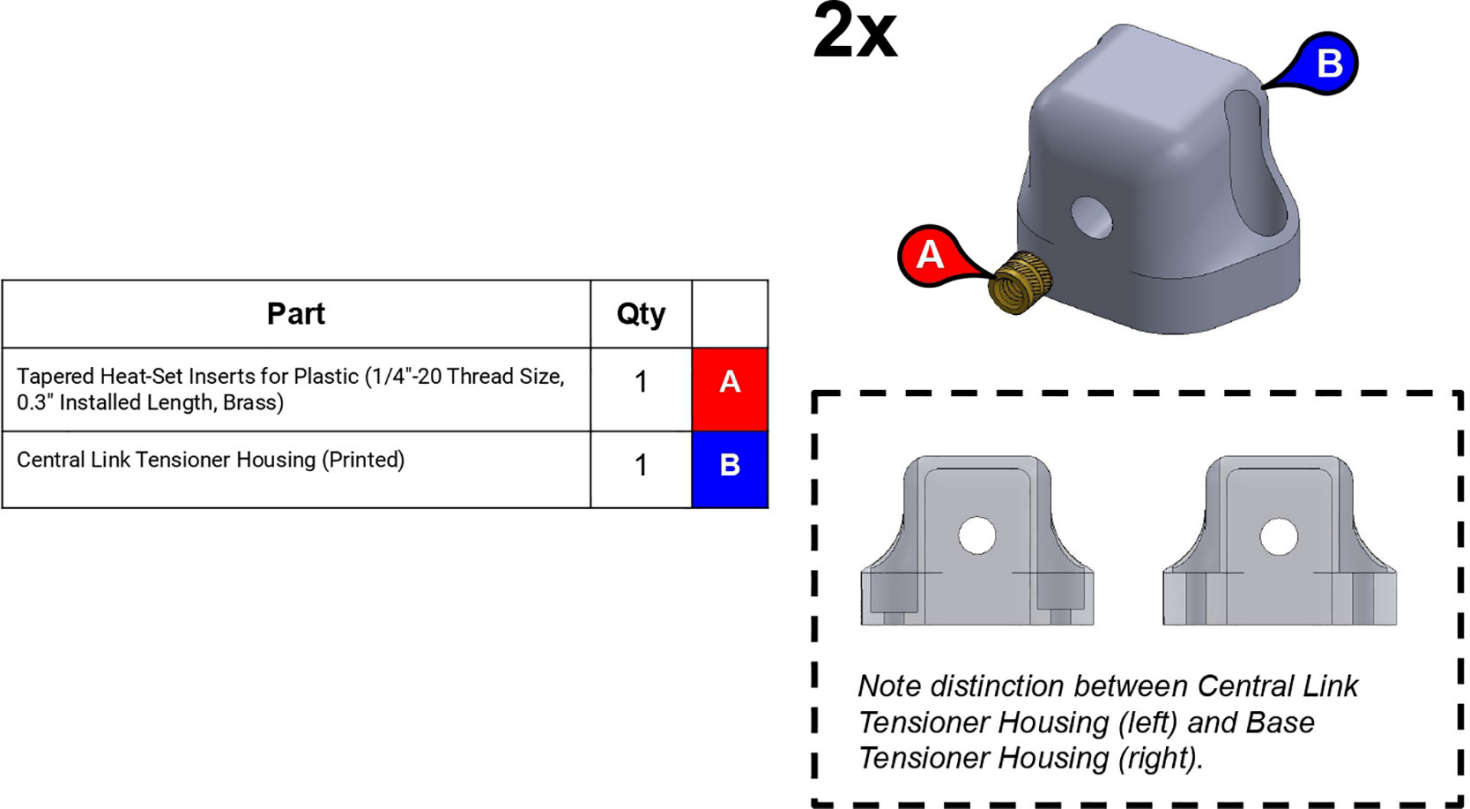

**Step 44:** Mount central link tensioner onto central link. Turn tensioner bolt to tension timing belt.

**Figure:**
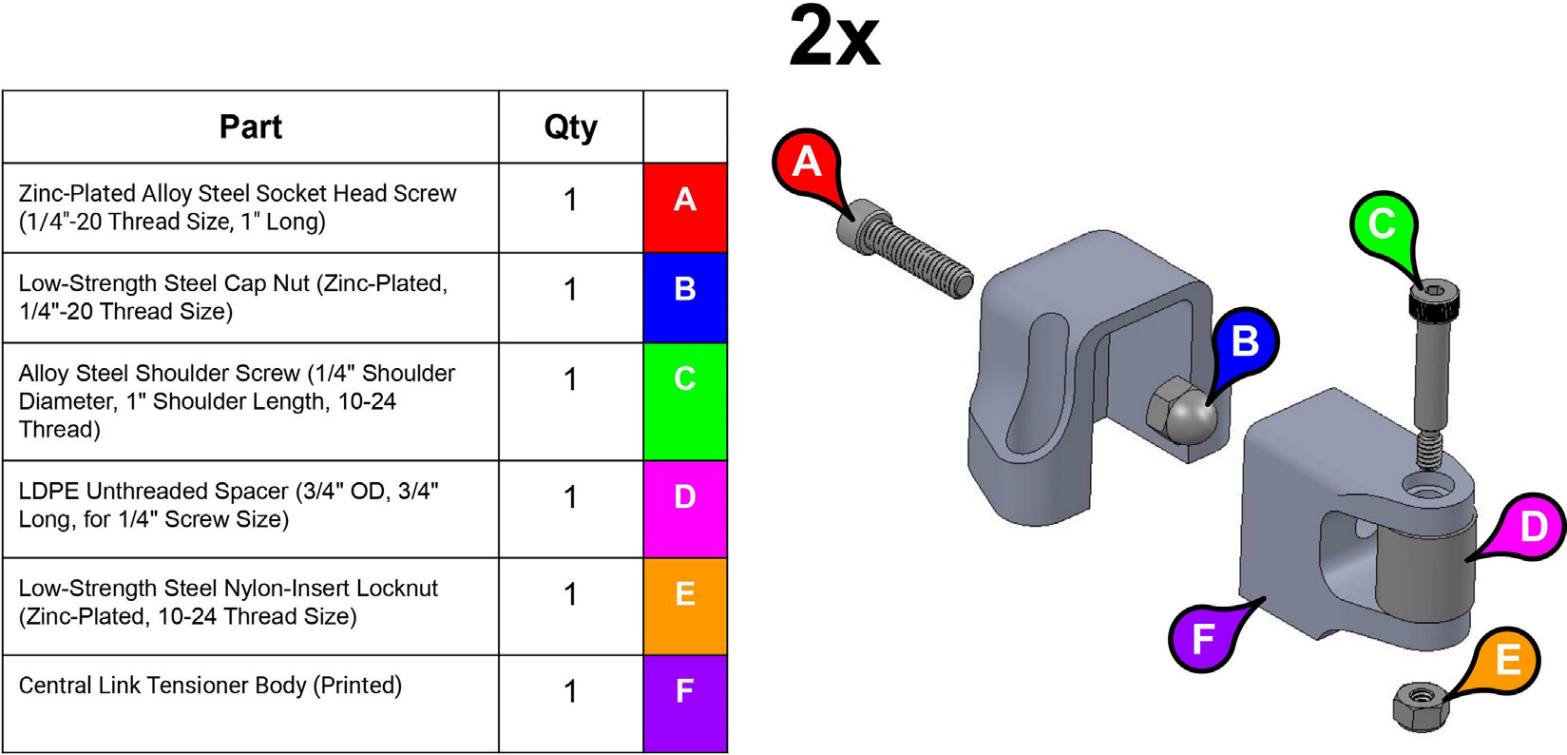

**Step 45:** Saw 3.25” diameter acrylic tube into two 12” segments.

**Figure:**
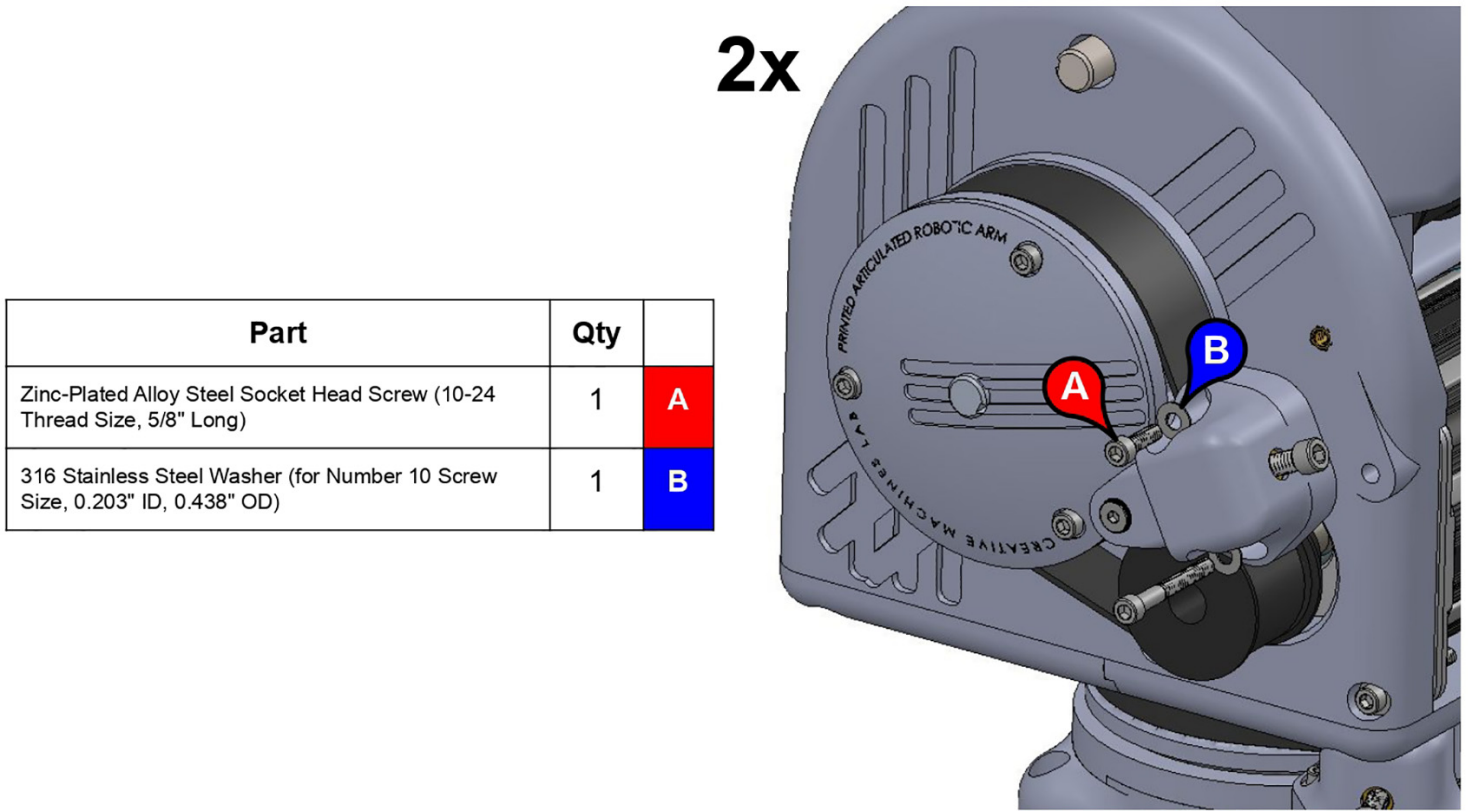

**Step 46:** Align drill jig with edge of acrylic tube and tighten with fasteners.

**Figure:**
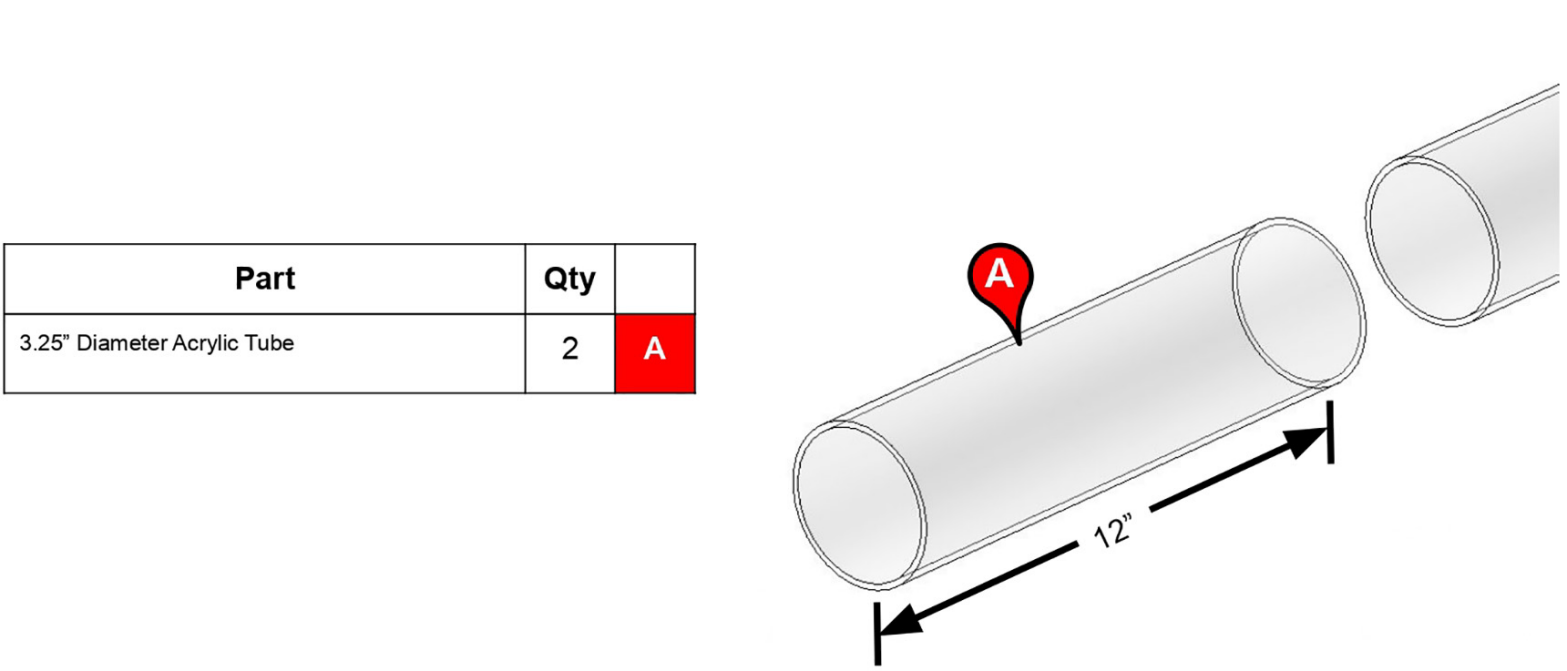

**Step 47:** Use hand drill and.25” drill bit to 6x drill holes in the locations indicated by the drill jig.

**Figure:**
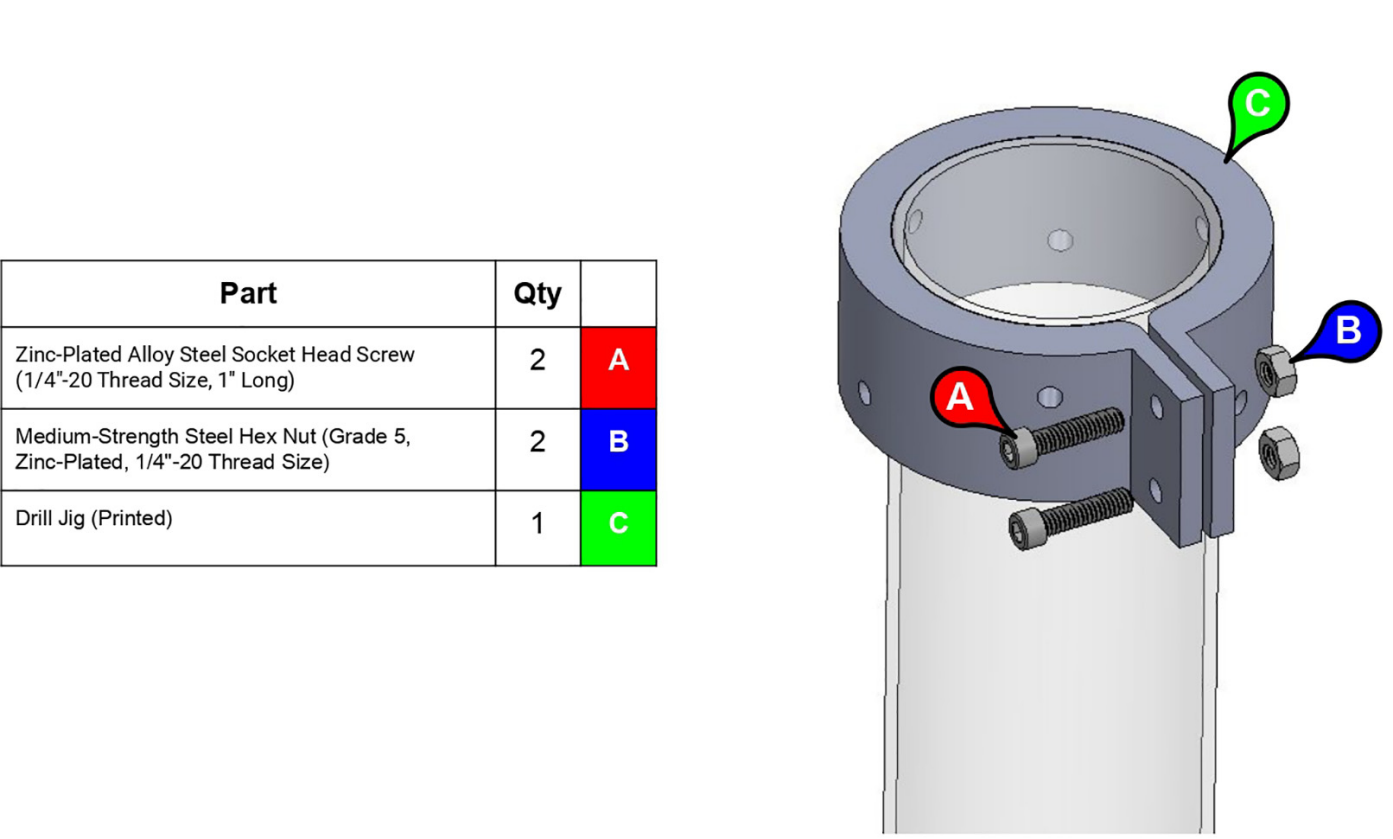

**Step 48:** Slide drill jig to other end of tube and repeat drilling operation. Ensure that holes are properly aligned and in the correct orientation.

**Figure:**
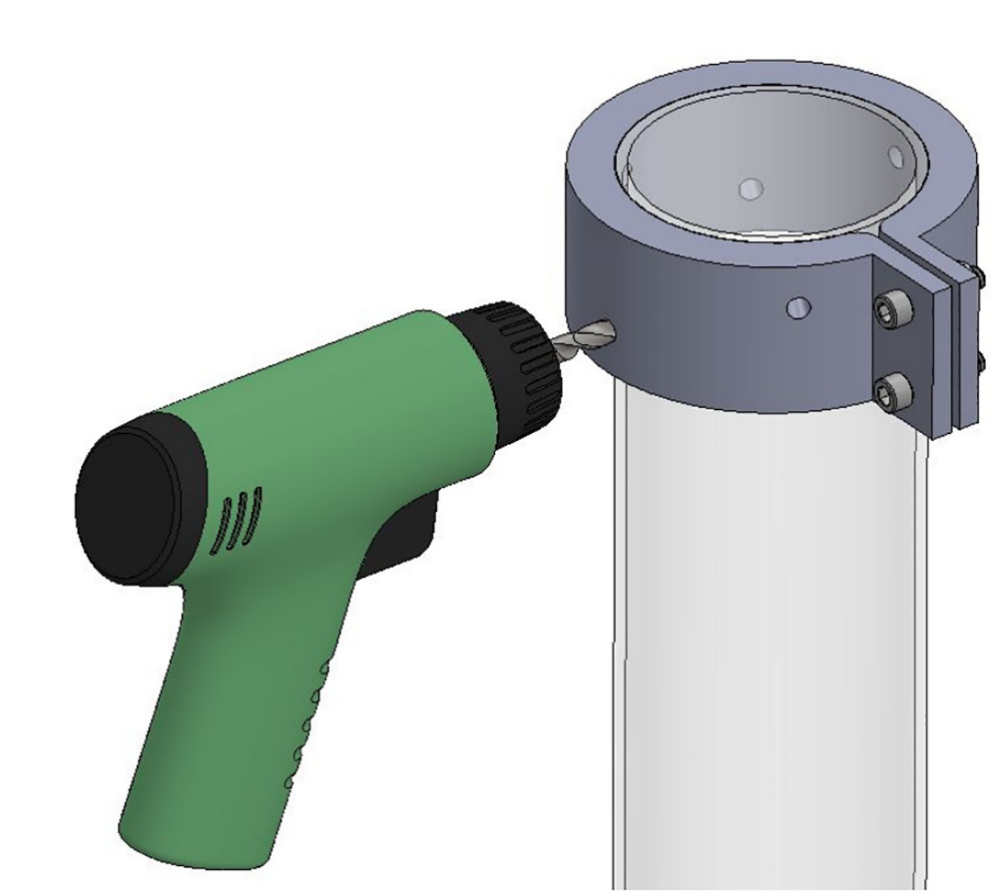

**Step 49:** Repeat hole drilling process for other tube.

**Figure:**
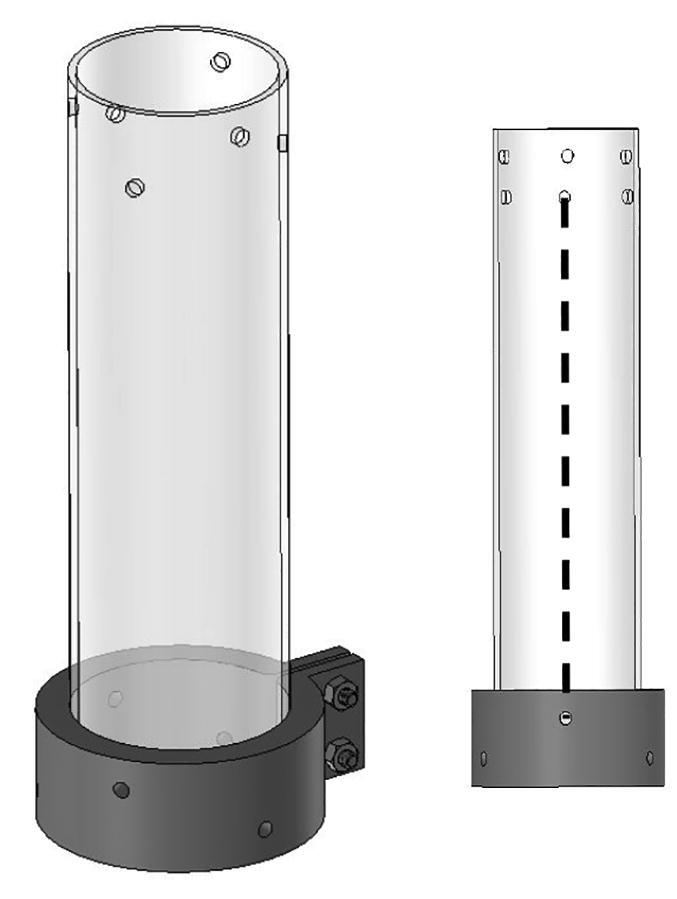

**Step 50:** Use soldering iron to set heat-set inserts into bicep link outboard cap.

**Figure:**
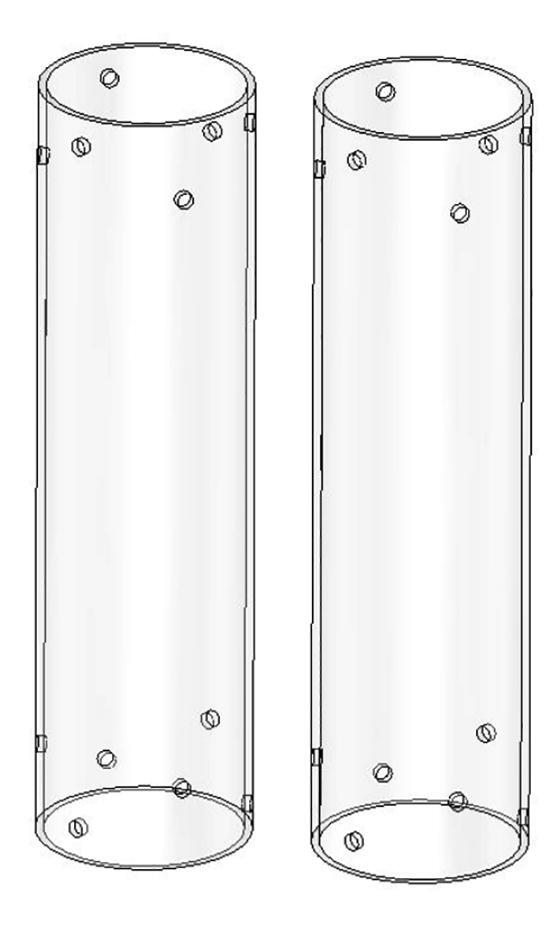

**Step 51:** Use soldering iron to set heat-set inserts into bicep link outboard cap.

**Figure:**
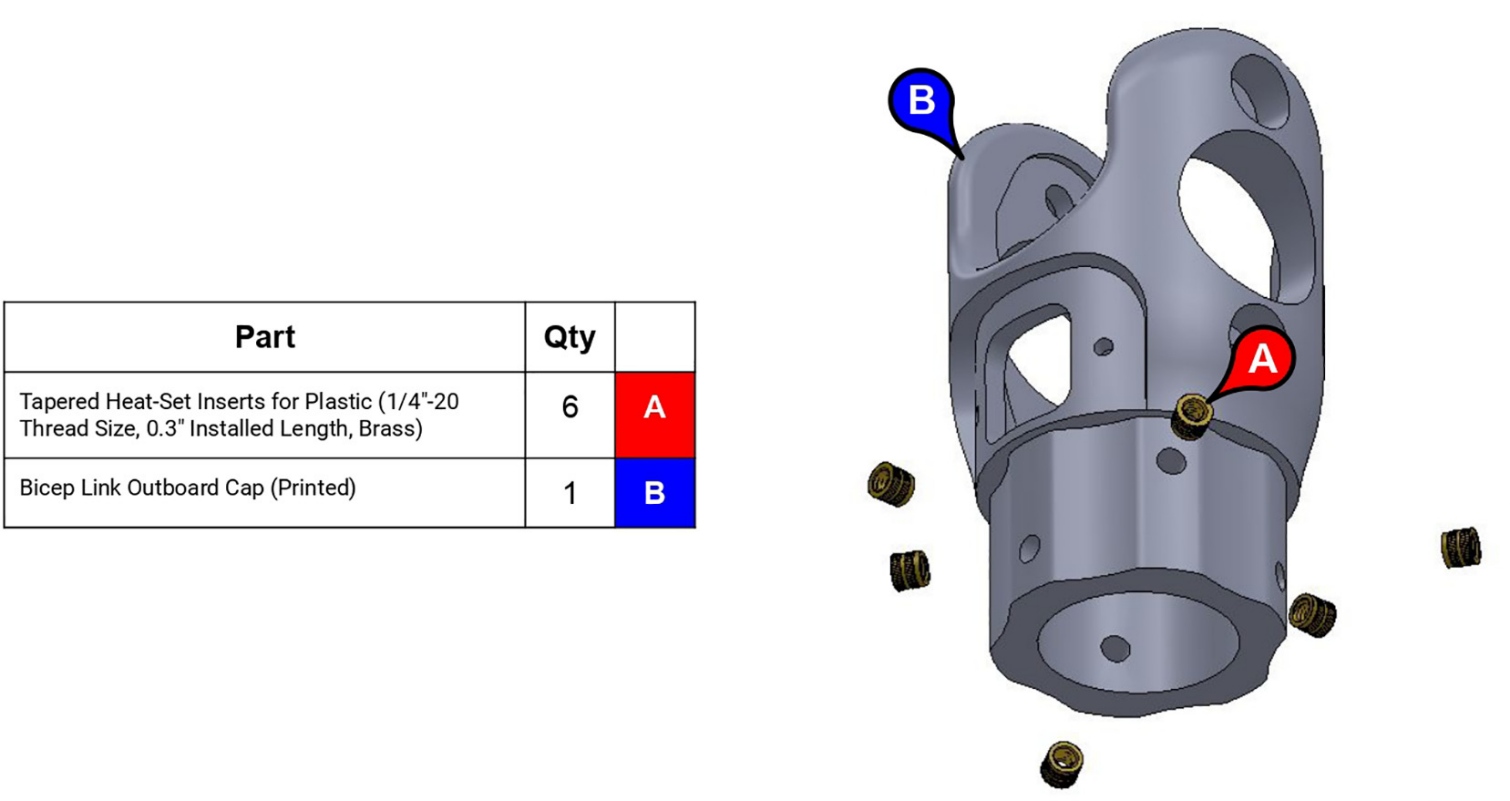

**Step 52:** Use soldering iron to set heat-set inserts into 13T elbow timing pulley.

**Figure:**
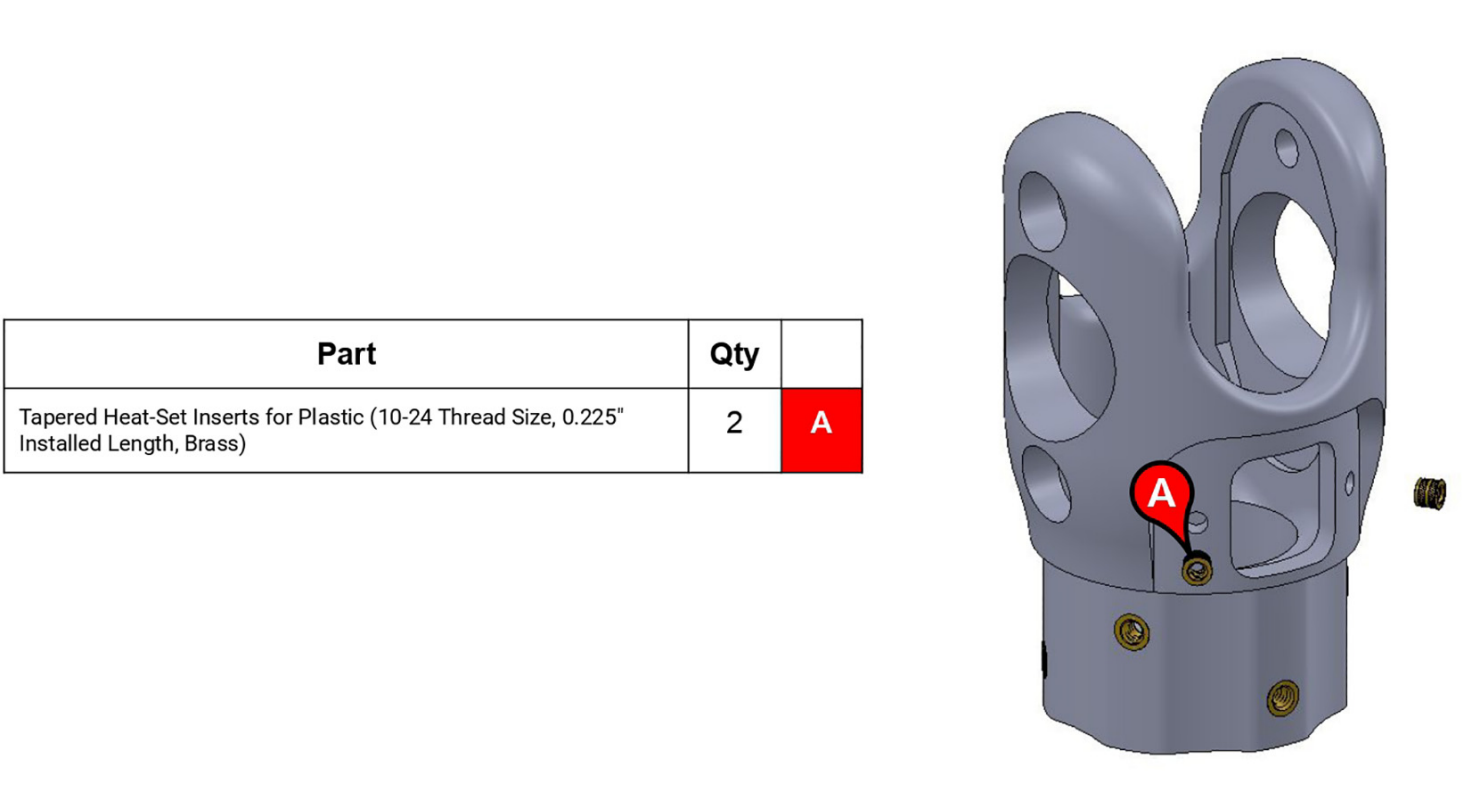

**Step 53:** Use soldering iron to set heat-set inserts into forearm link inboard cap.

**Figure:**
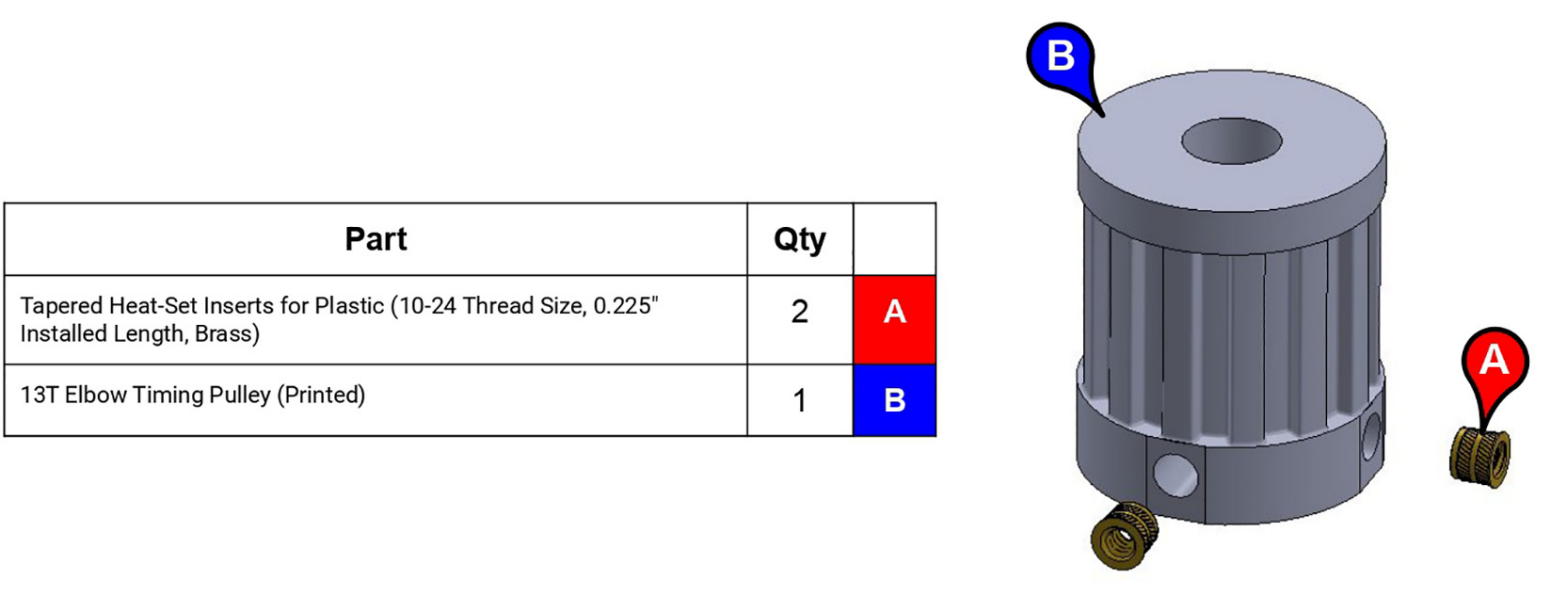

**Step 54:** Use soldering iron to set heat-set inserts into forearm link inboard cap.

**Figure:**
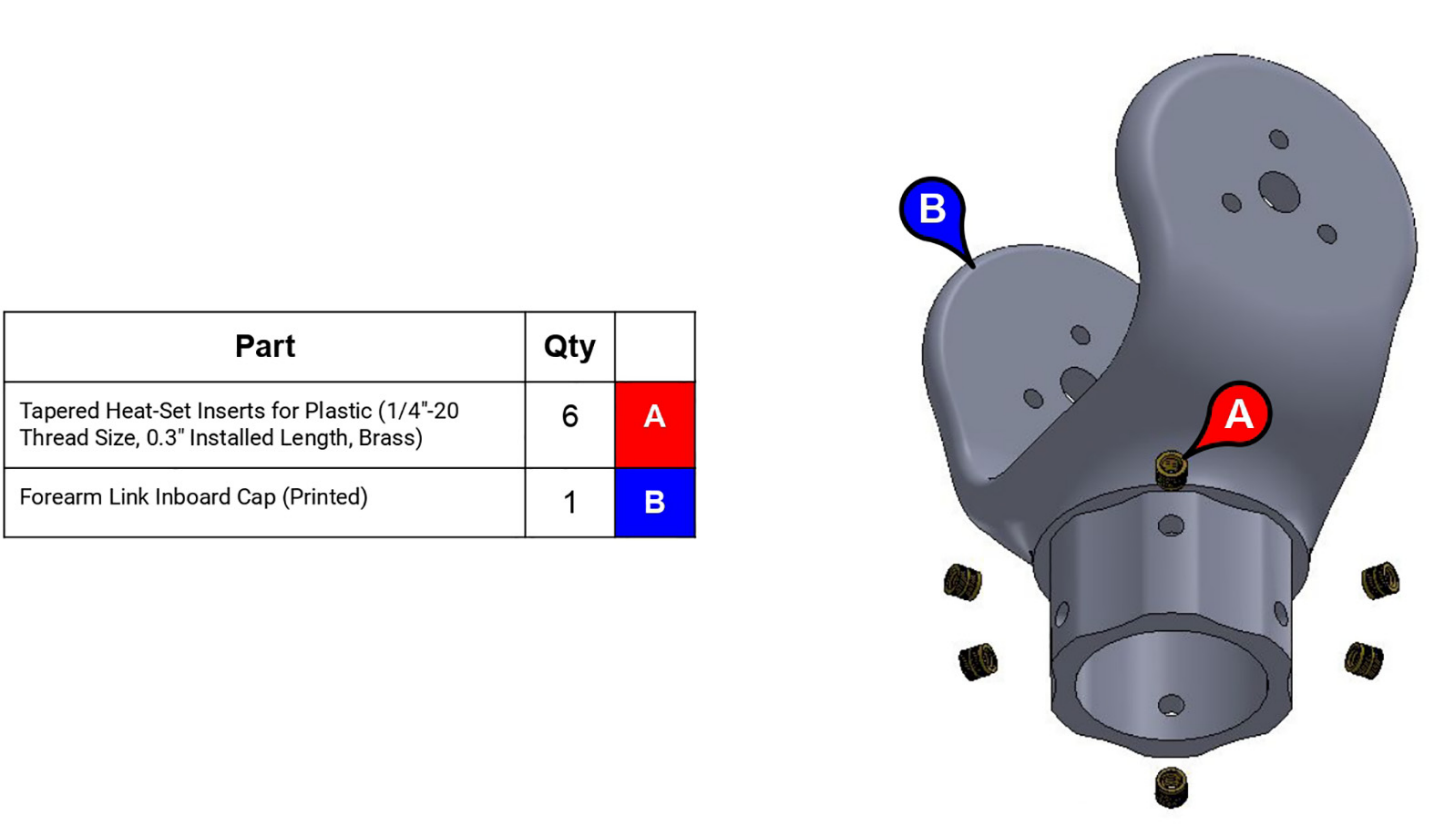

**Step 55:** Use soldering iron to set heat-set inserts into elbow joint couplers.

**Figure:**
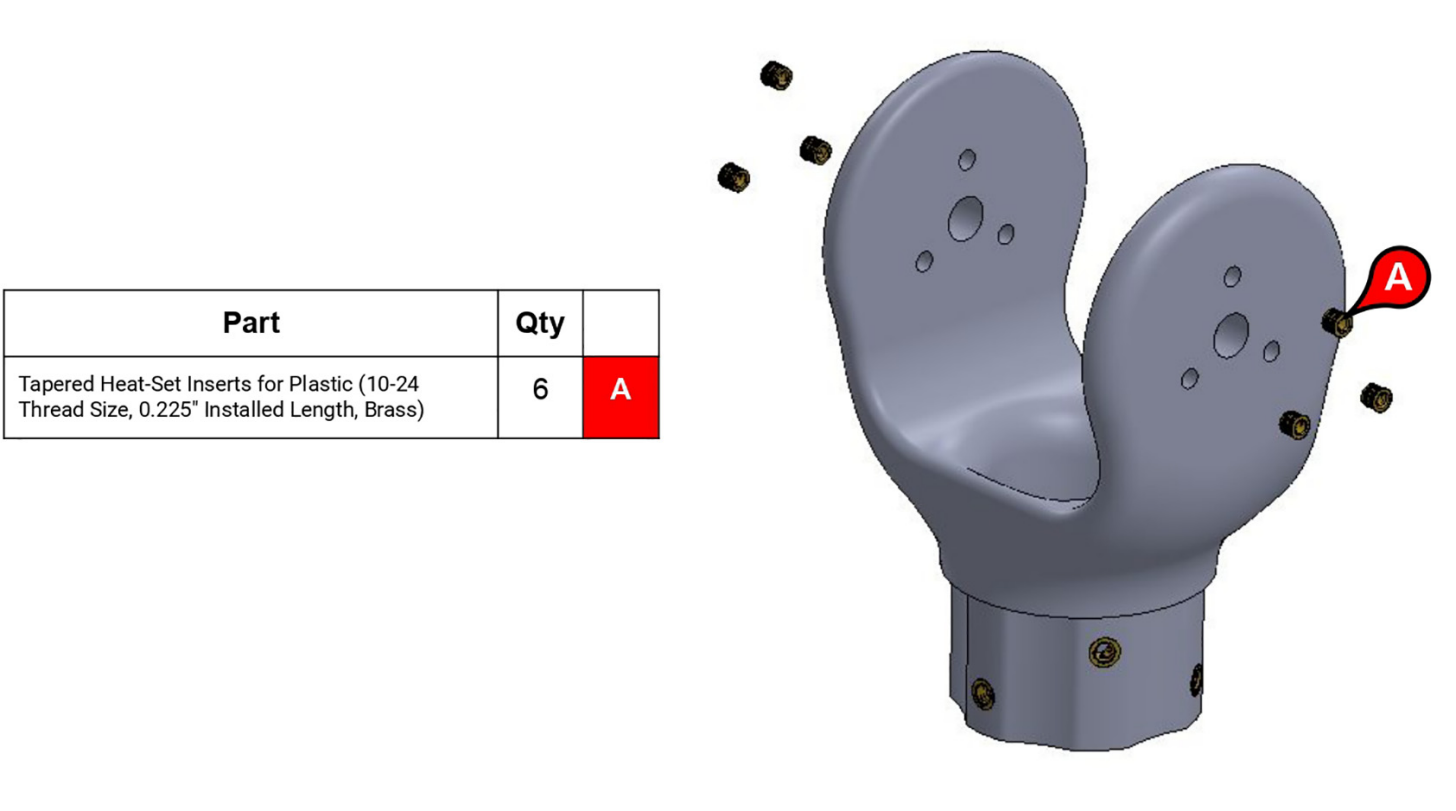

**Step 56:** Partially fasten mounted bearings to bicep link outboard cap.

**Figure:**
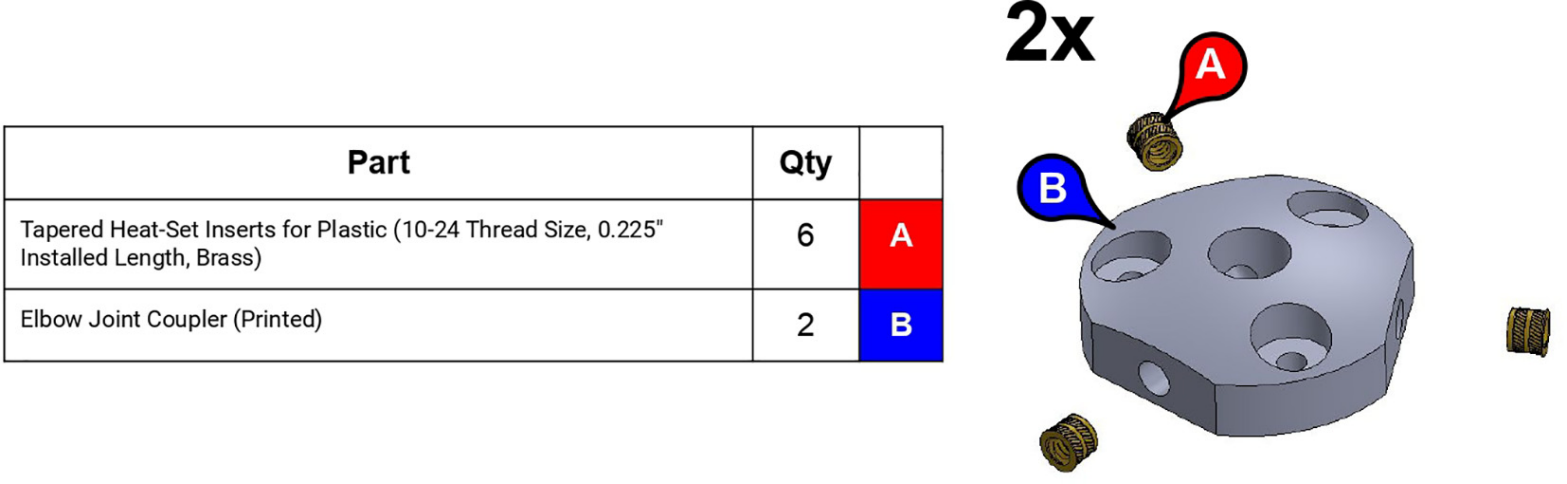

**Step 57:** Slide bicep link outboard cap and acrylic tube onto bicep link inboard cap.

**Figure:**
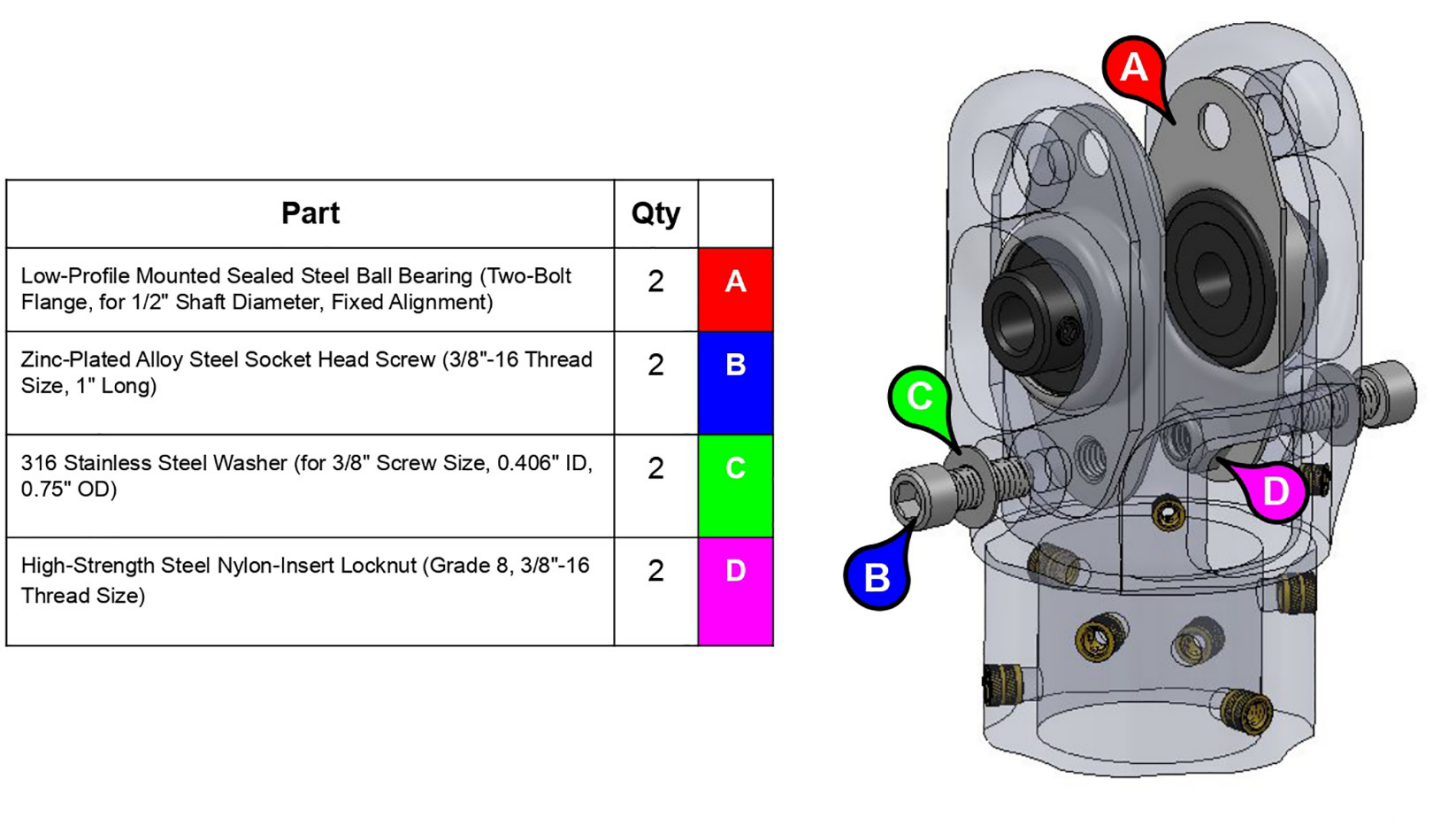

**Step 58:** Fasten acrylic tube to bicep link caps.

**Figure:**
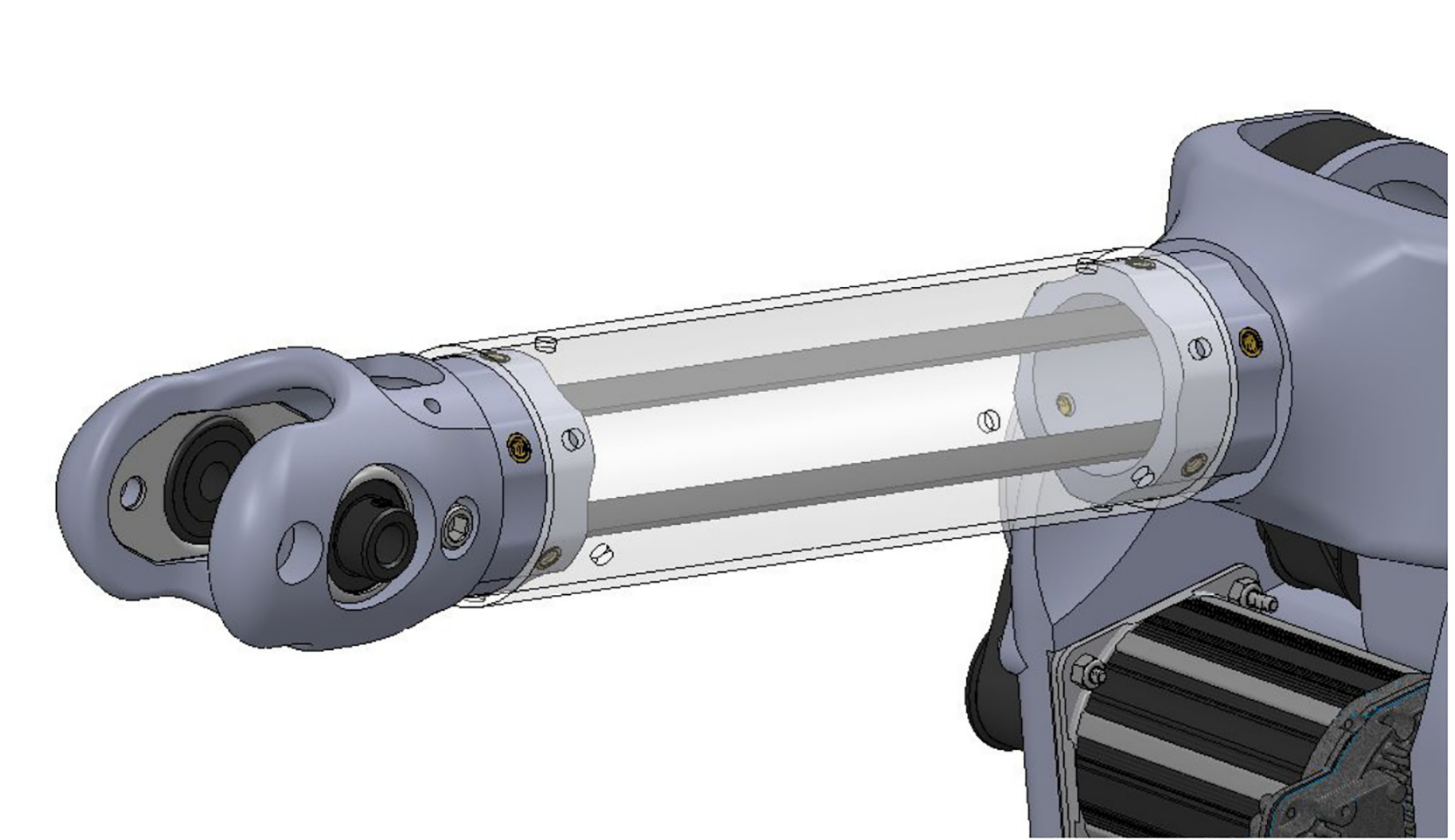

**Step 59:** Loop 13T elbow timing pulley into 42T timing belt and finish fastening mounted bearings to bicep link outboard cap.

**Figure:**
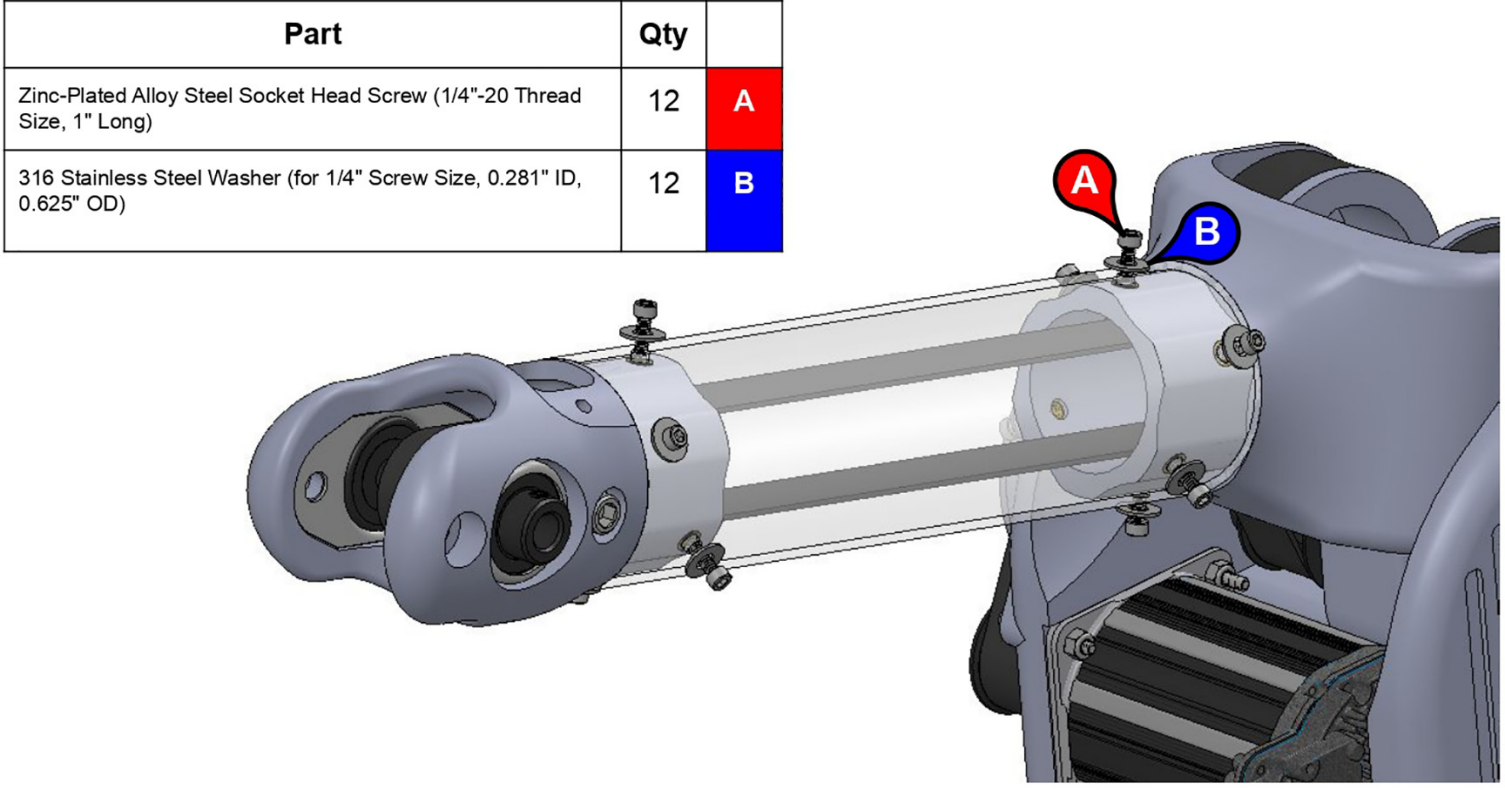

**Step 60:** Fasten elbow joint couplers to forearm link inboard cap.

**Figure:**
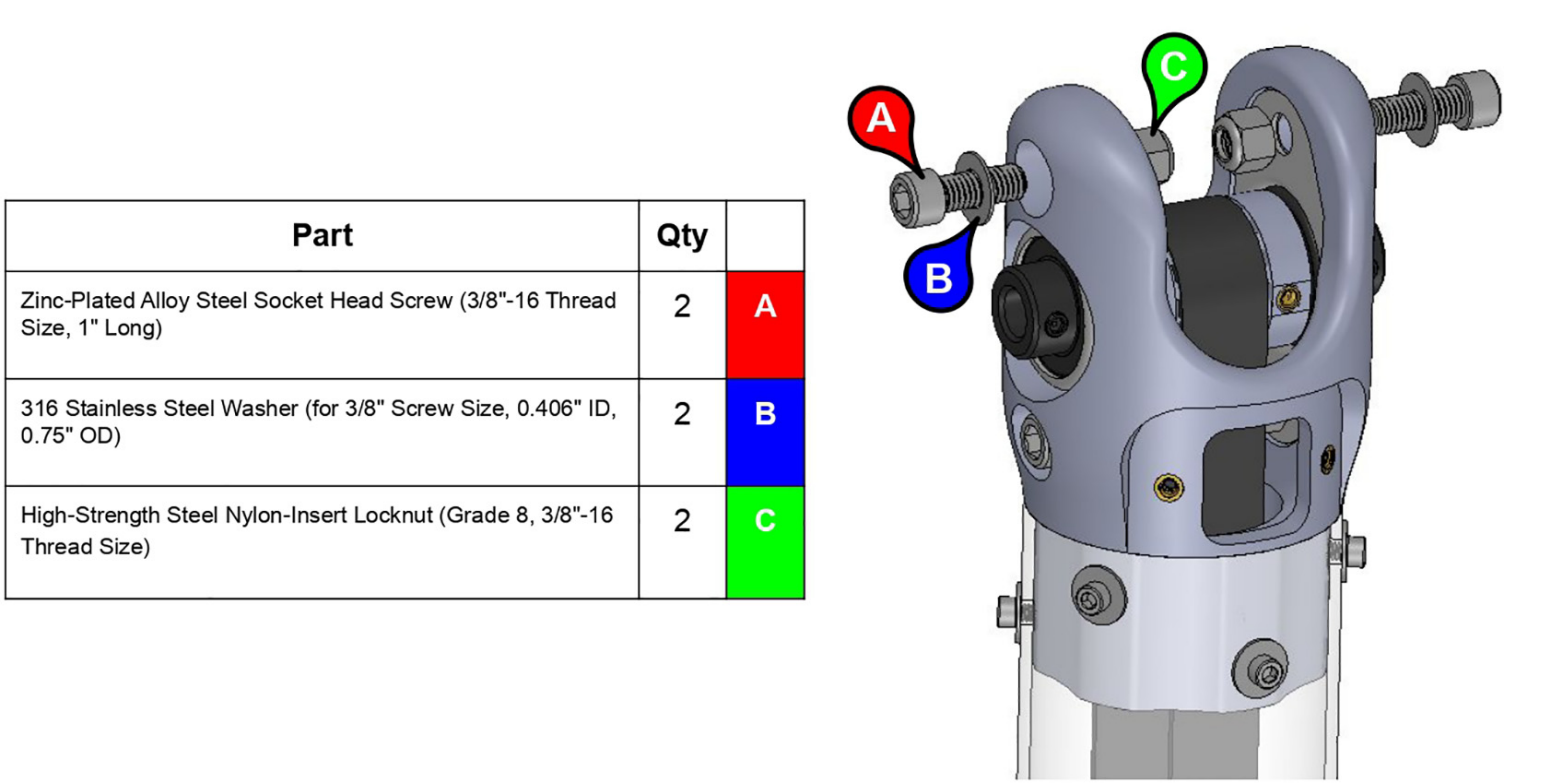

**Step 61:** Slide keyed rotary shaft through forearm link inboard cap, mounted bearings, and 13T elbow timing pulley.

**Figure:**
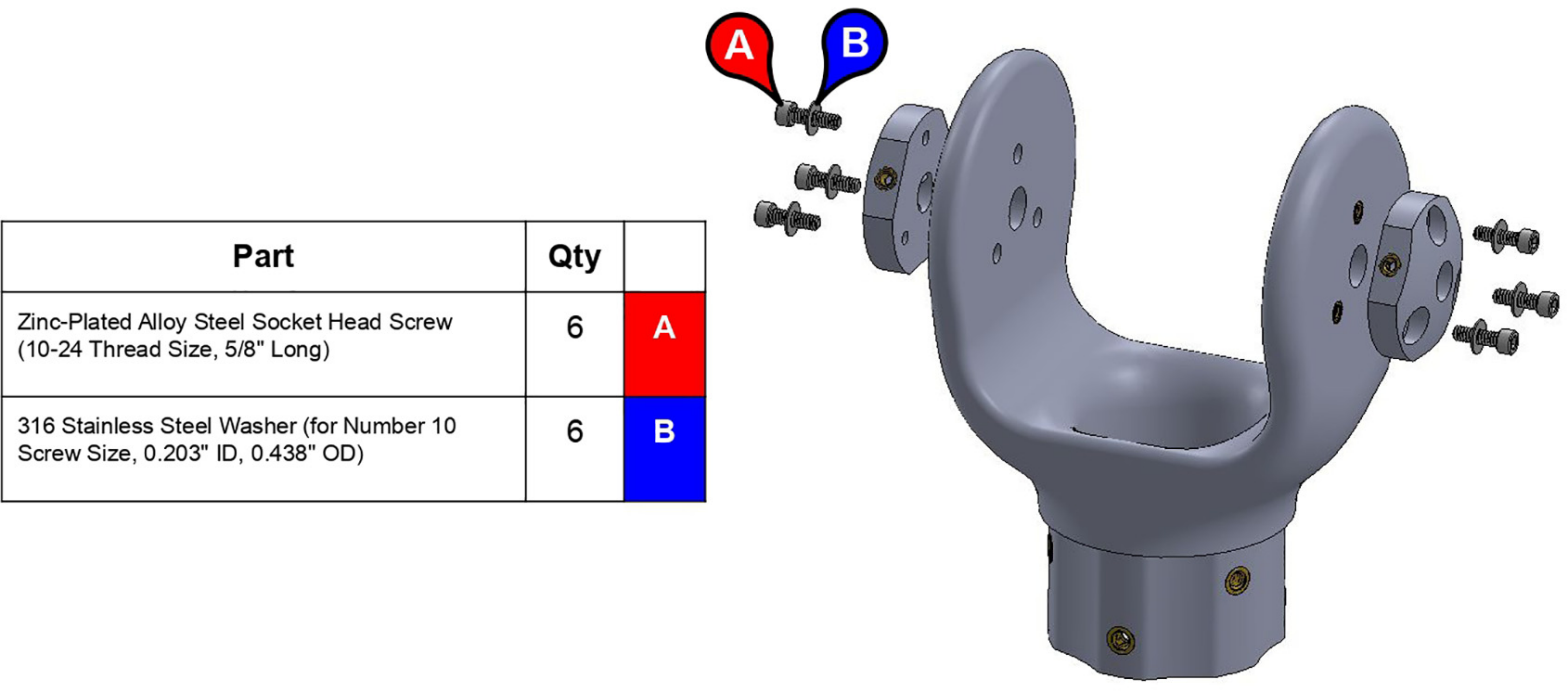

**Step 62:** Secure forearm link inboard cap to rotary shaft with set screws.

**Figure:**
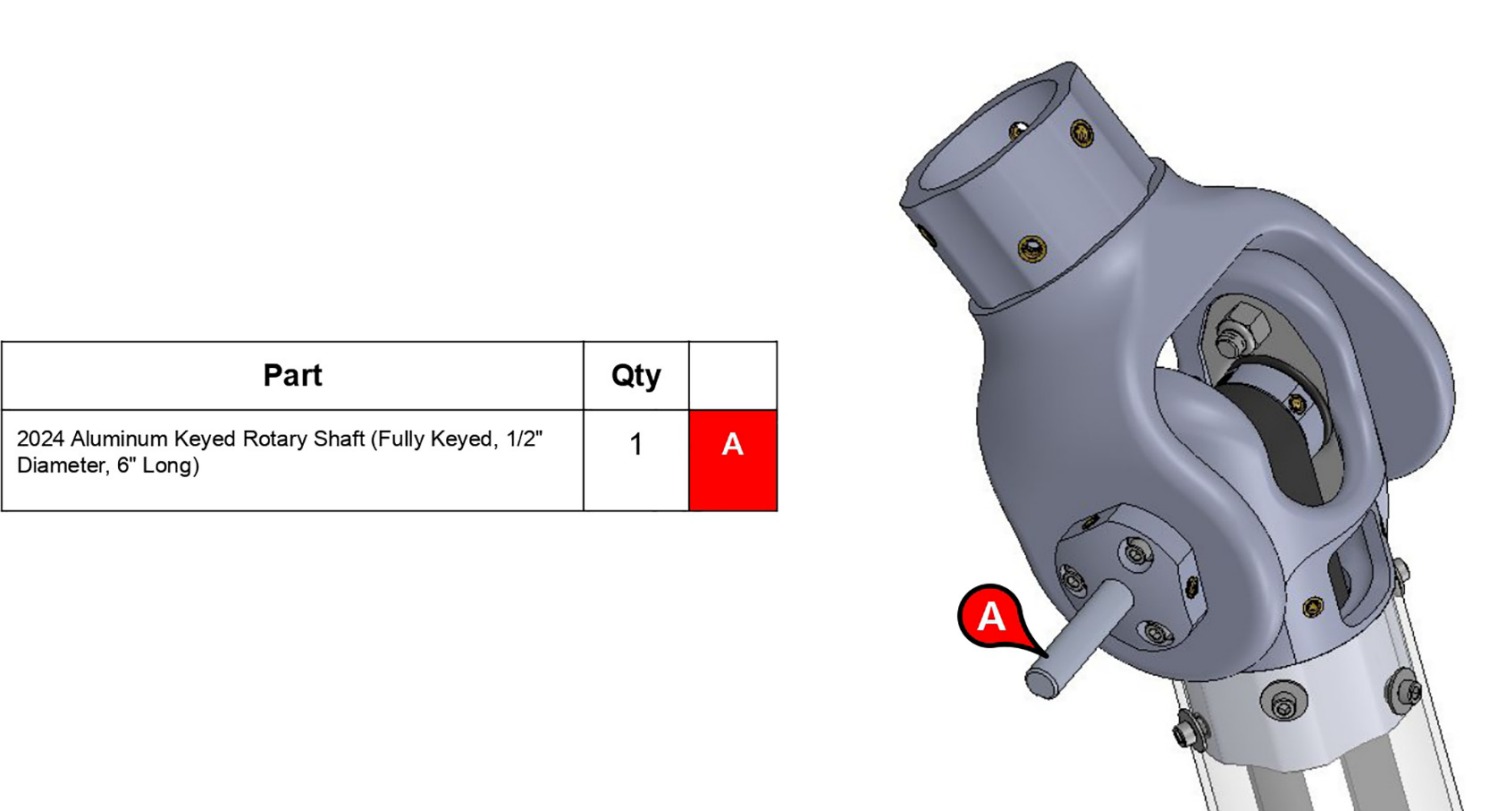

**Step 63:** Secure 13T elbow timing pulley to rotary shaft with set screws.

**Figure:**
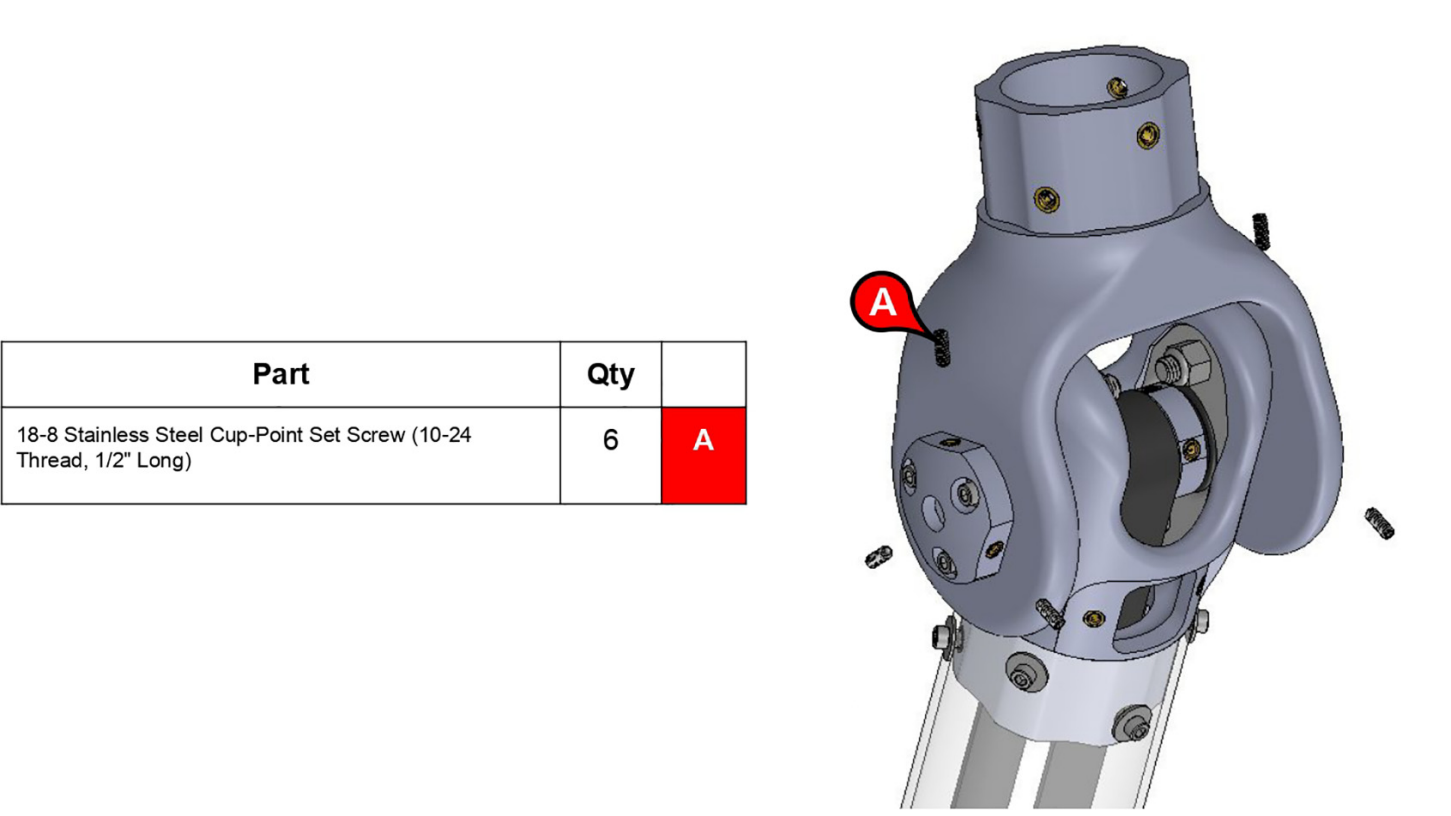

**Step 64:** Slide acrylic tube onto forearm link inboard cap.

**Figure:**
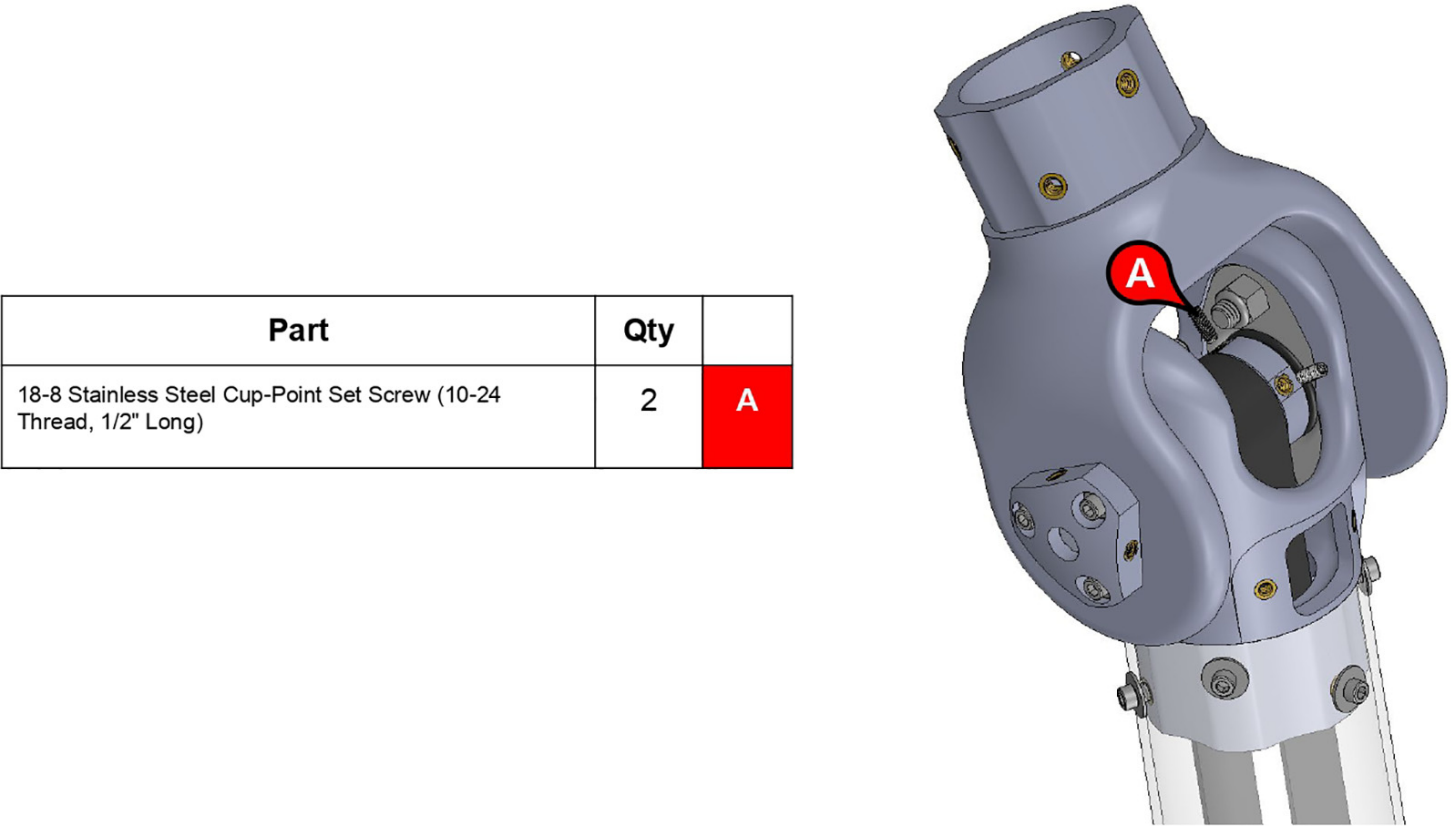

**Step 65:** Fasten acrylic tube to forearm link inboard cap.

**Figure:**
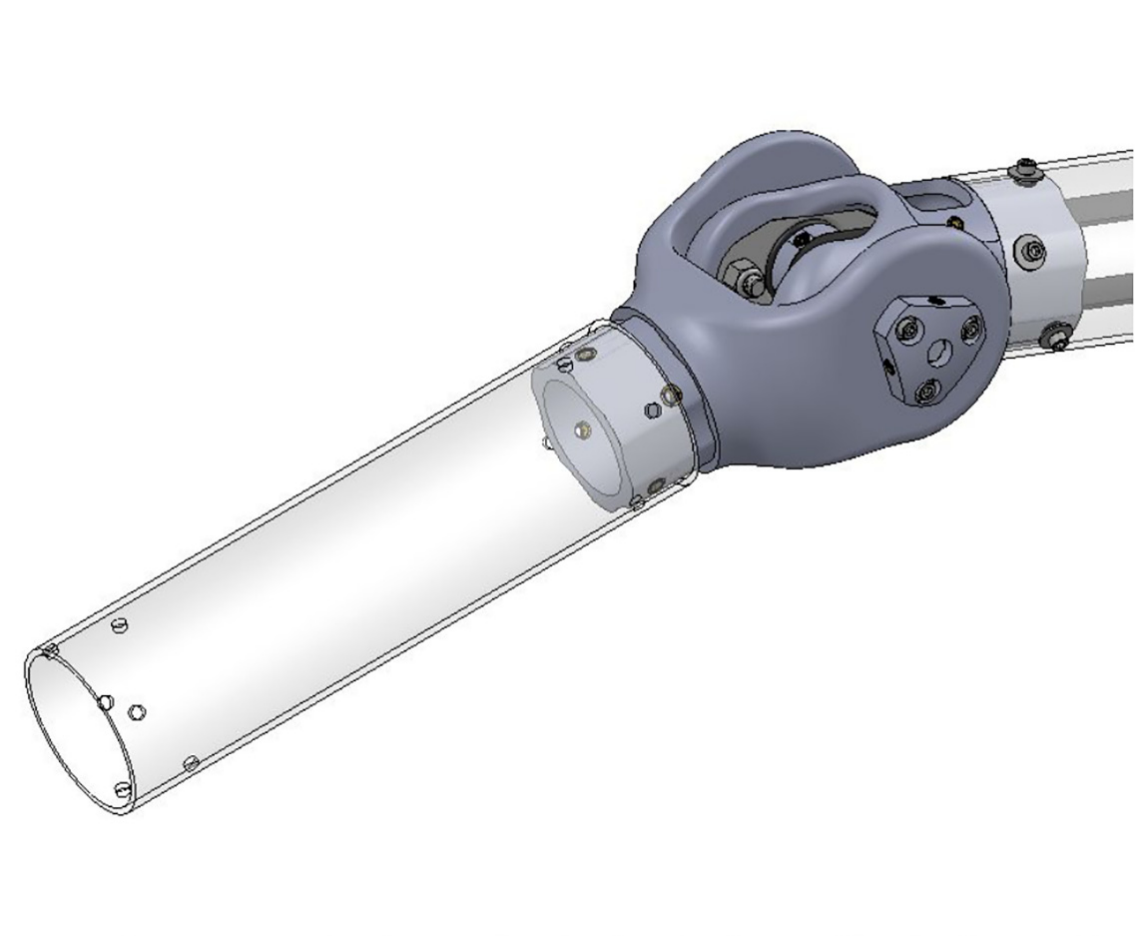

**Step 66:** Use soldering iron to set heat-set inserts into bicep link tensioner housing.

**Figure:**
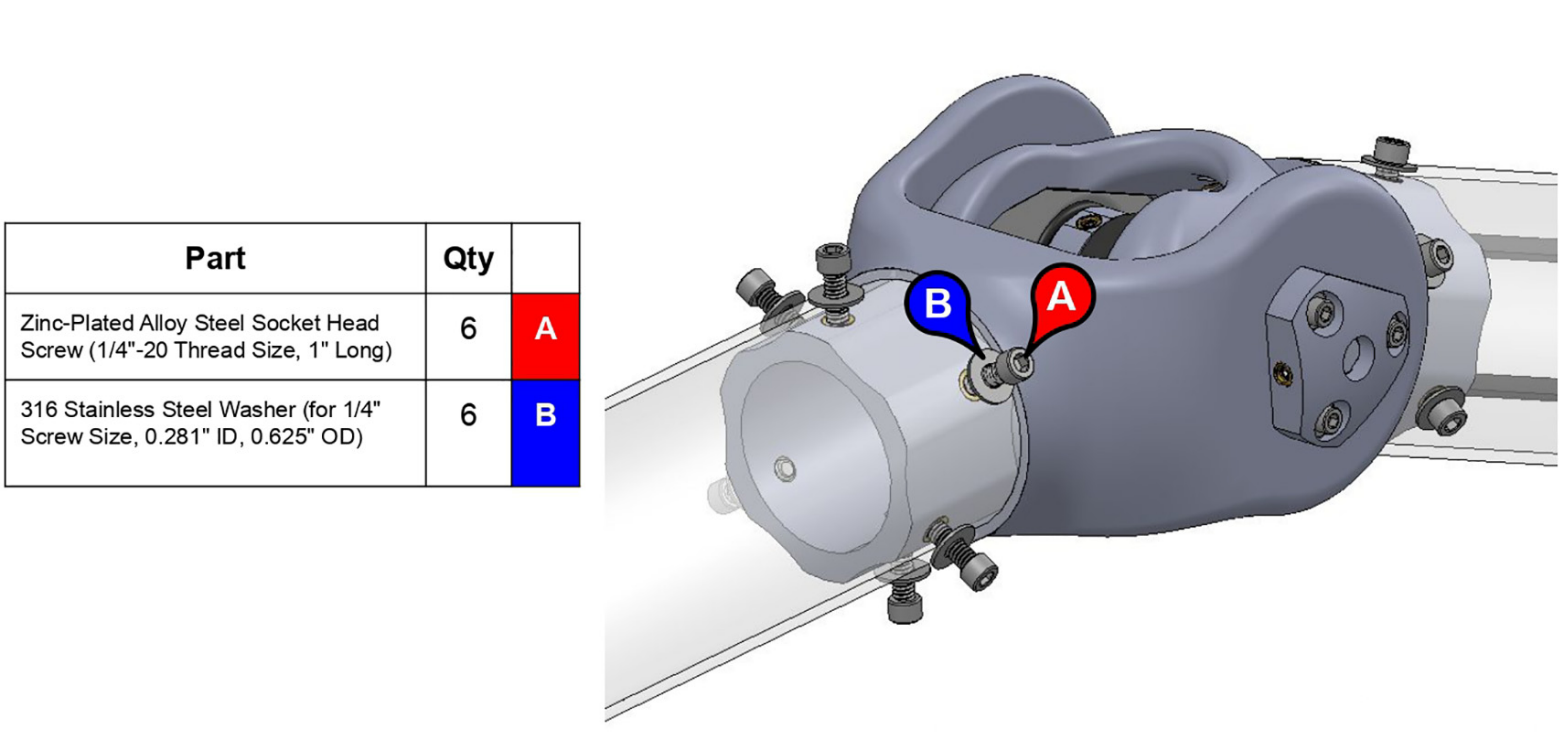

**Step 67:** Assemble various components of bicep link tensioner.

**Figure:**
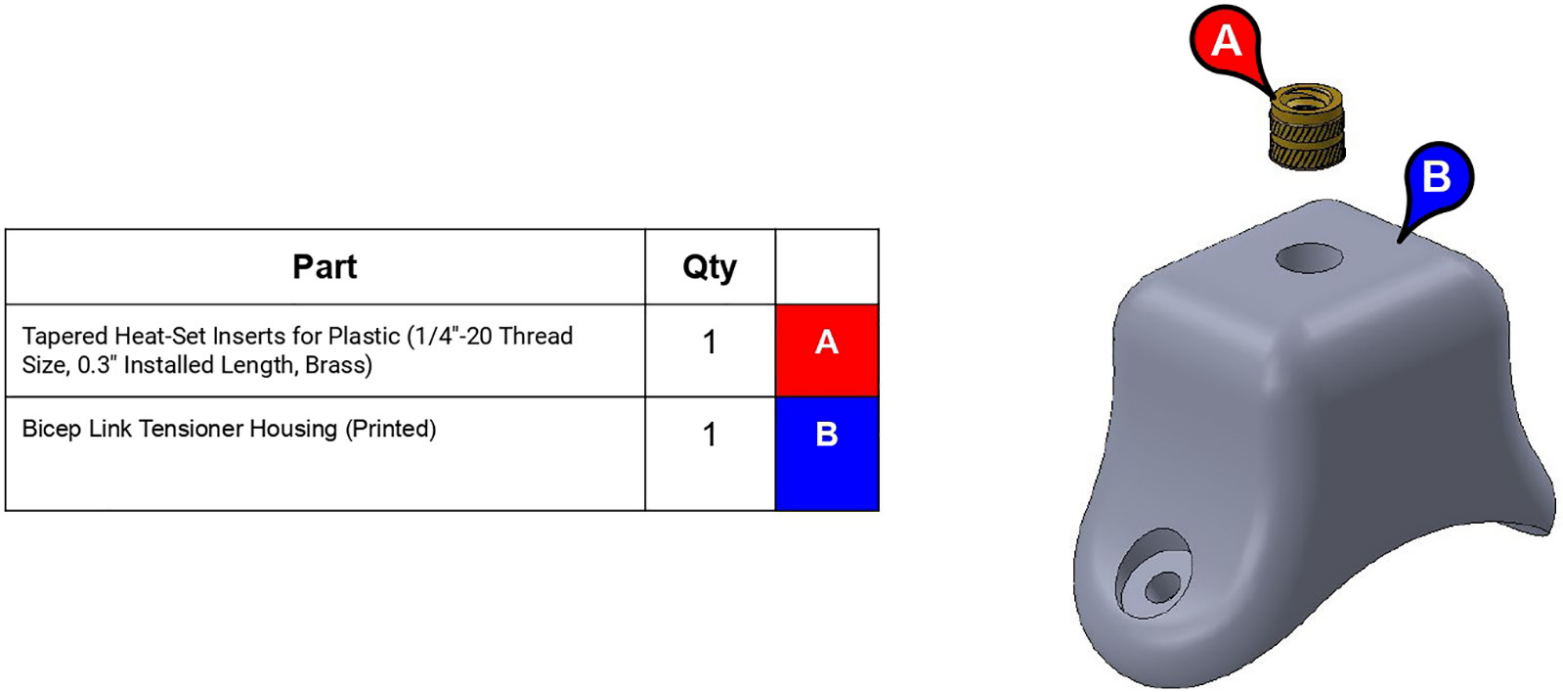

**Step 68:** Mount bicep link tensioner onto bicep link. Turn tensioner bolt to tension timing belt.

**Figure:**
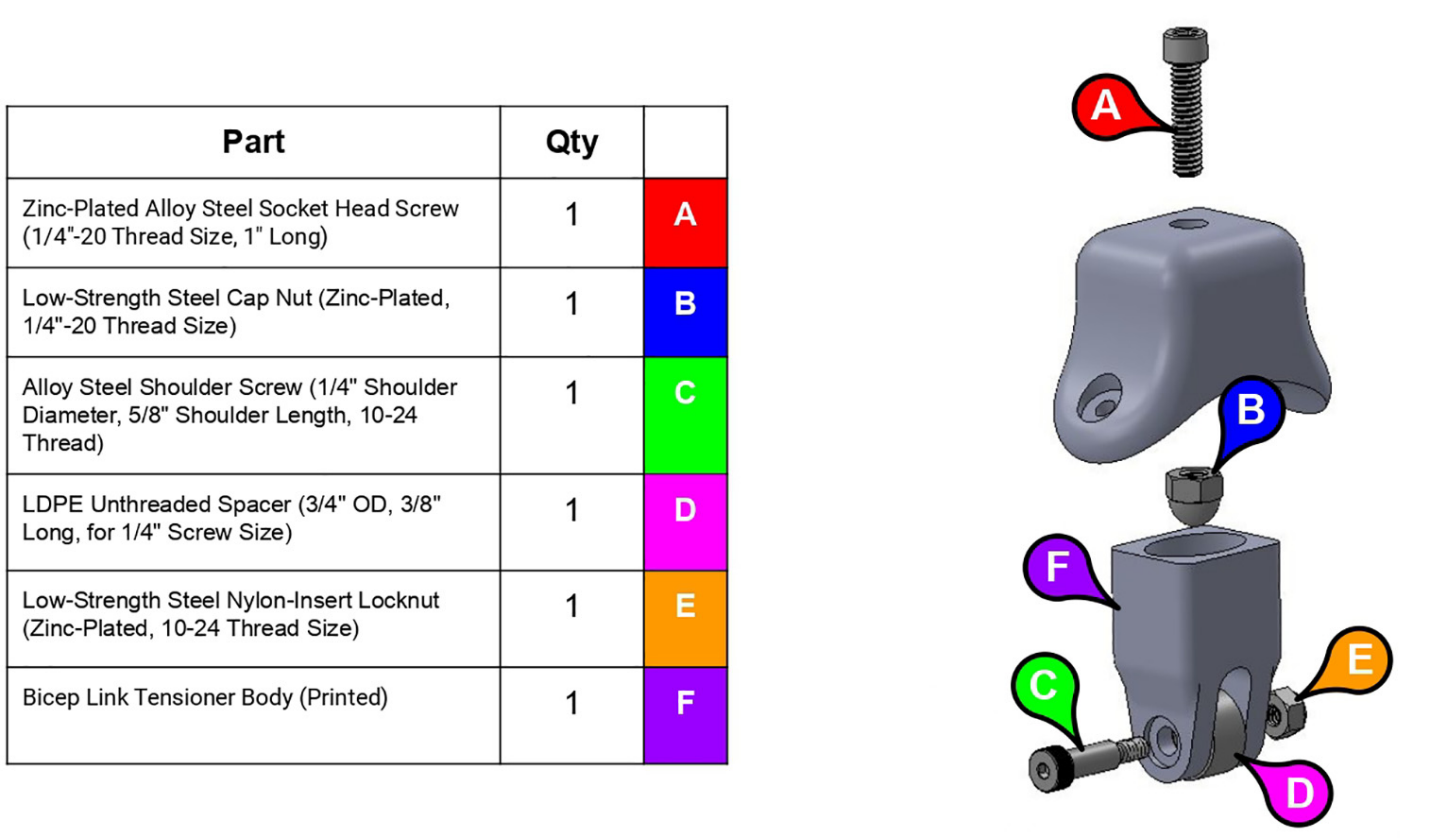

**Step 69:** Use soldering iron to set heat-set inserts into mounting plate.

**Figure:**
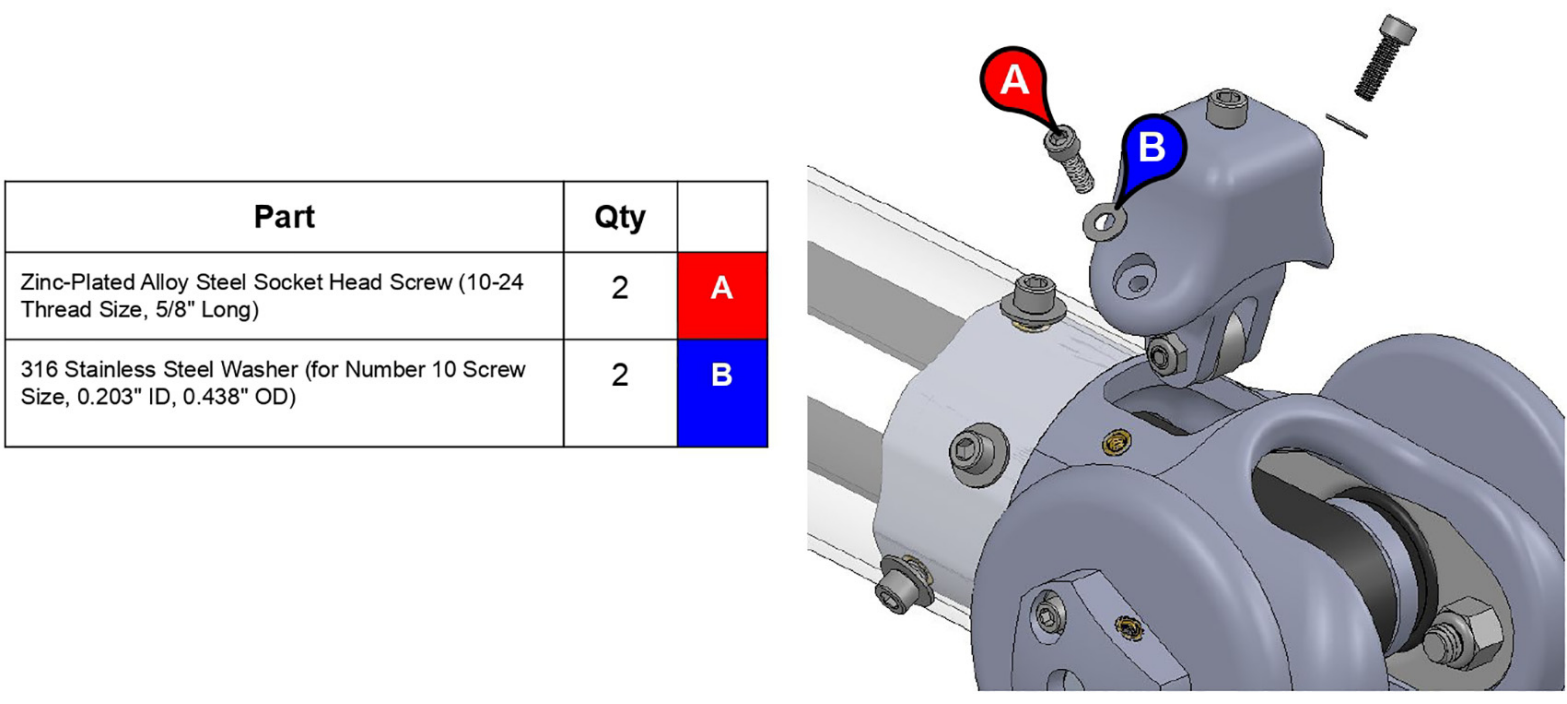

**Step 70:** Slide mounting plate into forearm link acrylic tube.

**Figure:**
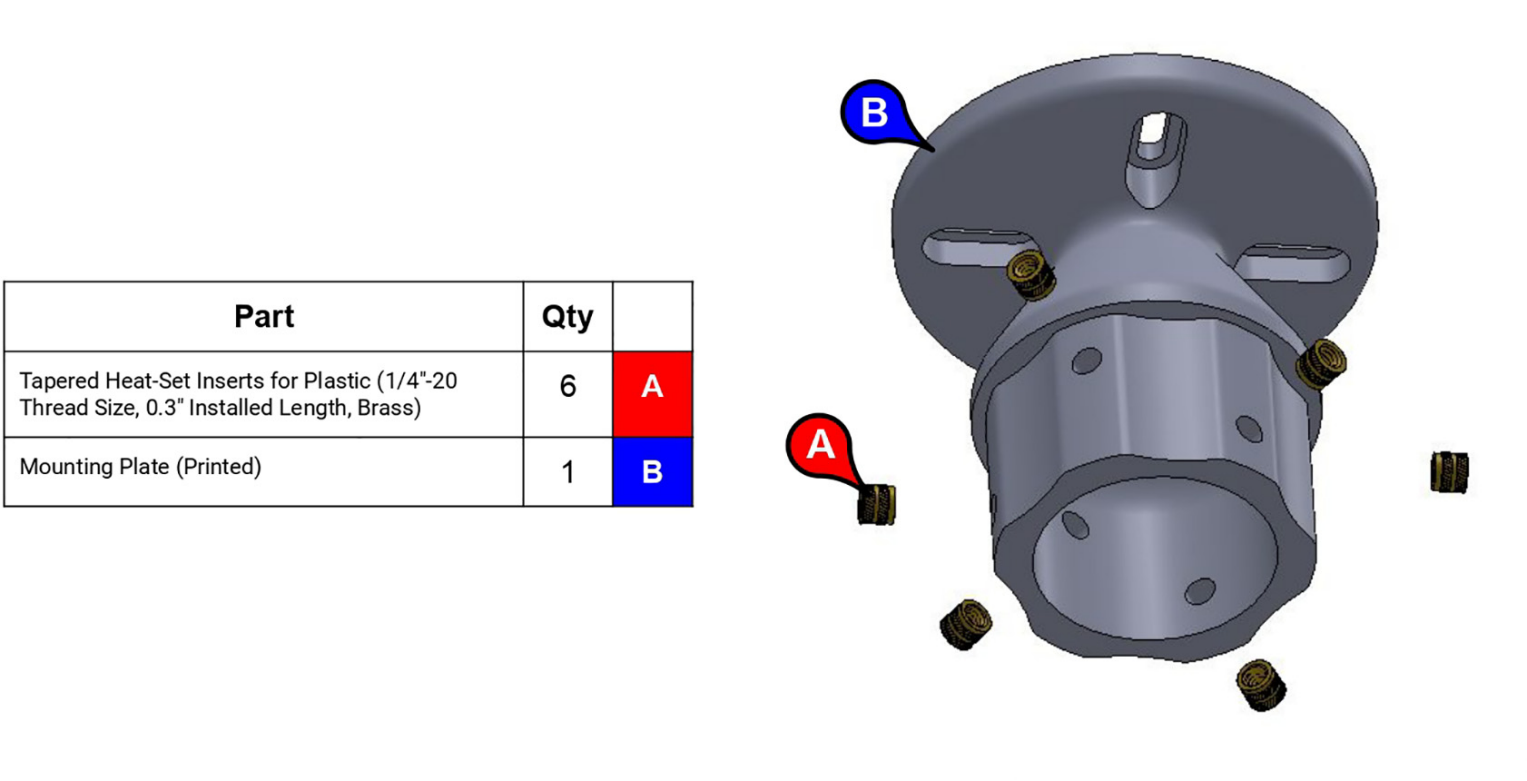

**Step 71:** Fasten mounting plate to forearm link acrylic tube.

**Figure:**
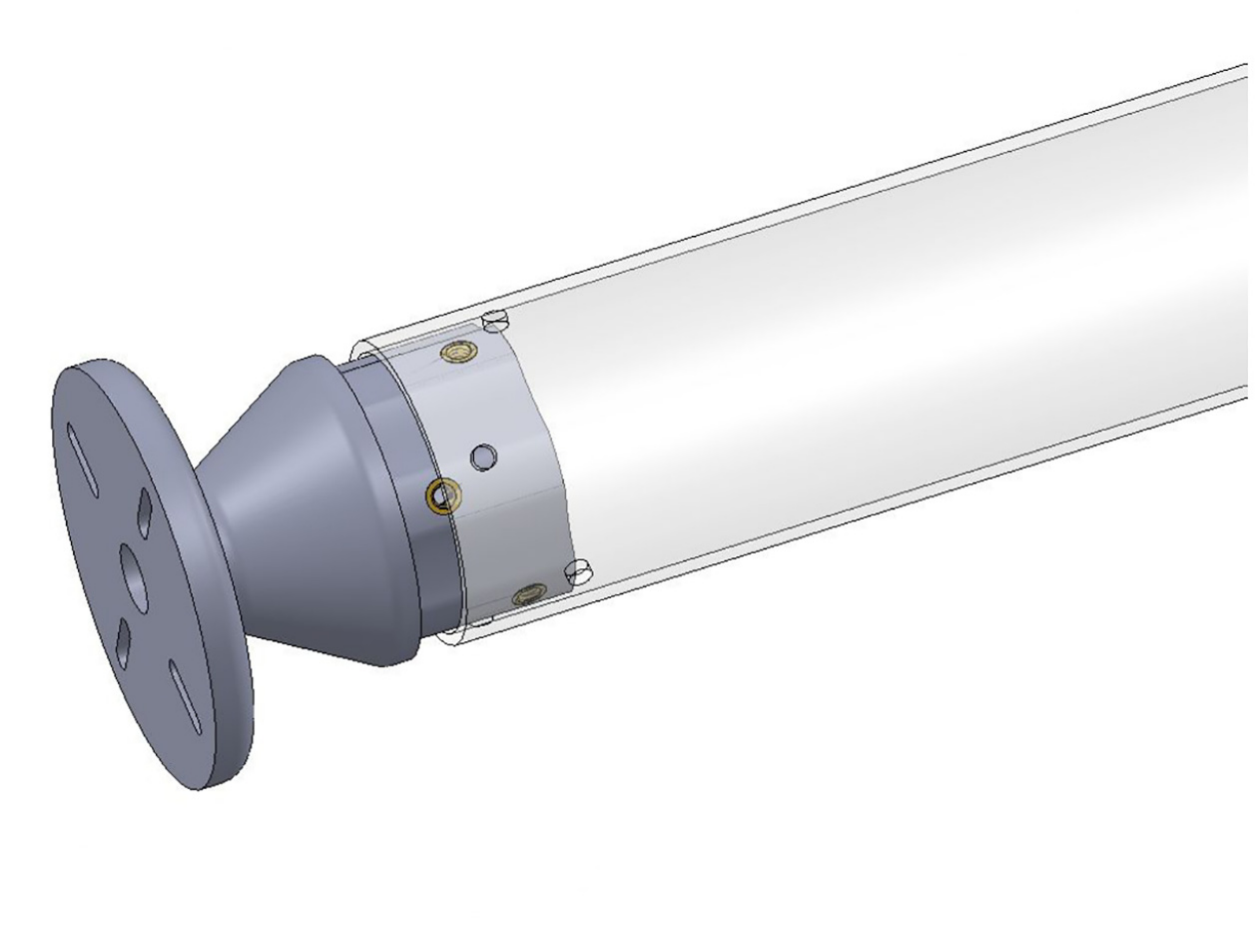

**Step 72:** Fasten power supply to power supply housing.

**Figure:**
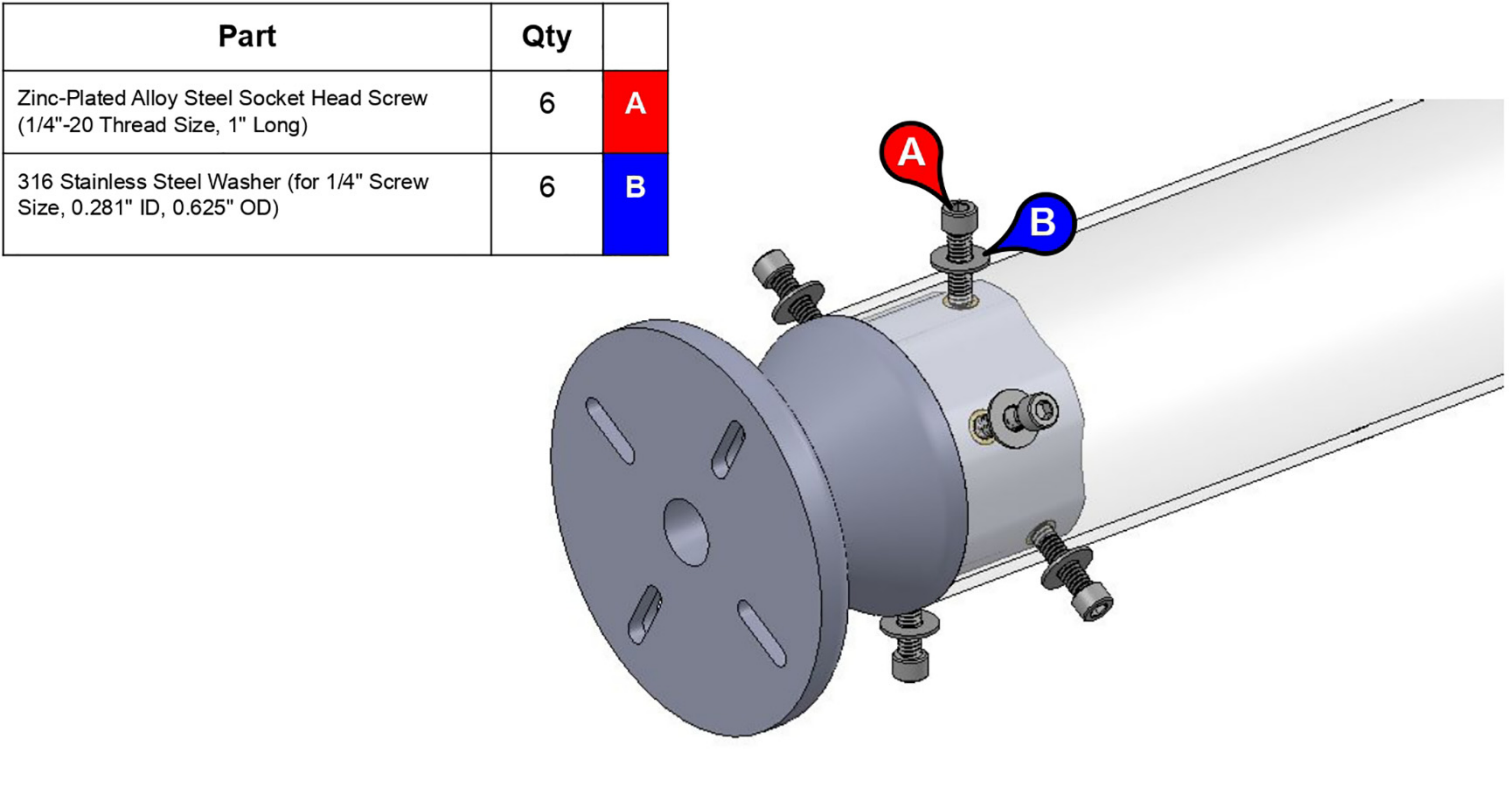

**Step 73:** Fasten Smoothieboard to Smoothieboard Housing.

**Figure:**
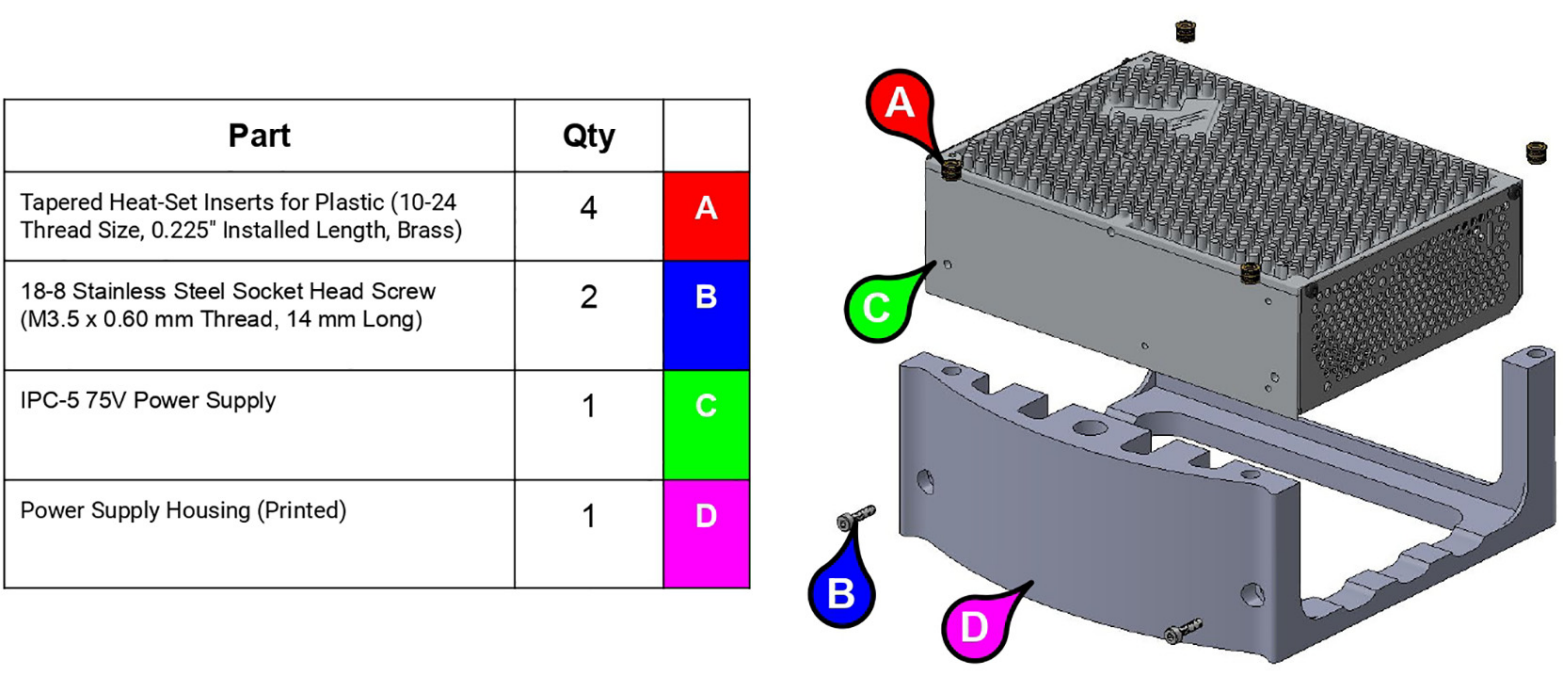

**Step 74:** Fasten power supply housing to base.

**Figure:**
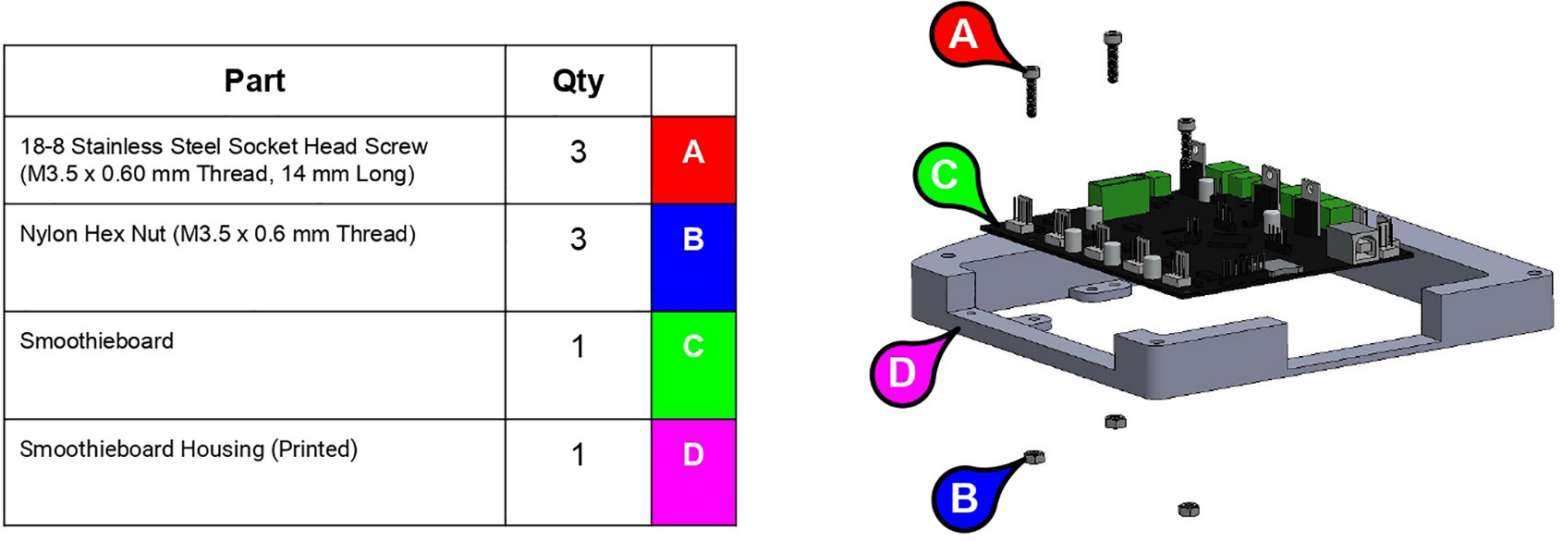

**Step 75:** Fasten Smoothieboard housing and electronics tower lid to power supply housing.

**Figure:**
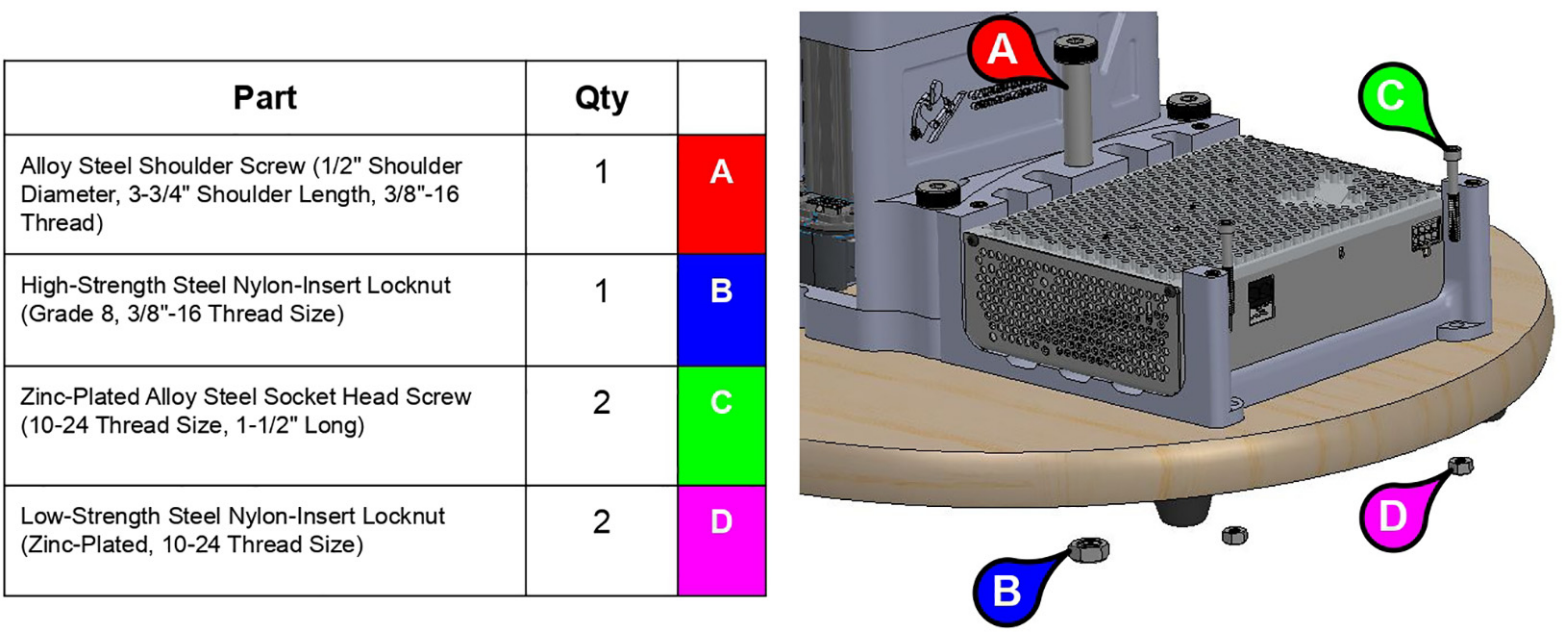

**Step 76:** Connect power supply cables between motors, power source, and wall AC power. If using the Teknic Clearpath servo system, follow the instructions provided on the Teknic website [6].

**Figure:**
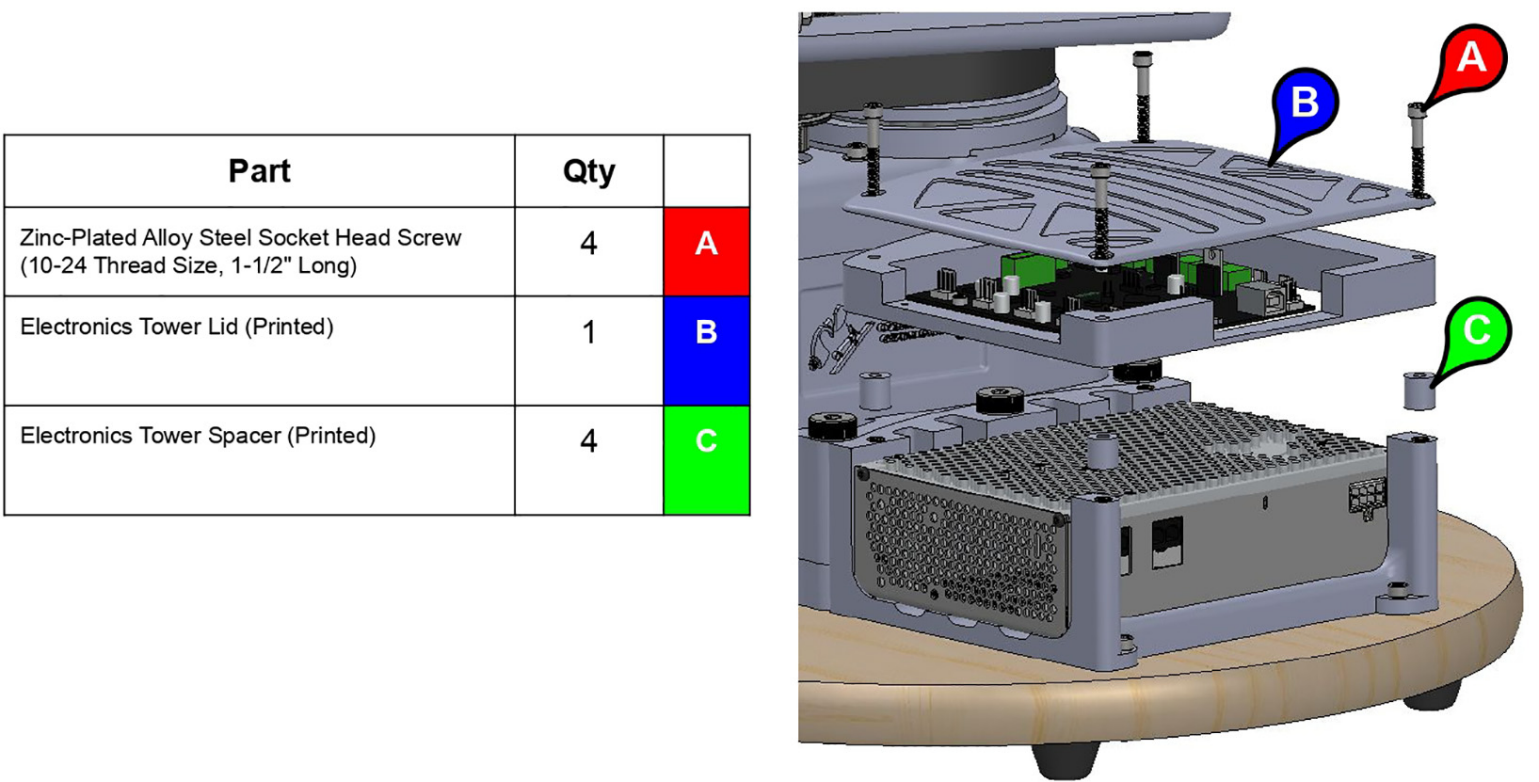

**Step 77:** Connect motor control signal wires to control board. For the provided software files, the base joint corresponds to the X-axis on the Smoothieboard, the shoulder joint the Y, and the elbow joint the Z. If using Clearpath servos/Teknic control cables and a Smoothieboard, the following wires on the cable correspond to the following pins on the Smoothieboard:

**Figure:**
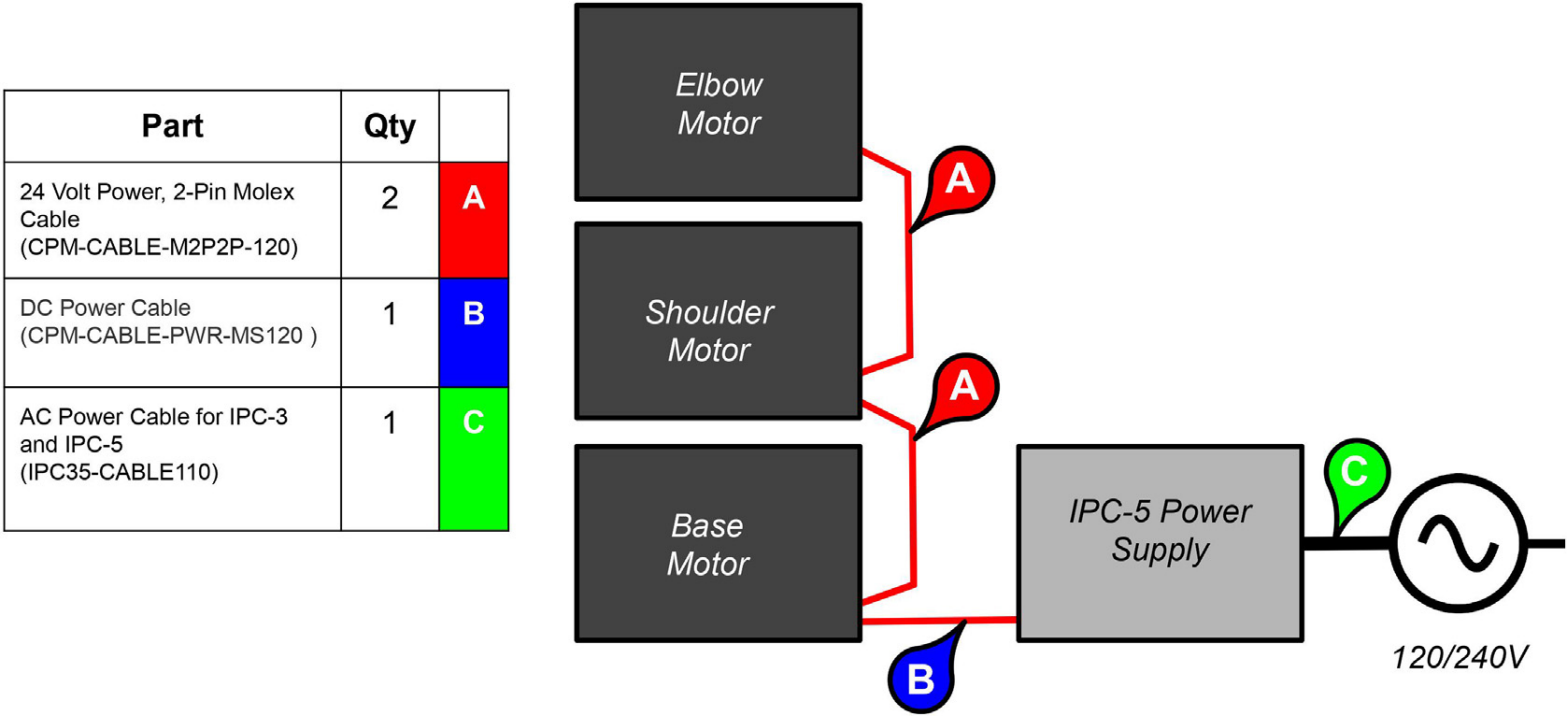

**Table:** 

**Teknic Control Cable**	**Cable Color**	**Control Board**
HLFB +	Green	N/A
Input B +	Black	ST1
Input A +	White	Dir1
Enable +	Blue	EN1
HLFB −	Red/Fuscia	N/A
Input B −	Yellow	GND
Input A −	Brown	GND
Enable −	Orange	GND

More info can be found on the Teknic [7] and Smoothieware [8] websites.

## 6 Operation instructions

### 6.1 Safety concerns

Before operating PARA, note the following safety concerns. For safety concerns pertaining to assembling PARA prior to operation, see Section 5.1.

• PARA is not designed to operate beyond the specifications presented in this article. Exceeding these specifications by, for example, attaching a heavier payload or instructing the arm to move faster than the recommended max speed, will result in PARA not functioning as intended (e.g. exhibiting jerky motions, excessive motor noise, or timing belt slippages). PARA’s components may also become permanently damaged through breaking or overheating. If PARA begins to exhibit clearly improper behaviors immediately power down the arm.
• If PARA’s components begin to smell of burnt plastic/metal or even start smoking immediately power down the arm.
• Even without an attached payload, PARA’s various hard plastic linkages can deliver a substantial amount of impact force, especially when moving at high speeds. The version of PARA presented in this paper does not contain obstacle-detection capabilities, thus if the arm encounters an object in its path of motion either PARA or the obstructing object may suffer damages. Note PARA’s workspace detailed in Fig. 6 and ensure that when the arm is powered on, any humans or animals (including the operator) are a safe distance away. Remove any unnecessary obstacles in the workspace and take note of those which must remain when controlling the arm. For operation around fragile objects consider wrapping PARA’s linkages in protective padding for extra safety.
• As a continuation of the previous point, PARA’s components are not designed to withstand high-energy or repeated impacts. The acrylic tubes used in the arm’s linkages can be brittle, while the arm’s 3D printed components can be prone to layer separation if impacted with sufficient force. Avoid high-speed collisions with obstacles and dropping PARA or any of its various components.
• PARA contains many live joints, timing belts, and toothed pulleys which could potentially pinch the user’s fingers or skin. Avoid touching or servicing these components while PARA is powered on.
• PARA’s motors utilize a considerable amount of power and can become hot after prolonged use. Exercise caution if touching these motors with bare skin during or right after using PARA.
• The Teknic Clearpath motors suggested in this article have fail safe measures to ensure that they do not draw power greater than what they – and the accompanying power supply – are rated for. If using substitute motors that do not have these features, ensure that the motors are operating within the recommended electrical specifications. Exceeding these specifications can permanently damage the motors. Also ensure that the selected power source can provide enough power for all motors at worst-case conditions. If the power source cannot supply a sufficient amount of power the arm may fail midway through performing a critical task.
• As a general safety principle for mobile robotics, when developing a new action for PARA, always test this action several times at a slower speed (the authors recommend an end effector speed of 100 mm/s) before running it at full speed.

### 6.2 Operation instructions

1. If using the proposed Teknic motors, use Teknic ClearPath-SC Series Motor Setup (Clearview) software to tune PARA’s motors under the load of the arm itself. The software can be downloaded from the Teknic website [9]. Ensure there is sufficient room around the arm for its various linkages to move without obstruction during the tuning process. Example tunes for PARA’s motors have been provided.
  Performance of stepper motors may be enchanced by tightening tensioner bolts.
  A more comprehensive guide for Teknic Clearpath motor operation can be found on the Teknic website [7].
2. If using the Smoothieboard, load the provided.config file onto the microSD card, re-insert the card back into the board, and press the ”RESET” button while the board is powered. This.config file contains correct settings for motor steps per mm travel and acceleration.
  A more comprehensive guide for Smoothieboard operation can be found on the Smoothieboard website [10].
3. Operate the arm by sending G-Code to the Smoothieboard. Many pieces of open-source software like the Printrun Suite [11] can facilitate this process. One can also communicate with and send code directly to the Smoothieboard, which is exemplified in the provided Python code.
  Below are some useful G-Code commands for operating PARA:
  • M17: Turns on all motors.
  • G91: Sets motor positioning to relative rather than absolute.
  • G92 X0 Y0 Z0: Zeros all motors.
  • G0 XN YN ZN FN: Moves X, Y, and Z motors by *N* amount at a speed of *N*.
  • M584 X0 Y1 Z2 A3 B4: If implementing additional axes in a custom end effector for example, will map additional axes to motors connected to the control board.

## 7 Validation and characterization

### 7.1 Strength assessment

**Fig. 23:**
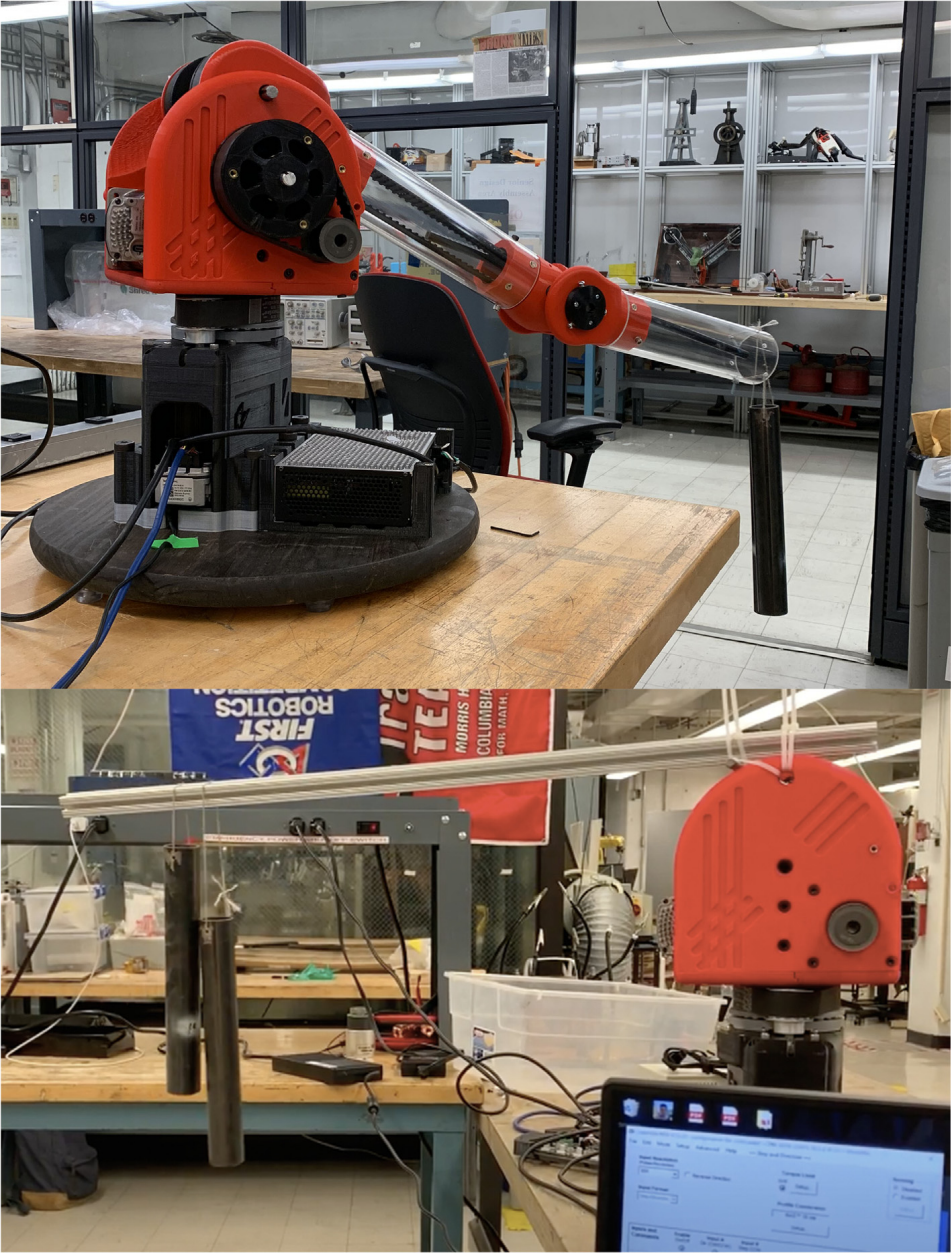
PARA lifting two 1-kg weights during an assessment of the elbow joint’s strength (top), and an early test setup for the capabilities of the base joint without the bicep and forearm links attached (bottom).

PARA’s payload-handling capabilities were assessed empirically by hanging progressively heavier weights off of the end of the arm’s forearm linkage and actuating each joint independently, noting the maximum amount of weight which could be manipulated until the joints began to behave erratically (Fig. 23).

For the base joint/central linkage axis, past approximately 2 kg of load the base joint motor began to exhibit signs that it was being pushed near its operating limits: it produced excessive vibrations, and began to turn at different rates at different angles throughout each revolution. Such motor issues were possibly exacerbated by the fact that loads on the central axle were being transmitted to the motor axle in the radial direction through the timing belt linking them (theoretically, the ball bearings around the central axle should’ve reacted any moment exerted on the axle by gravitational forces acting on the tip of the arm, but slop in the arm’s construction and deflection of the printed parts under excessive loads could easily result in a portion of these forces shifting to the axle itself). Additionally, while accelerating to high speeds (approx. 1000 mm/s end effector speed) the base joint timing belt would slip due to deformation of the belt’s rubber teeth in response to the excessive inertia of the payload.

**Fig. 24:**
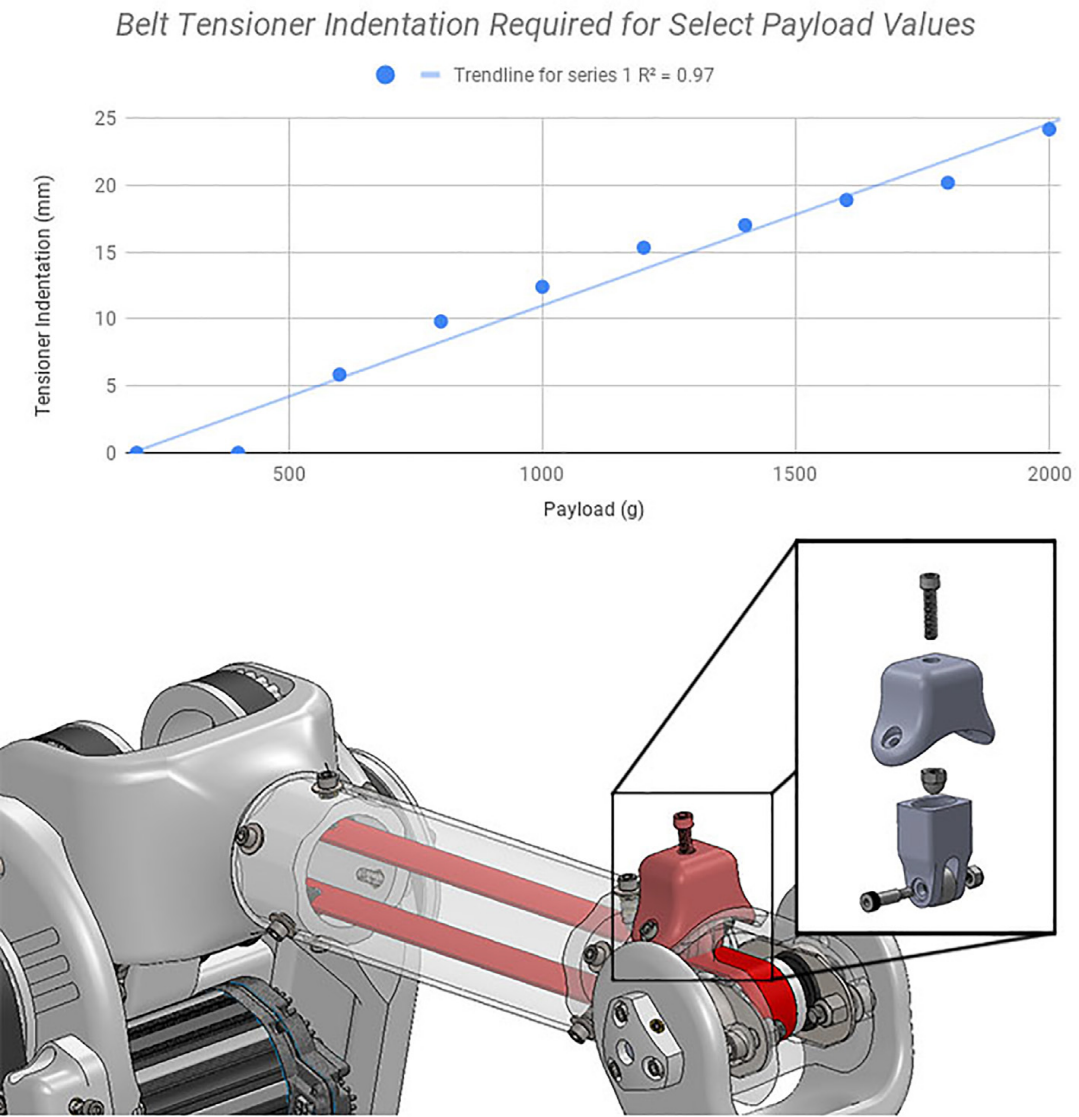
Indentation of elbow joint timing belt tensioner (highlighted in red in CAD screenshot) required to lift select payload values without belt slippage. Elbow joint velocity and acceleration values used in each test were approximately 3 rad/s and 3 rad/s^2^, respectively. Note the interestingly linear relationship (R^2^ =.97).

For the shoulder and elbow axes actuating the bicep and forearm linkages, the timing belts would also begin to slip past 2 kg of load, mostly due to excessive gravitational force. Tensioning the timing belts assisted in preventing belt slippage to a certain degree (most prominently in the long 42-tooth timing belt linking the final two pulley gears in the elbow joint timing belt system, as shown in Fig. 24), but excessive belt tensions risked breaking the arm’s plastic parts (either through deforming them through excessive bending moments, cracking the parts, or separating the parts’ 3D printed layers) and damaging the motors through excessive radial thrust forces on their axles.

Stronger, more robust materials for the structural elements of PARA such as metal or solid injection-molded plastic parts would undoubtedly improve the payload capabilities of the arm by minimizing part deflection and unwanted load paths in the assembly. However, such parts would also prompt much higher material and tooling costs (part-specific injection/casting molds and stamping dies, for example), which is a trade-off PARA makes to drive down cost. Automated means of assembling PARA could also potentially allow for extremely tensioned timing belts (much tighter than what the average person could assemble by hand) to prevent belt slippage, but such means of assembly would also incur higher costs.

**Fig. 25:**
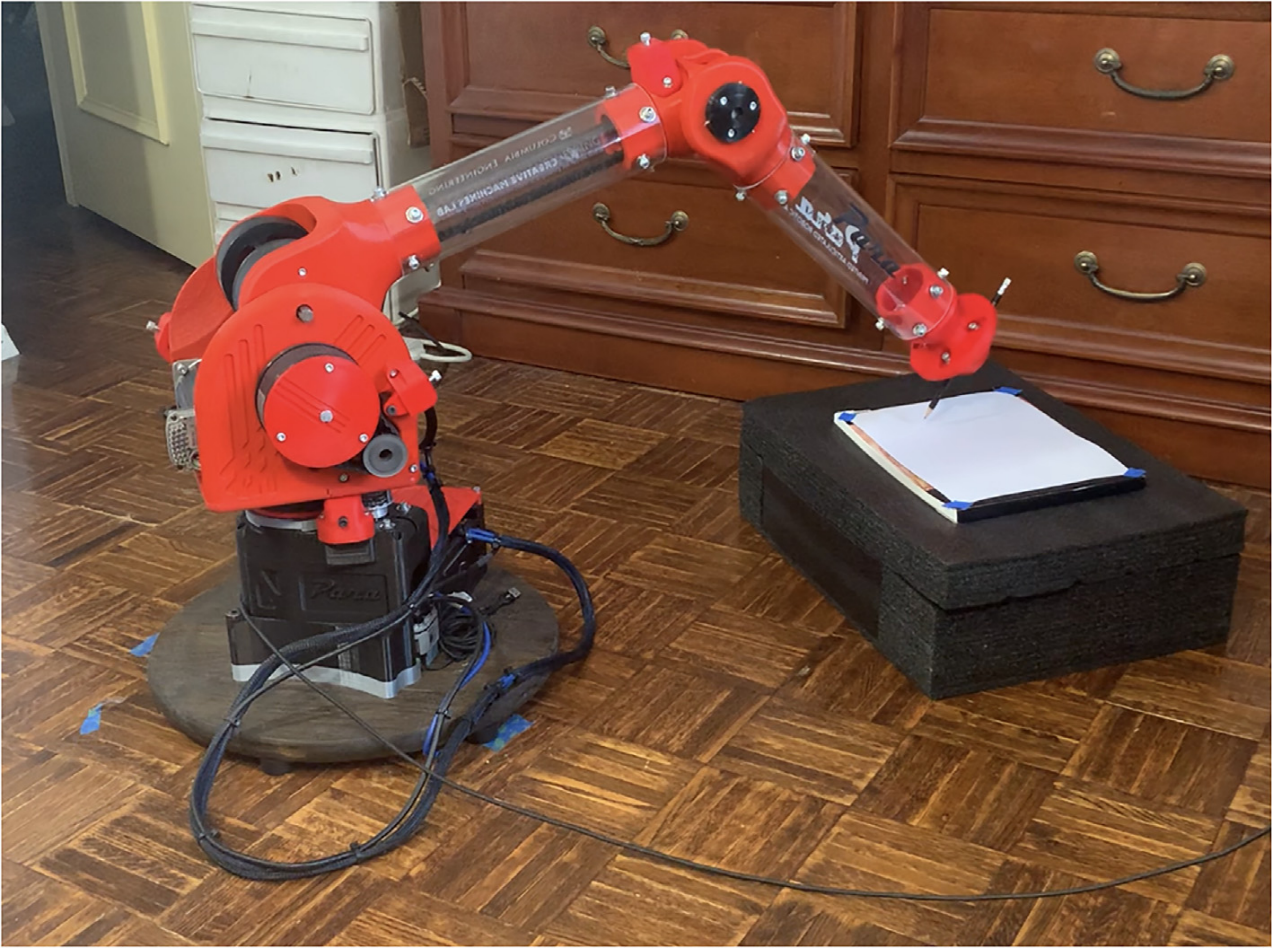
PARA performing precision assessment.

**Fig. 26:**
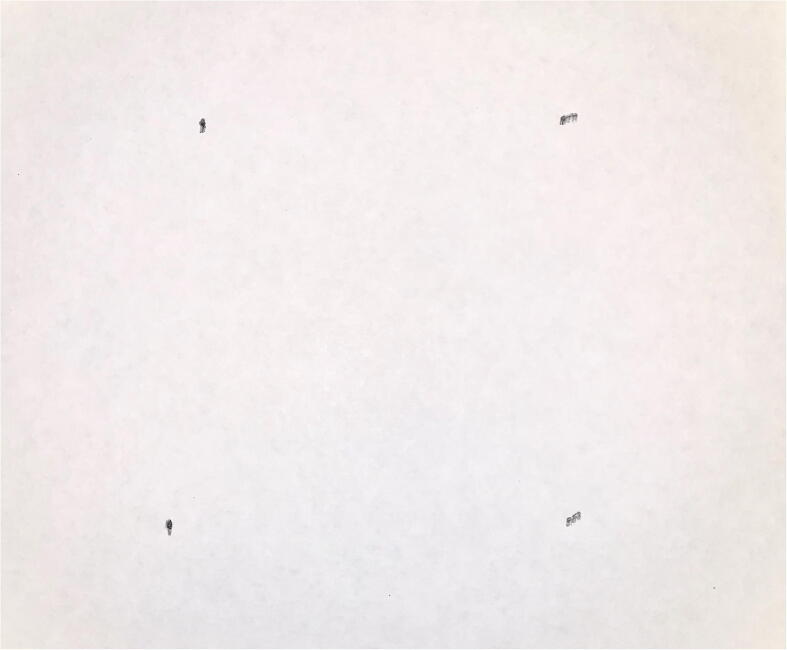
Markings from 250 mm/s assessment.

**Fig. 27:**
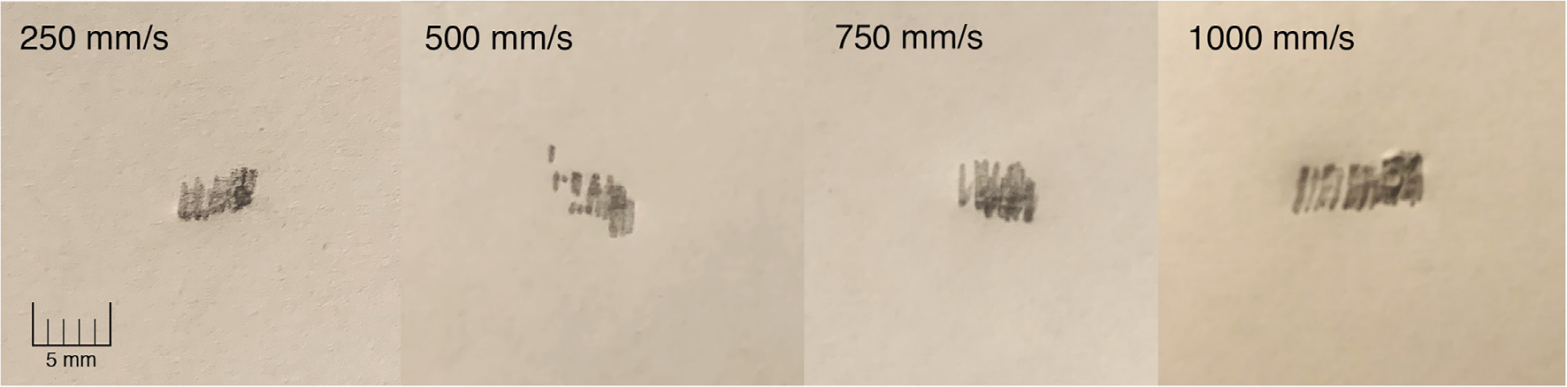
Detail view of upper right corner from various assessments. Note scale and differing proximity of points to one another.

When performing these strength assessments for each joint, maximum joint acceleration values which would also allow each degree of freedom to operate reliably at a variety of payloads were chosen. These values were about 3 rad/s^2^ for the base and shoulder joints and 6 rad/s^2^ for the elbow joint.

Running the theoretical maximum payload calculations from Section 2.6 while assuming operating accelerations of 3 rad/s^2^ for the base and shoulder joints and 6 rad/s^2^ for the elbow joint yields values of 2.1 kg for the base joint, 7.7 kg for the shoulder joint, and 15.8 kg for the elbow joint. The theoretical base joint max load aligns with the observations from this study: the corresponding motor was indeed operating at values close to its peak torque while manipulating the 2 kg payload. The large discrepancies in the shoulder and elbow joints’ theoretical max payload and actual max payload can be accounted for by the points of mechanical failure discussed throughout this section (there is also more potential for power loss and sources of failure for these axes due to the more elaborate nature of their power transmission mechanisms). Future work with PARA should consist of finding methods to increase the tension in PARA’s timing belts while distributing these forces in a mechanically sound way.

**Table:** 

**Axis**	**Acceleration (rad/s**^**2**^**)**	**Theoretical Max Payload (kg)**	**Actual Max Payload (kg)**	**Efficiency**
Base ($\theta_{X}$)	3	2.1	2.0	95%
Shoulder ($\theta_{Y}$)	3	7.7	2.0	26%
Elbow ($\theta_{Z}$)	6	15.8	2.0	13%

### 7.2 Repeatability assessment

PARA’s repeatability was assessed by mounting a 0.7 mm mechanical pencil to the end effector, repeatedly having the arm dot four corners on a 100 mm by 100 mm square (alternating between different corners of the square), and measuring the range of pencil marks produced at each of those four corners (Fig. 25, Fig. 26, Fig. 27). Four assessments were conducted, each with the end effector traveling at a different cartesian velocity: 250 mm/s, 500 mm/s, 750 mm/s, and 1000 mm/s. For each assessment, 36 points were produced at each corner of the square (meaning 144 data points were collected for each assessment). The end effector’s cartesian acceleration for each test was 250 mm/s^2^.

The geometric mean of the x and y ranges for each corner in the assessments were as follows:

**Table:** 

**Speed (mm/s)**	**Top Left Range (mm)**	**Top Right Range (mm)**	**Bottom Left Range (mm)**	**Bottom Right Range (mm)**
250	4.47	6.40	5.83	3.61
500	5.83	7.07	7.07	5.83
750	10.30	7.07	6.40	5.66
1000	10.30	11.18	10.77	9.85

Using Student’s t-distribution and assuming 35 degrees of freedom for each sample set, the standard deviation for each corner was as follows:

**Table:** 

**Speed (mm/s)**	**Top Left SD (mm)**	**Top Right SD (mm)**	**Bottom Left SD (mm)**	**Bottom Right SD (mm)**
250	2.65	3.79	3.45	2.13
500	3.45	4.18	4.18	3.45
750	6.09	4.18	3.79	3.35
1000	6.09	6.62	6.37	5.83

Note that two assumptions were made in obtaining these values: 1) because the points at each corner were so close to each other, it was impractical to measure the actual distribution of the points from the mean, and it’s assumed that all points conform to Student’s t-distribution, and 2) the measured ranges at each corner encompass 95% of all points at those corners.

Treating each corner as a sample set from a total population (the population being all points corresponding to a certain end effector velocity), the pooled standard deviation and range with 95% confidence for each assessment are as follows:

**Table:** 

**Speed (mm/s)**	**Total SD (mm)**	**Total Range at 95% Confidence (mm)**	**Repeatability (mm)**
250	3.07	5.20 mm	±2.60
500	3.83	6.48 mm	±3.24
750	4.48	7.57 mm	±3.79
1000	6.23	10.54 mm	±5.26

**Fig. 28:**
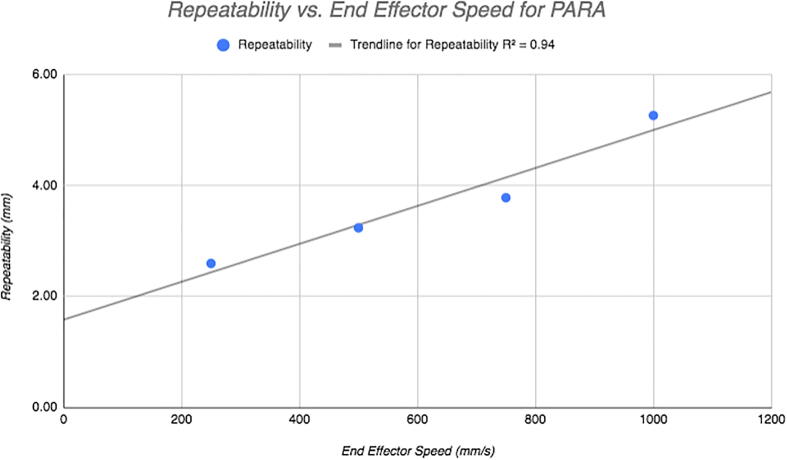
Repeatability demonstrated by PARA during various trials with different end effector speeds. Note trendline.

Interestingly, at this range of end effector speeds, end effector speed and repeatability display a linear relationship, with an R-squared value of 0.94 obtained when plotting the two variables together (Fig. 28).

It must be stressed that these values were obtained with no payload attached to the end effector; repeatability and precision can be assumed to decrease as the arm payload increases.

## Human and animal rights

No humans or animals were harmed in this study.

## CRediT authorship contribution statement

**Albert Tai:** Conceptualization, Methodology, Software, Validation, Formal analysis, Investigation, Writing - original draft, Writing - review & editing, Visualization. **Michael Chun:** Conceptualization, Methodology, Investigation, Writing - original draft. **Yuqiu Gan:** Software, Validation, Writing - original draft, Writing - review & editing. **Mert Selamet:** Methodology, Investigation, Writing - review & editing. **Hod Lipson:** Conceptualization, Resources, Writing - review & editing, Supervision, Project administration, Funding acquisition.

## Declaration of competing interest

The authors declare that they have no known competing financial interests or personal relationships that could have appeared to influence the work reported in this paper.

## Figures and Tables

**Table:** 

**Name/Source**	**DoF**	**Payload (kg)**	**Repeat-ability (mm)**	**Reach (mm)**	**Cost**	**No. in**Fig. 1	**No. in**Fig. 3
UFACTORY uArm Swift Pro [6]	4	0.5	0.2	320	749	1	1
Thor [8]	6	0.75	?	422.15	379	2	2
BCN3D MOVEO [1]	5	0.8	?	538	400	3	3
LittleArm BIG [17]	5	0.11	?	292	119	4	4
3D Printable Robot Arm (A. Hoelldorfer) [18]	6	2	?	600	?	5	N/A
Zortrax Robotic Arm [19]	5	0.1	?	378	?	6	N/A
ViperX 250 Robot Arm [3]	5	0.45	1	650	3795.95	7	5
ReactorX 200 Robot Arm [2]	5	0.15	2.5	550	1695.95	8	6
Niryo One - 6-Axis Robotic Arm [11]	6	0.3	1	440	1999	9	7
RobotDigg SCARA [20]	6	2	0.1	650	3890	10	8
RobotAnno V6PLUS [21]	6	1	0.5	480	1390	11	9
Innfos Gluon [22]	6	1	?	421	579	12	10
Tinkerkit Braccio [23]	5	0.4	?	800	218	13	11
xArm 5 Lite [24]	5	3	0.1	700	5068	14	12
Meca500 [25]	6	0.5	0.005	260	15000	15	N/A
Fanuc ARC Mate 50iD/7L [26]	6	7	0.03	911	>25000	16	N/A
Fanuc LR Mate 200iD [10]	6	7	0.01	717	>25000	17	N/A
Fanuc LR Mate 200iD/4S [27]	6	4	0.01	550	>25000	18	N/A
Fanuc LR Mate 200iD/4SH [28]	5	4	0.01	550	>25000	19	N/A
Fanuc LR Mate 200iD/7H [29]	5	7	0.01	717	>25000	20	N/A
Fanuc LR Mate 200iD/7L [30]	6	7	0.01	911	>25000	21	N/A
Fanuc M-10iA/12S [31]	6	12	0.01	1098	>25000	22	N/A
Fanuc M-10iA/10 M [32]	6	10	0.01	1422	>25000	23	N/A
KUKA Agilus KR 3 R540 [33]	6	3	0.02	541	24000	24	N/A
KUKA Agilus KR 4 R600 [34]	6	4	?	600	23000	25	N/A
KUKA Agilus KR 10 R1100-2 [35]	6	11.1	0.02	1101	33000	26	N/A
KUKA LBR iiwa 7 R800 [36]	7	7	.1	800	77000	27	13
UR3e [7]	6	3	?	500	>25000	28	N/A
UR5e [37]	6	5	?	850	>25000	29	N/A
UR10e [38]	6	10	?	1300	>25000	30	N/A
UR16e [38]	6	5	?	900	>25000	31	N/A
RobotAnno RS900 [39]	6	5	0.05	900	?	32	N/A
PARA	3	2	?	876	3400	33	13

## Appendix: Robotic arm models consulted for hardware in context

Note: ? = Not provided by source.
